# Design, synthesis, and biochemical and computational screening of novel oxindole derivatives as inhibitors of Aurora A kinase and SARS-CoV-2 spike/host ACE2 interaction

**DOI:** 10.1007/s00044-024-03201-7

**Published:** 2024-03-05

**Authors:** Donatus B. Eni, Joel Cassel, Cyril T. Namba-Nzanguim, Conrad V. Simoben, Ian Tietjen, Ravikumar Akunuri, Joseph M. Salvino, Fidele Ntie-Kang

**Affiliations:** 1 Center for Drug Discovery, Faculty of Science, University of Buea, Buea, Cameroon; 2 Department of Chemistry, Faculty of Science, University of Buea, Buea, Cameroon; 3 The Wistar Institute, Philadelphia, PA USA; 4 Institute of Pharmacy, Martin-Luther University Halle-Wittenberg, Halle (Saale), Germany

**Keywords:** ACE2, Aurora A kinase, SARS-CoV-2, spike/ACE2 interactions

## Abstract

Isatin (indol-2,3-dione), a secondary metabolite of tryptophan, has been used as the core structure to design several compounds that have been tested and identified as potent inhibitors of apoptosis, potential antitumor agents, anticonvulsants, and antiviral agents. In this work, several analogs of isatin hybrids have been synthesized and characterized, and their activities were established as inhibitors of both Aurora A kinase and severe acute respiratory syndrome coronavirus 2 (SARS-CoV-2) spike/host angiotensin-converting enzyme II (ACE2) interactions. Amongst the synthesized isatin hybrids, compounds **6a**, **6f**, **6g**, and **6m** exhibited Aurora A kinase inhibitory activities (with IC_50_ values < 5 $\mu$M), with GScore values of −7.9, −7.6, −8.2 and −7.7 kcal/mol, respectively. Compounds **6g** and **6i** showed activities in blocking SARS-CoV-2 spike/ACE2 binding (with IC_50_ values in the range < 30 $\mu$M), with GScore values of −6.4 and −6.6 kcal/mol, respectively. Compounds **6f**, **6g**, and **6i** were both capable of inhibiting spike/ACE2 binding and blocking Aurora A kinase. Pharmacophore profiling indicated that compound **6g** tightly fits Aurora A kinase and SARS-CoV-2 pharmacophores, while **6d** fits SARS-CoV-2 and **6l** fits Aurora A kinase pharmacophore. This work is a proof of concept that some existing cancer drugs may possess antiviral properties. Molecular modeling showed that the active compound for each protein adopted different binding modes, hence interacting with a different set of amino acid residues in the binding site. The weaker activities against spike/ACE2 could be explained by the small sizes of the ligands that fail to address the important interactions for binding to the ACE2 receptor site.

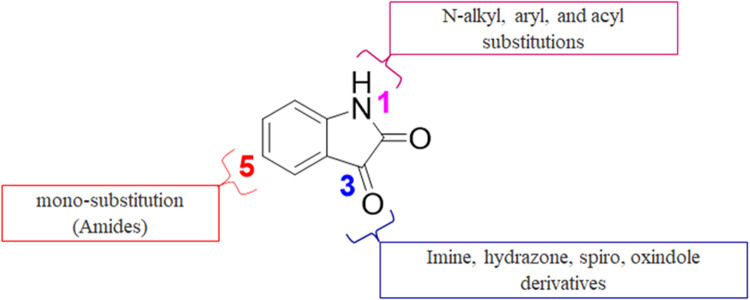

## Introduction

Efficiencies in synthesis through modern synthetic chemistry in confluence with the development of automation and various combinatorial techniques have enabled the drug industry to build substantial screening collections [1]. To mitigate the investment of resources and to manage the universe of the nearly infinite potential of small molecules there has been a focus on scaffold classes that contain lead-like properties, follow rules outlined by Linpiski et al. [2] and Veber et al. [3] or are limited to compounds that are easy to synthesize.

Cancer is a life-threatening disease that kills millions of people each year [4, 5] and despite the best efforts, it continues to resist full control and eradication [6, 7]. Interestingly, the overexpression of Aurora kinases and their association with genetic instability and aneuploidy in tumors suggests that a wide range of cancers could respond therapeutically to inhibitors of the Aurora kinases [8, 9]. Aurora A kinase, a multifunctional protein that is highly implicated in cancer, is very important in the regulation of mitotic progression. It is well known that if this protein is interrupted with the targeted therapy, mitotic progression gets interrupted, which leads to the death of malignant cells [10–12]. During the outbreak of COVID-19 in late 2019, there was widespread interest in the repurposing of most anticancer drugs as potential inhibitors of the severe acute respiratory syndrome coronavirus 2 (SARS-CoV-2) spike and host angiotensin-converting enzyme 2 (ACE2) interactions as a strategy to prevent viral transmission [13, 14].

SARS-CoV-2 is the causative agent of the pandemic viral disease COVID-19 [15, 16]. Although the speed with which COVID-19 vaccines have been developed is remarkable, their long-term protection effect and effectiveness against emerging variants and potential future variants of SARS-CoV-2 and other coronaviruses remains to be determined [17–20]. Considering that ACE2 also serves as the receptor for the SARS-CoV and SARS-CoV-2 viruses [13, 14], the binding of the S1 domain of the SARS Coronavirus spike protein to ACE2 initiates viral entry into the host cell [21–23]. This area of interaction between SARS-CoV-2 and ACE2 has become of therapeutic interest to develop possible treatments against COVID-19 [24, 25].

Isatin (indol-2,3-dione), Fig. S1 (Supplementary Data), a secondary metabolite of tryptophan is widely distributed in the central nervous system, mammalian tissues, and body fluids of humans [26, 27]. This oxidized indole has been used as the core structure in the designation of several compounds that have been tested and identified as potent inhibitors of apoptosis [28–30], potential antitumor agents [28, 29], anticonvulsants [31, 32] as well as antiviral agents [30, 33]. Isatin, therefore, is considered a versatile and favorable precursor for pharmacophore development among the privileged scaffolds because the moiety can be modified at various positions (N-1, C‐3, C‐4, C‐5, and C‐7), as illustrated in Fig. S1 (Supplementary Data), resulting in different derivatives with diverse biological properties [34].

**Fig. 1:**
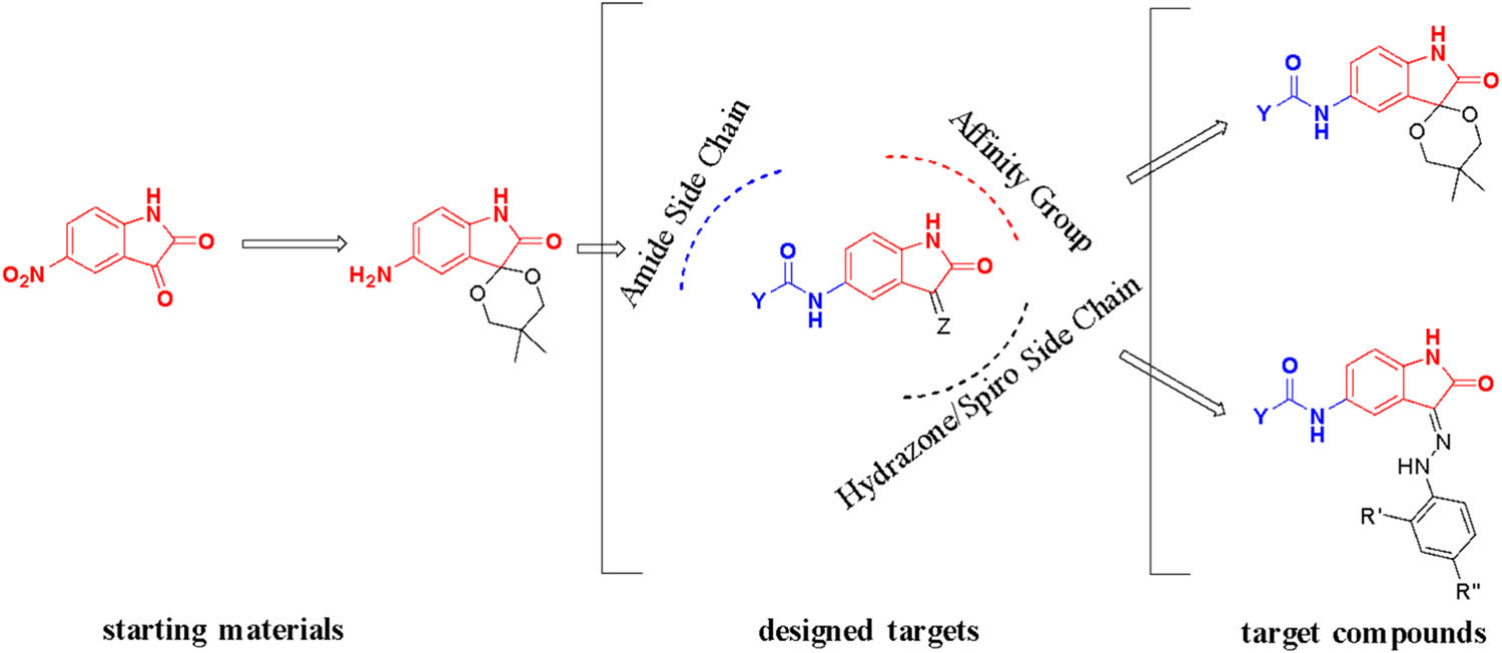
Design of the target compounds

The purpose of this work was to lay the groundword for antiviral discovery and establish an independent and African-led drug discovery and development research center at the University of Buea to research and treat diseases that disproportionately affect Africans. Toward this end, we are building an open-access pan-African library of natural and synthetic compounds. We had identified the isatin scaffold as one with potential biological activities and we sought to synthesize a pseudo-natural product library (Fig. 1) and evaluate these new compounds for biological activities against anticancer and antiviral drug targets. This work is meant to affirm the activities of isatin analogs as inhibitors of both Aurora A kinase, and SARS-CoV-2 spike and host ACE2 interactions and to establish their mechanisms of action as well as their structure-activity relationships.

## Results and discussion

### Chemistry

**Scheme 1:**
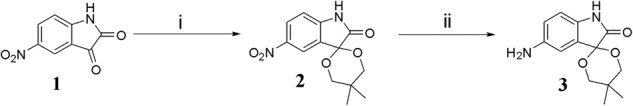
Formation of intermediate 3. (i) neopentyl glycol, *p*-toluenesulfonic acid, *n*-heptane, 125 °C, 36 h in N_2_ atm using Dean stark apparatus; (ii) Pd/C, H_2_, ethyl acetate, r.t., 3 h

**Scheme 2:**
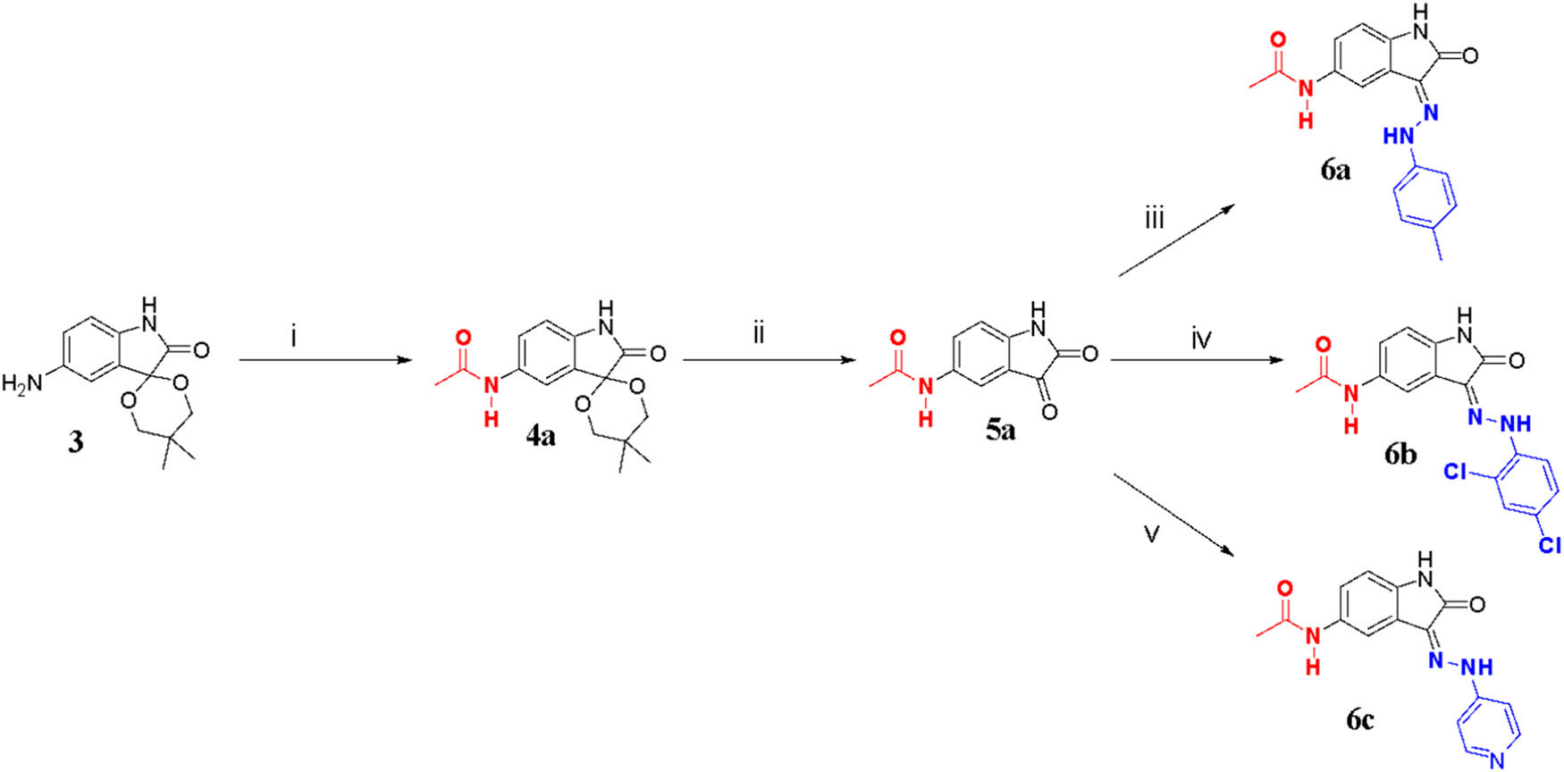
Synthesis of compounds **6a** – **6c**. (i) acetyl chloride, K_2_CO_3_, ethyl acetate, 0 °C, 12 h; (ii) glacial acetic acid, concentrated hydrochloric acid, r.t., 2 h; (iii) *p*-tolyhydrazine hydrochloric acid, EtOH, acetic acid, 80–85 °C, 2 h; (iv) 2,4 dichlorohydrazine hydrochloric acid, EtOH, acetic acid, 80–85 °C, 2 h. (v) 4-hydrazinylpyridine, EtOH, acetic acid, 80–85 °C, 2 h

**Scheme 3:**
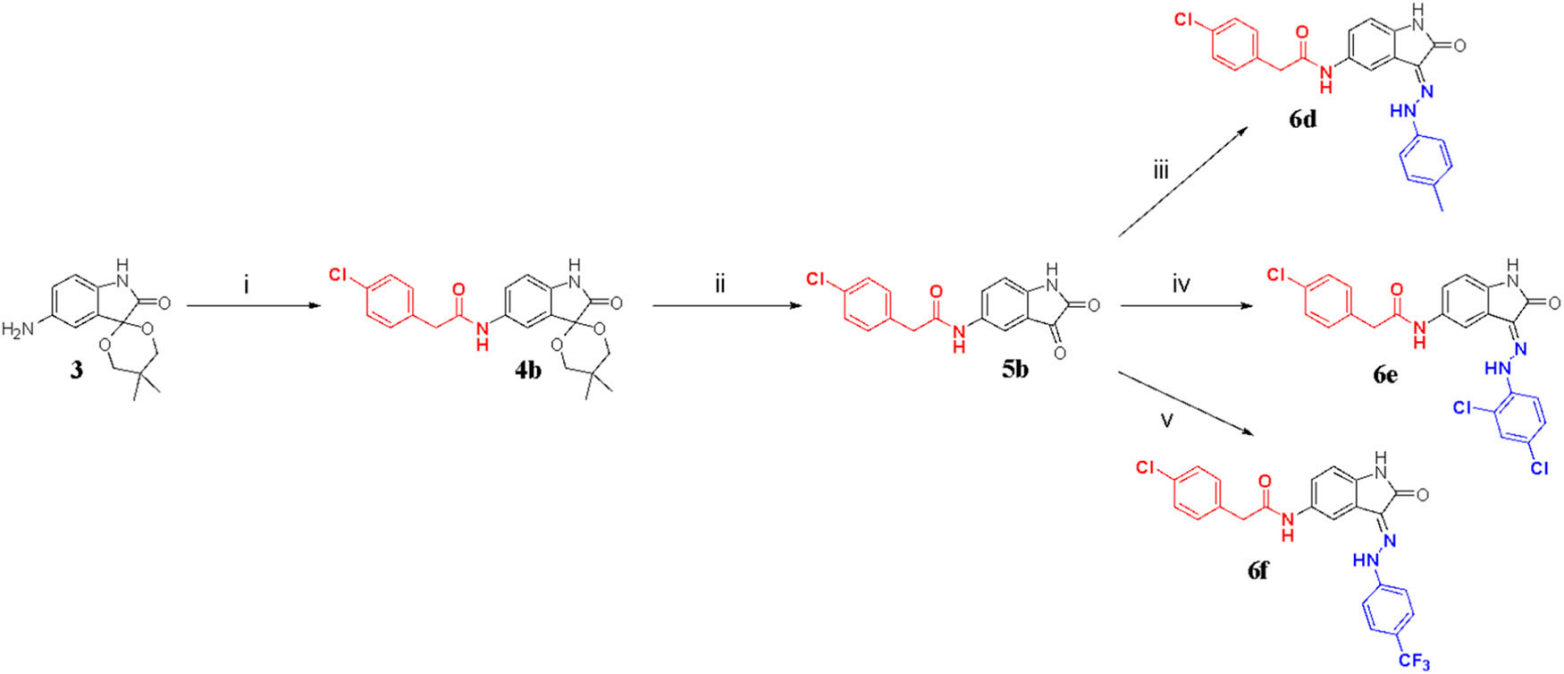
Synthesis of compounds **6d** – **6** **f**. (i) 2-(4-cholorophenyl)acetic acid, HATU, DIPEA, Dimethylformamide (DMF), N_2_ atm, r.t., 1 h (ii) glacial acetic acid, concentrated hydrochloric acid, r.t., 2 h (iii) *p*-tolyhydrazine hydrochloric acid, EtOH, acetic acid, 80–85 °C, 2 h; (iv) 2,4 dichlorohydrazine hydrochloric acid, EtOH, acetic acid, 80–85 °C, 2 h; (v) (4-(trifluoromethyl)phenyl)hydrazine, EtOH, acetic acid, 80–85 °C, 2 h

**Scheme 4:**
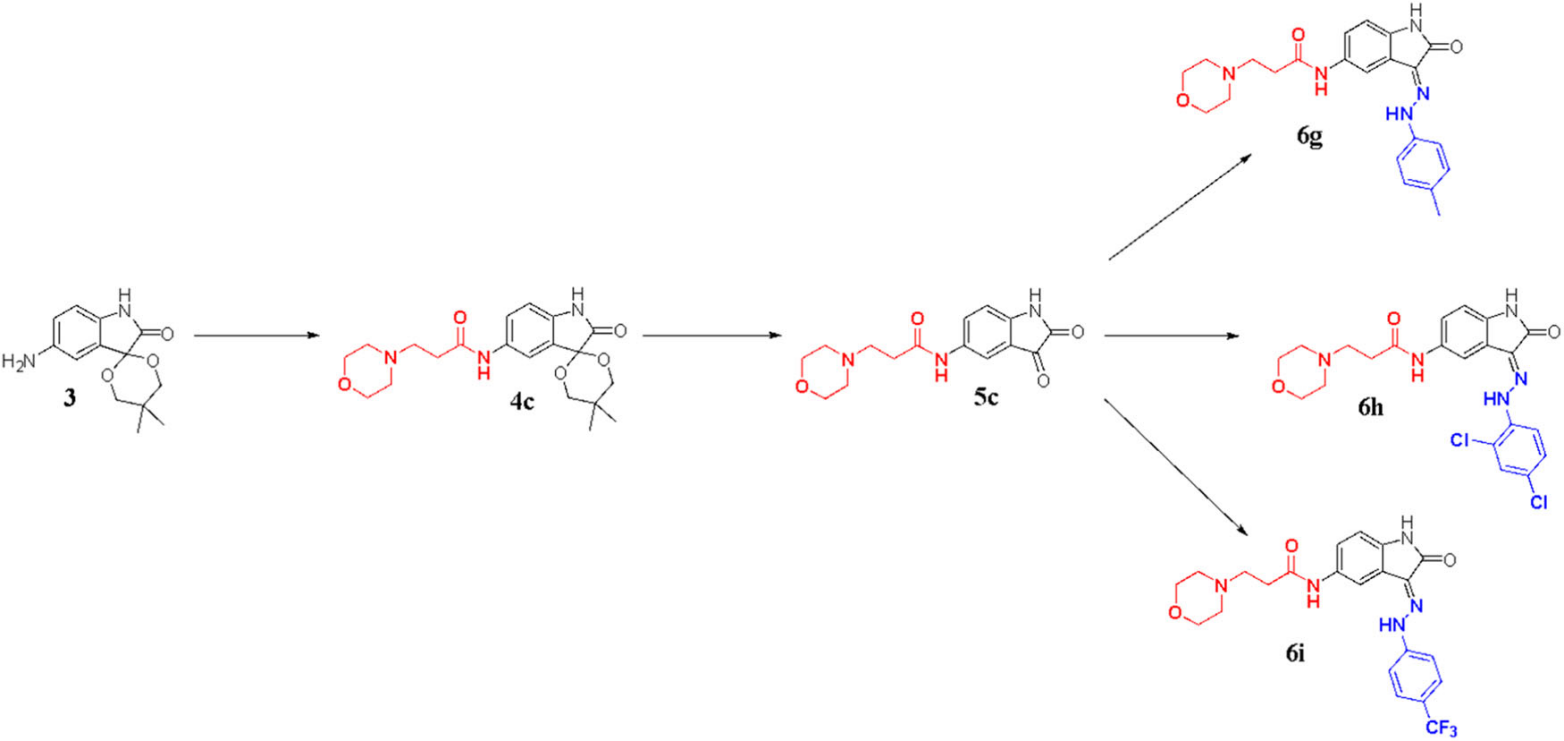
Synthesis of compounds **6** **g** – **6i**. (i) 3-morpholinopropanoic acid, HATU, DIPEA, DMF, N_2_ atm, r.t., 1 h (ii) glacial acetic acid, concentrated hydrochloric acid, r.t., 2 h (iii) *p*-tolyhydrazine hydrochloric acid, EtOH, acetic acid, 80–85 °C, 2 h; (iv) 2,4 dichlorohydrazine hydrochloric acid, EtOH, acetic acid, 80–85 °C, 2 h; (v) (4-(trifluoromethyl)phenyl)hydrazine, EtOH, acetic acid, 80–85 °C, 2 h

**Scheme 5:**
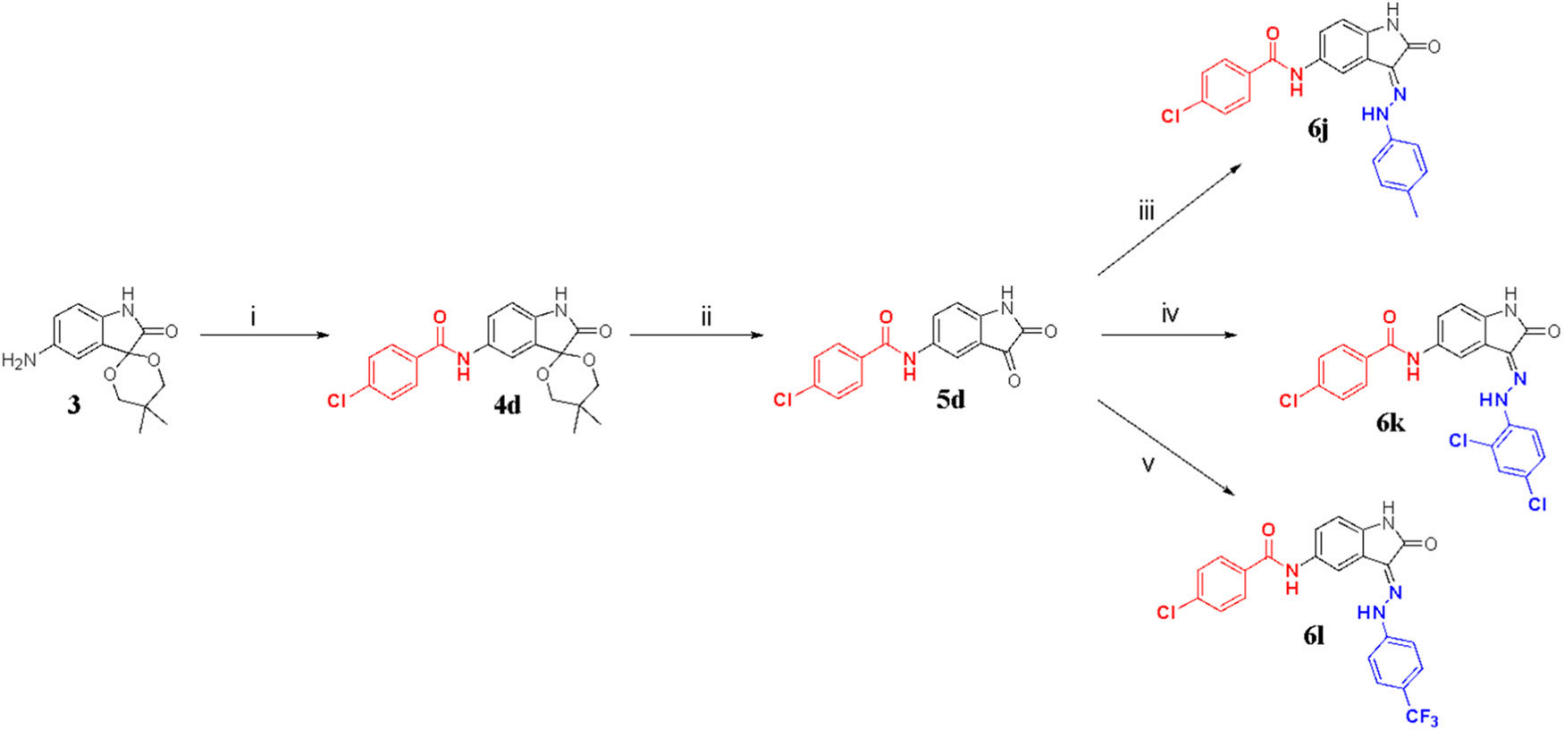
Synthesis of compounds **6j** – **6** **l**. (i) 4-chlorobenzoic acid, HATU, DIPEA, DMF, N_2_ atm, r.t., 1 h (ii) glacial acetic acid, concentrated hydrochloric acid, r.t., 2 h (iii) *p*-tolyhydrazine hydrochloric acid, EtOH, acetic acid, 80–85 °C, 2 h; (iv) 2,4 dichlorohydrazine hydrochloric acid, EtOH, acetic acid, 80–85 °C, 2 h; (v) (4-(trifluoromethyl)phenyl)hydrazine, EtOH, acetic acid, 80–85 °C, 2 h. Synthesis of compound **6** **m**. (i) 4-chlorobenzoic acid, HATU, DIPEA, DMF, N_2_ atm, r.t., 1 h (ii) glacial acetic acid, concentrated hydrochloric acid, r.t., 2 h (iii) 4-hydrazinylpyridine, EtOH, acetic acid, 80–85 °C, 2 h

The syntheses of the 2,3-indolinedione derivatives are depicted in Schemes 1–5 and S1 (Supplementary Data). Commercially available 5-nitroisatin **1** was used to prepare the intermediate (5′,5′-dimethyl-5-nitrospiro[indoline-3.2’[1,3]dioxan]-2-one) **2**. Intermediate **2** was prepared by treating **1** with neopentyl glycol, with catalysis by *p*-toluenesulfonic acid illustrated in Scheme 1. With Pd/C (10%, w/w) as the catalyst, the nitro group in intermediate **2** was converted to an amino group by hydrogenation to prepare intermediate **3**.

Intermediate **3** was allowed to react with acetyl chloride in the presence of anhydrous potassium carbonate to achieve the amide-spiro compound **4a**. Compound **4a** under acidic conditions was permitted to undergo deprotection to obtain intermediate **5a**. This was followed by Schiff base reaction with arylhydrazines to achieve the expected target compounds **6a** – **6c** as indicated in Scheme 2.

Intermediate **3** was reacted with 2-(4-cholorophenyl)acetic acid in the presence of amide coupling agent Hexafluorophosphate Azabenzotriazole Tetramethyl Uronium/*N*,*N*-Diisopropylethylamine (HATU/DIPEA) to obtain amide-spiro arylated compound **4b**. Compound **4b** was then deprotected under acidic conditions to obtain the intermediate **5b**. This was followed by Schiff base reaction to achieve the expected target compounds **6d** – **6** **f** shown in Scheme 3.

Intermediate **3** was also treated with 3-morpholinopropanoic acid in the presence of amide coupling (HATU/DIPEA) to obtain amide-spiro arylated compound **4c** followed by deprotection reaction under acidic conditions to obtain the intermediate **5c**. The intermediate **5c** was allowed to undergo Schiff base reaction to achieve the expected compounds **6** **g** – **6i** as illustrated in Scheme 4.

Intermediate **3** was also reacted with 4-chlorobenzoic acid in the presence of amide coupling (HATU/DIPEA) to obtain arylated compound **4d**. Compound **4d** was then treated under acidic conditions to allow it to undergo deprotection to obtain the intermediate **5d** followed by a Schiff base reaction with arylhydrazines to achieve the final target compounds **6j** – **6** **m** as shown in Scheme 5 and S1 (Supplementary Data).

### Biochemical screening

The Aurora A kinase inhibitory activities and blockage of fusion of the SARS-CoV-2 viral spike with the human ACE2 of the synthesized compounds are shown on Table 1.

**Table 1:** Summary of the ability of the compounds to inhibit aurora A kinase and SARS-CoV-2 spike/ACE2 fusion

Compound	Aurora A Kinase			Spike RBD/ACE2		
	Percent block at 32 $\mu$M	IC_50_ ($\mu$M)	GScore (kcal/mol)	Percent block at 32 $\mu$M	IC_50_ ($\mu$M)	GScore (kcal/mol)
**4a**	9.1 ± 6.5	n.d.	−7.0	0 ± 0	n.d.	−5.8
**4b**	20.9 ± 6.3	n.d.	−6.3	20.1 ± 11.5	n.d.	−5.9
**4c**	1.6 ± 2.1	n.d.	−7.1	2.0 ± 2.9	n.d.	−6.0
**4d**	17.8 ± 9.2	n.d.	−6.8	22.5 ± 5.7	n.d.	−5.4
**4e**	11.0 ± 9.5	n.d.	−7.0	0.8 ± 1.2	n.d.	−5.9
**6a**	82.7 ± 2.7	0.8 ± 0.1	−7.9	21.5 ± 4.8	n.d.	−6.1
**6b**	26.4 ± 0.6	n.d.	−8.3	21.5 ± 6.2	n.d.	−5.8
**6c**	58.1 ± 1.7	5.5 ± 0.5	−9.2	5.2 ± 4.1	n.d.	−6.3
**6d**	54.2 ± 6.7	22.8 ± 7.9	−7.3	3.2 ± 0.4	n.d.	−6.4
**6e**	10.0 ± 4.9	n.d.	−7.7	10.4 ± 12.1	n.d.	−6.2
**6** **f**	87.8 ± 2.9	1.8 ± 0.2	−7.6	27.1 ± 3.9	n.d.	−6.5
**6** **g**	79.2 ± 4.9	3.1 ± 0.7	−8.2	56.8 ± 5.2	22.4 ± 0.8	−6.4
**6** **h**	10.3 ± 7.1	n.d.	−8.1	21.3 ± 5.6	n.d.	−6.5
**6i**	79.3 ± 1.5	4.8 ± 0.3	−8.3	64.5 ± 9.0	12.1 ± 2.9	−6.6
**6j**	13.2 ± 8.2	n.d.	−7.2	2.8 ± 4.0	n.d.	−5.6
**6k**	13.4 ± 14.6	n.d.	−7.3	34.4 ± 20.4	n.d.	−5.3
**6** **l**	19.4 ± 13.5	n.d.	−7.0	44.2 ± 9.0	n.d.	−5.6
**6** **m**	80.2 ± 1.2	3.1 ± 1.3	−7.7	0 ± 0	n.d.	−5.7
Hesperadin	93.8 ± 0.8	0.0053 ± 0.0027	−8.0	43.3 ± 10.6	n.d.	−6.7
Hopeaphenol	n.d.	n.d.	−7.5	83.6 ± 2.0	0.3 ± 0.1	−9.6

**Fig. 2:**
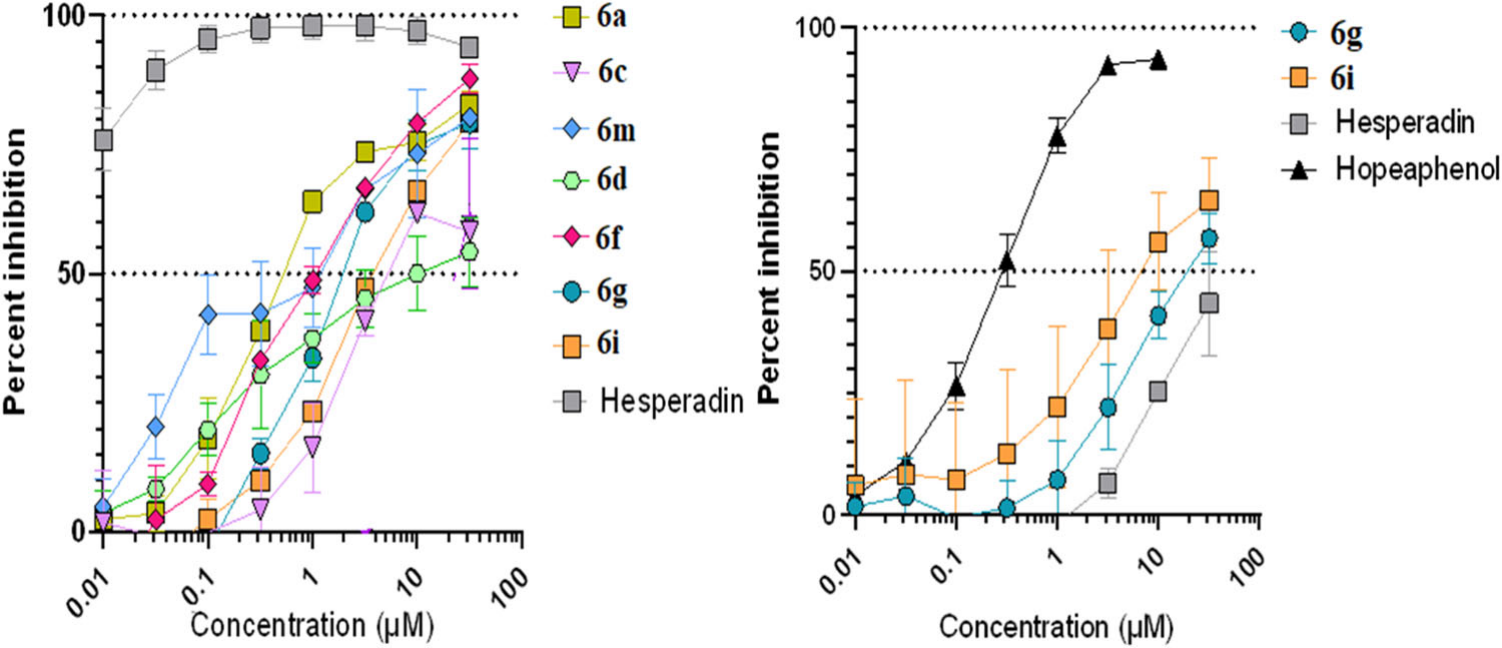
Dose-response curves for (left) Aurora A kinase inhibition of the compounds and (right) Spike RBD/ACE2 inhibition of the compounds

The results show the activities of the compounds on the ability to inhibit aurora A kinase using the established homogeneous time-resolved fluorescence (HTRF) kinase assay [35], which can measure Aurora kinase 1 activity by its autophosphorylation. The results obtained using this assay were able to show that the control compound hesperadin (Fig. 2), could inhibit Aurora kinase with an IC_50_ value of 5.3 nM, consistent with the standard value [36]. Several of the compounds (Table 1) were also able to inhibit Aurora kinase, with three compounds of series 6 (**6a,** **6m**, and **6f**) having the best activities (IC_50s_ = 0.8, 1.8, and 3.1 $\mu$M, respectively).

The compounds were also tested to disrupt the interaction of the SARS-CoV-2 spike protein (original Wuhan variant) with its host ACE2 receptor using an Alpha-screen method. Here, two recombinant proteins; the spike receptor domain and the ACE2 protein were used, and each fused to a donor and acceptor bead. When binding between the proteins occurs, a singlet oxygen transfer occurs between the beads which promotes luminescence. Compounds that can inhibit this interaction will also inhibit luminescence. The control inhibitor hopeaphenol could block this interaction with an IC_50_ of 0.3 $\mu$M, consistent with its published values [37]. Two of the compounds, **6g** and **6i**, showed respective IC_50_ values of 12.5 and 22.4 $\mu$M. Generally, both assays indicate that Aurora kinase inhibitors have additional leads from the potential anti-SARS-CoV-2 entry inhibitors (Fig. 2).

### Computer modeling

An explanation of the structure-activity relationships was arrived at through computer modeling of selected compounds from the series against their respective drug targets.

#### In silico analysis of series 4 and 6 ligands binding to Aurora A kinase ligand-receptor pair

**Fig. 3:**
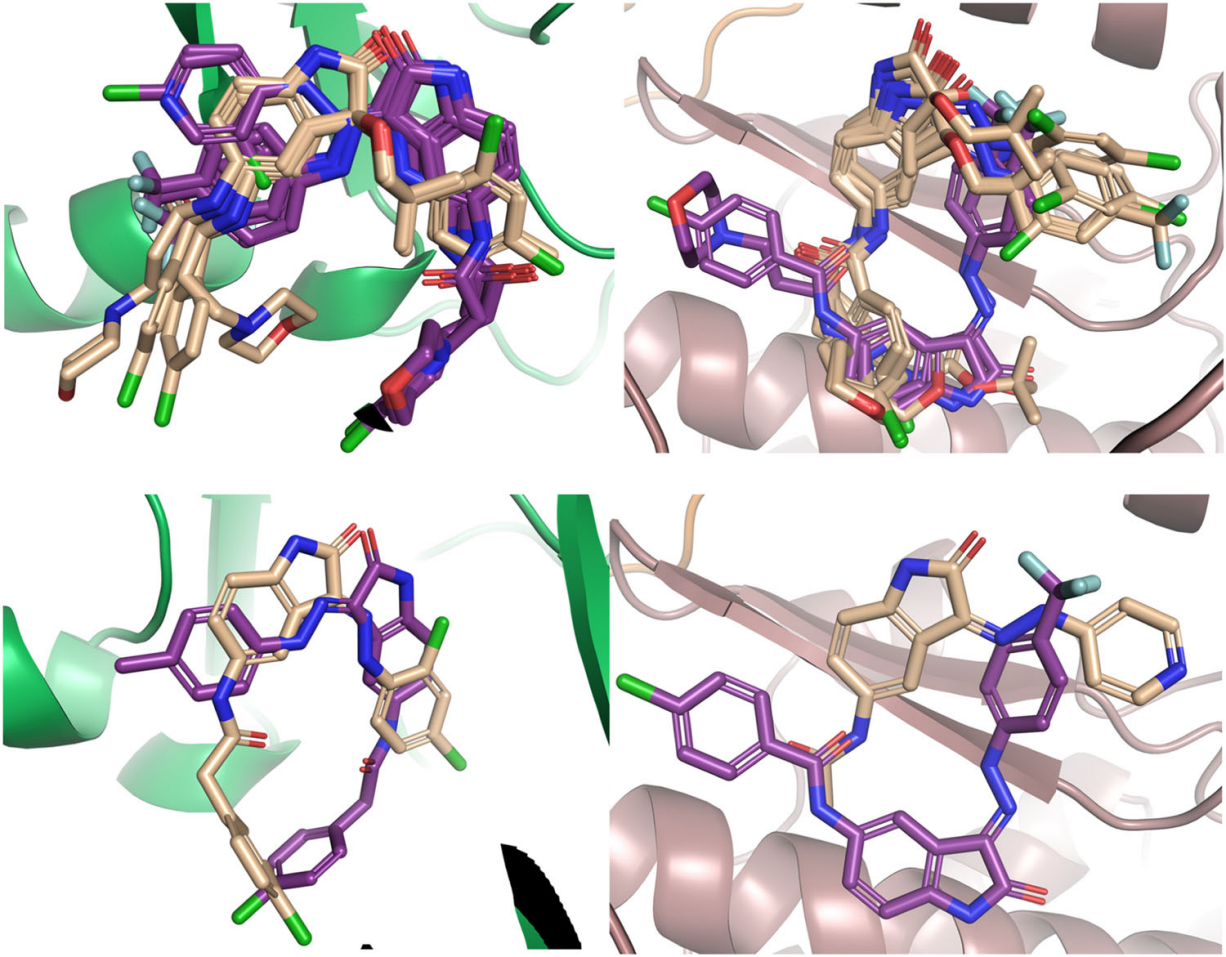
Superposition of the docked ligands at the docking sites (top left) All docked compounds in Aurora A kinase in green (top right) All docked compounds in the spike/ACE2 in brown (B) receptor site. Active compounds in deep purple and inactive compounds in light orange; (bottom left) the most active (**6l**) and least active (**6c**) ligands in the docked sites for the Aurora A kinase and (bottom right) spike/ACE2 receptor site. The active compounds are in deep purple and the inactive in light orange

To perform the docking, we first established a protocol by docking validation which consisted in re-docking the native or co-crystallized ligand from each protein target. This was necessary to ensure that the docking procedure could reproduce the binding mode of the inhibitor, imidazo[4,5-b]pyridine, co-crystallized with Aurora A kinase in the X-ray structure (PDB code: 4BYI). This docking validation gave a root mean square deviation (RSMD) of 0.5 Å hence, encouraging the adoption of the established docking protocol. Additionally, the small sizes of both active and inactive synthesized compounds showed that they could putatively bind at the same area as the co-crystallized ligand, which is an inhibition of Aurora A kinase at the protein receptor binding domain (RBD), see Fig. 3.

**Fig. 4:**
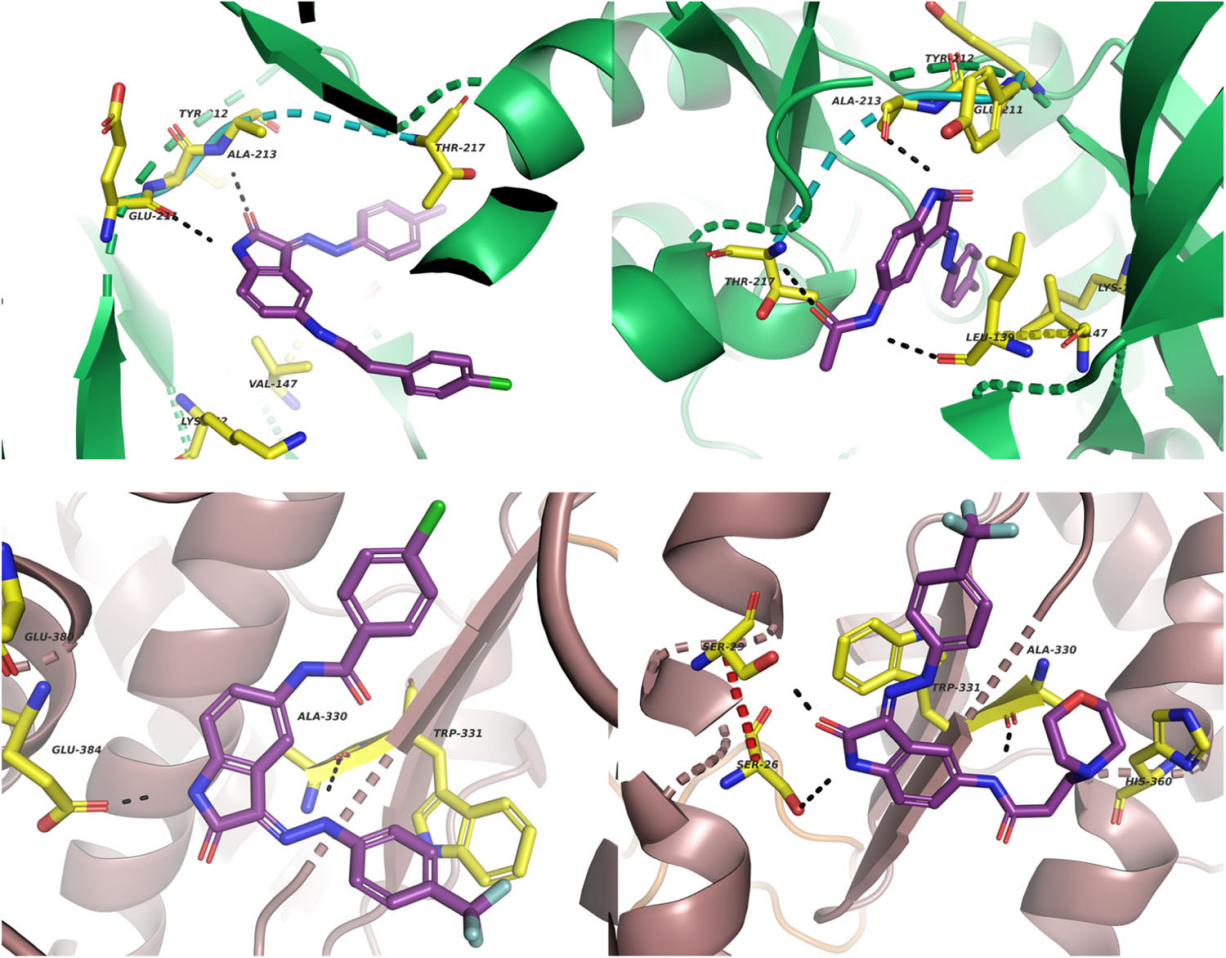
Interaction of the most active of the ligands at the Aurora A kinase (in green) binding site (top left) **6d** and (top right) **6a**. Active ligand in deep purple, residues at the binding site in yellow and interactions in black dotted lines. (bottom left) Interaction of the most active ligands at the Spike/ACE2 receptor site (in brown) with **6** **l** on the left, and (bottom right) **6i**. Active ligand in deep purple, residues at the binding site in yellow and interactions in black dotted lines

Additionally, a superposition of the docking active and inactive compounds for both Aurora A kinase and spike/ACE2 showed that the active and inactive ligands bind differently, overall (Fig. 3). Looking closely at the different interactions, as was exemplified for the active compounds **6a** and **6d** for Aurora A kinase (Fig. 4), it was shown that they both interact with Leu139 and Ala213. It has been proven that the Aurora A kinase domain consists of a hydrophobic pocket composed of Leu263, Gly216, and Leu139 [38]. Besides this, the amino acid duo Ala213 and Arg137 is known to assist in anchoring the ligand to direct them in the abovementioned hydrophobic pocket through bidentate hydrogen bond formation. It was also shown that small molecules with the hydrophobic feature toward the hydrophobic pocket of the receptor are crucial for Aurora-A kinase inhibitory activity. This scenario was also seen in almost all the active ligands and, therefore, these interactions helped to stabilize the active ligands in their binding to the RBD, thus orientating the ligands with hydrophobic features towards the hydrophobic pocket of the receptor. This has been previously reported to be crucial for Aurora A kinase inhibitory activity [38–40]. For Leu139, this residue interacts with the amide functional group of the most active ligand 6a, while Ala213 is seen to interact with the central isatin core of all the active Aurora A kinase inhibitors (Fig. 4). The former amide functionality also interacts favorably with the Thr227 sidechain, thus making it an important component of the bioactive pharmacophore for Aurora A kinase inhibitors in this series.

In addition to Leu139 and Ala213 residues, it was observed that the docked pose of the ligand **6d**, which expressed the desired in vitro activity, also interacted with Glu211. Meanwhile, it was observed that active compounds **6a,** **6b,** **6c,** **6f,** **6g**, and **6i** all make interactions with both Lys162 and Glu211, indicating that Lys162 and Glu211 could be important for activity. It must be noted that, since Lys is hydrophilic, interactions with the hydrophobic portions of the ligands with hydrophobic residues would bring about increased activity [38–41]. Most importantly, in this work, ligand **6a** was seen to have an interaction with Thr217 which, from previous studies, has been proven to account for selectivity towards Aurora A kinase. This implies that **6a** is an active and selective compound [42].

#### In silico analysis of series 4 and 6 ligands binding to RBD-ACE2 ligand-receptor pair

From the computational studies performed on the wild-type (WT) or the Wuhan variant of SARS-CoV-2. The docking studies showed that ligands preferably bind at the ACE2 receptor binding site. This correlates with some previous studies revealing ligands bind at the ACE2 binding site and induce conformational changes that influence the interaction of the spike/ACE2 receptor fusion, hence preventing recognition of the RBD of the viral spike by the host protein [43–45]. Only two of the tested compounds (**6g** and **6i**) showed activities against spike/ACE2 fusion and even showed weaker activities when compared to their inhibitory concentration against Aurora A kinase. This could be explained by the relatively small size and weaker hydrophobicities of these ligands when compared to those of much bulkier ligands that are known to bulk the spike/ACE2 fusion, e.g. the natural products hepeaphenol, vaticanol B and vatalbinoside A [37]. The docked poses of these compounds showed that a significant part of the binding site remained unoccupied and many of the key residues for interacting with the angiotensin II receptor site were not addressed (Fig. 4).

## Conclusion

There has been a quest to repurpose anticancer compounds as potential treatment options for COVID-19. In this work, we report the synthesis, full characterization, and screening of 18 novel oxindole derivatives for the inhibitory potential against Aurora A kinase and SARS-CoV-2 spike/ACE2 fusion from the isatin scaffold. The results showed that seven of the synthesized compounds had 50% inhibitory concentrations (IC_50_) against Aurora A kinase at less than 25 micromolar, while two of the compounds had IC_50_ values of less than 25 micromolar. Interestingly, these two compounds (**6g** and **6i**) could also be dual inhibitors of both protein targets, with respective docking scores (GScore values) of −6.4 and −6.6 kcal/mol, respectively against spike/ACE. The docking scores were −8.2 and −8.3 kcal/mol, respectively, against Aurora A kinase. Although the docking scores could not clearly distinguish between the active and inactive compounds or help to establish a clear SAR, the range of docking scores for docking each protein were quite different, i.e. −6.3 kcal/mol ≤ GScore ≤ −9.2 kcal/mol for Aurora A kinase and −5.3 kcal/mol ≤ GScore ≤ −6.6 kcal/mol for spike/ACE2. Apart from GScore = 6.3 kcal/mol or above, six of the docked compounds showed higher docking scores against spike/ACE2 than against Aurora A kinase. In general, the compounds had lower (better) docking scores as well as better biological activities for the Aurora A kinase than against spike/ACE2. This implies these compounds contain pharmacophores required for dual inhibition and are to be fine-tuned to improve dual potency. Besides, the size and hydrophobic properties of the angiotensin II receptor site in the spike/ACE2 complex necessitate a bulkier ligand to address all required amino acid residues for inhibition of spike/ACE2 fusion. Dimerization of these compounds is under consideration as an option to create bulkier ligands as next-generation scaffolds for the angiotensin II receptor site with an isatin core. Molecular modeling has provided further insights, particularly that the active and inactive compounds adopt different binding modes in the respective binding sites, clearly showing different interaction patterns with the binding site amino acid residues. Recent studies have shown that targeted therapy against different cancers could be a therapeutic option against cancer-specific cells and toward the signaling pathways is a valuable avenue of research [46] The kinase family of anticancer drug targets is also known to provide a valuable source of biological targets against both cancers and COVID infections [47]. As an example, kinases like the tyrosine kinases, Rho kinase, Bruton tyrosine kinase, ABL kinases, and NAK kinases play an important role in the modulation of signaling pathways involved in both cancers and viral infections such as COVID [48]. For the Aurora A kinase inhibitors, it was shown that the important residues for binding were Leu139, Ala213, Lys162, and Glu211. It was also shown that small molecules with the hydrophobic feature toward the hydrophobic pocket of the receptor are crucial for Aurora A kinase inhibitory activity. For the spike/ACE2 inhibition, although the biological screening only gave the most active compounds (**6g** and **6i**) with IC_50_ values 12.1 and 22.4, respectively, these show the starting positions for further structural modifications for the generation of more active analogs with much lower IC_50_ values. This work is laying the foundation for the discovery of dual inhibitors of SARS-CoV-2 spike/ACE2 fusion and Aurora A kinase, which could eventually be developed into anticancer agents with potential for COVID-19 treatment.

## Experimental section

### Chemistry

All the chemical reagents and solvents were purchased from commercial sources and were used without further purification. Reactions were monitored using thin-layer chromatography (TLC) performed on SGF254 plates. Chromatographic separations were performed using column chromatography on silica gel (60 Å, 200–300 mesh). Melting points were determined on a Büchi capillary melting point apparatus (Buchi Labortechnik AG, University of Buea) without correction. The ^1^H NMR and ^13^C NMR spectra were recorded at 400 MHz, on a Bruker Avance DRX-400 Spectrometer (Bruker, USA) in deuterated dimethyl sulfoxide (DMSO-d_6_) with tetramethylsilane (TMS) as the internal standard. Peak multiplicities were expressed as follows: singlet (s), doublet (d), triplet (t), quartet (q), multiplet (m), broad singlet (br s), doublet of doublets (dd), doublet of triplets (dt), and quartet of doublets (qd). The mass spectra (MS) were measured with LCQ FLEET (ThermoFisher, USA). The purity of the compounds was determined by HPLC performed on a Shimadzu LC-20ATVP Liquid Chromatograph equipped with an SPDM20A UV VIS Detector using a C18 column (size: 250 mm × 4.6 mm). Elution solvent: 75% methanol and 25% water. The elution rate was 1.00 mL/min, and the injection volumes were 10 μL at 25 °C and detection at 253 nm. All spectral data (^1^H, ^13^C and LC-MS have been included in the Supplementary Data).

#### Synthesis of 5′,5′-dimethyl-5-nitrospiro[indoline-3,2′-[1,3]dioxan]- 2-one (**2**)

5-nitroindoline-2,3-dione (5 g, 26.02 mmol), neopentyl glycol (8.13 g, 78.07 mmol) and *p*-toluene sulfonic acid (447 mg, 2.60 mmol) under an inert atmosphere of nitrogen were successively added and dissolved in *n*-heptane (200 mL). The resulting reaction mixture was refluxed for 36 h at 125 °C under reflux using a Dean-Stark apparatus. After completion of the reaction, the mixture was cooled to room temperature. The solid collected was dissolved in 200 mL ethyl acetate and was washed with water (2 × 100 mL) and brine solution (1 × 100 mL). Then, the organic layer was dried over anhydrous sodium sulfate and the solution was filtered under reduced pressure. Purification by chromatography on silica gel (70% ethyl acetate/hexane). Cream white solid; yield 90%; ^1^H NMR (400 MHz, DMSO-d6) δ ppm 11.21 (s, 1H), 8.29 (dd, *J* = 8.7, 2.5 Hz, 1H), 8.08 (d, *J* = 2.4 Hz, 1H), 7.04 (d, *J* = 8.7 Hz, 1H), 4.49 (d, *J* = 10.9 Hz, 2H), 3.55 (d, *J* = 10.9 Hz, 2H), 1.34 (s, 3H), 0.84 (s, 3H). MS(ESI): cald for C_13_H_14_N_2_O_5_ [M+H]^+^ 278.26, found 278.18; LC(ESI): t_R_ 2.42 min, purity 90%.

#### Synthesis of 5-amino-5’,5’-dimethylspiro[indoline-3,2’-[1,3] dioxan]-2-one (**3**)

To a solution of intermediate **2** (8 g, 28.75 mmol) in methanol (200 mL) was added 10% Pd/C (68.10 mg). The mixture was subjected to hydrogen for 3 h at room temperature. The reaction mixture was filtered, and the filtrate was concentrated under reduced pressure. The crude material was recrystallized with ethyl acetate/hexane to obtain intermediate (**3**). Purification by chromatography on silica gel (50% ethyl acetate/hexane). Brown solid; yield 81%; ^1^H NMR (400 MHz, DMSO-d6) δ ppm 9.97 (s, 1H), 6.68 (s, 1H), 6.47 (s, 2H), 4.82 (s, 2H), 4.49 (d, *J* = 10.8 Hz, 2H), 3.43 (d, *J* = 10.9 Hz, 2H), 1.27 (s, 3H), 0.81 (s, 3H). MS(ESI): cald for C_13_H_16_N_2_O_3_ [M+H]^+^ 248.28, found 248.00; LC(ESI): t_R_ 1.00 min, purity 95%.

#### Synthesis of *N*-(5’,5’-dimethyl-2-oxospiro[indoline-3,2’-[1,3]dioxan]-5-yl)acetamide (**4a**)

To a solution of intermediate **3** (700 mg, 2.80 mmol) and anhydrous K_2_CO_3_ (584.48 mg, 4.23 mmol) in ethyl acetate (50 mL) was added acetyl chloride (265.57 mg, 3.38 mmol) at 0°C. The resulting solution was stirred for 12 h at room temperature. After completion of the reaction, the mixture was filtered. The organic phase was successively washed with water (15 mL × 3) and saturated brine (15 mL × 3) and then evaporated under reduced pressure to obtain compound **4a**, which was directly used for the next step without further purification. Cream white solid; yield 90%; ^1^H NMR (400 MHz, DMSO-d6) δ ppm 10.35 (s, 1H), 9.87 (s, 1H), 7.67 (d, *J* = 2.2 Hz, 1H), 7.45 (dd, *J* = 8.4, 2.2 Hz, 1H), 6.73 (d, *J* = 8.4 Hz, 1H), 4.50 (d, *J* = 10.8 Hz, 2H), 3.48 (d, *J* = 10.9 Hz, 3H), 2.02 (s, 1H), 2.01 (s, 1H), 1.29 (s, 3H), 0.83 (s, 3H). MS(ESI): cald for C_15_H_18_N_2_O_4_ [M+H]^+^ 290.32, found 290.08; LC(ESI): t_R_ 1.76 min, purity 98%.

#### Synthesis of amide-spiro compounds (**4b – 4d**)

Intermediate **3** (500 mg, 2.01 mmol), aryl carboxylic acids (2.42 mmol), and HATU (1.15 g, 3.02 mmol) in dimethyl fluoride (3 mL) solution under an inert atmosphere of nitrogen was added dropwise DIPEA (780.85 mg, 6.04 mmol) and the resulting solution was stirred for 1 h at room temperature. The residue was treated with a saturated solution of sodium carbonate NaHCO_3_ and extracted with ethyl acetate. The organic layer was dried (Na_2_SO_4_) and the filtrate was concentrated under reduced pressure. The crude residue was purified by chromatography on silica gel, with a corresponding eluent system to give the expected compounds (**4b – 4d**).

##### 2-(4-chlorophenyl)-N-(5’,5’-dimethyl-2-oxospiro[indoline-3,2’-[1,3]dioxan]-5-yl)acetamide (**4b**).

Cream white solid; yield 90%; ^1^H NMR (400 MHz, DMSO-d_6_) δ ppm 10.37 (s, 1H), 10.13 (s, 1H), 7.70 (d, *J* = 2.2 Hz, 1H), 7.46 (dd, *J* = 8.4, 2.2 Hz, 1H), 7.37 (m, 4H), 6.75 (d, *J* = 8.3 Hz, 1H), 4.50 (d, *J* = 10.7 Hz, 2H), 3.61 (s, 2H), 3.46 (d, *J* = 10.8 Hz, 2H), 1.29 (s, 3H), 0.82 (s, 3H). ^13^C NMR (100 MHz, DMSO-d_6_) δ ppm 173.24 (s), 168.89 (s), 137.18 (s), 135.45 (s), 134.56 (s), 131.74 (s), 131.46 (s), 128.69 (s), 127.86 (s), 121.92 (s), 116.49 (s), 110.52 (s), 93.36 (s), 70.61 (s), 42.85 (s), 30.34 (s), 22.76 (s), 22.11 (s). MS(ESI): cald for C_21_H_21_ClN_2_O_4_ [M+H]^+^ 400.86, found 400.94; LC(ESI): t_R_ 2.62 min, purity 98%.

##### N-(5’,5’-dimethyl-2-oxospiro[indoline-3,2’-[1,3]dioxan]-5-yl)-3-morpholinopropanamide (**4c**)

Brown solid; yield 90%; ^1^H NMR (400 MHz, DMSO-d_6_) δ ppm 10.35 (s, 1H), 9.94 (s, 1H), 7.70 (d, *J* = 2.2 Hz, 1H), 6.73 (d, *J* = 8.3 Hz, 1H), 4.50 (d, *J* = 10.8 Hz, 2H), 3.57 (t, *J* = 4.7 Hz, 4H), 3.47 (d, *J* = 10.8 Hz, 2H), 3.16 (dd, *J* = 11.4,5.4 Hz, 1H), 2.89 (s, 1H), 2.69 (s, 6H), 2.40 (s, 6H). ^13^C NMR (100 MHz, DMSO-d_6_) δ ppm 173.24 (s), 170.22 (s), 136.98 (s), 134.72 (s), 127.82 (s), 121.79 (s), 116.46 (s), 110.48 (s), 93.39 (s), 70.61 (s), 66.64 (s), 54.67 (s), 54.67 (s), 54.13 (s), 53.49 (s), 38.72 (s), 34.33 (s), 30.35 (s), 22.75 (s), 22.14 (s), 21.83 (s). MS(ESI): cald for C_20_H_27_N_3_O_5_ [M+H]^+^ 389.45, found 389.15; LC(ESI): t_R_ 1.29 min, purity 97%.

##### 4-chloro-N-(5’,5’-dimethyl-2-oxospiro[indoline-3,2’-[1,3]dioxan]-5-yl)benzamide (**4d**)

Cream white solid; yield 90%; ^1^H NMR (400 MHz, DMSO-d_6_) δ ppm 10.43 (s, 1H), 10.26 (s, 1H), 7.98 (m, 2H), 7.83 (d, *J* = 2.1 Hz, 1H), 7.71 (dd, *J* = 8.4, 2.2 Hz, 1H), 7.61 (m, 2H), 6.81 (d, *J* = 8.4 Hz, 1H), 4.51 (d, *J* = 10.8 Hz, 2H), 3.50 (d, *J* = 10.7 Hz, 2H), 1.32 (s, 3H), 0.83 (s, 3H). ^13^C NMR (100 MHz, DMSO-d_6_) δ ppm 173.28 (s), 164.57 (s), 137.70 (s), 136.82 (s), 134.30 (s), 133.99 (s), 129.99 (s), 128.93 (s), 127.79 (s), 123.36 (s), 117.86 (s), 110.42 (s), 93.42 (s), 70.64 (s), 30.38 (s), 22.79 (s), 22.15 (s). MS(ESI): cald for C_20_H_19_ClN_2_O_4_ [M+H]^+^ 386.83, found 386.15; LC(ESI): t_R_ 2.55 min, purity 96%.

#### Synthesis of intermediates **5a – 5d**

To amide-spiro compounds (**4a – 4d** (421 μmol) was successively added glacial acetic acid (10 mL) and concentrated hydrochloric acid (2 mL). The mixture was stirred for 2 h at room temperature and then poured into water (100 mL), and a precipitate was produced. The precipitate was filtered and purified by recrystallization in methanol to obtain the compound.

##### N-(2,3-dioxoindolin-5-yl)acetamide (**5a**)

Reddish brown solid; yield 90%; ^1^H NMR (400 MHz, DMSO-d_6_) δ ppm 10.92 (s, 1H), 9.98 (s, 1H), 7.80 (d, *J* = 2.2 Hz, 1H), 7.65 (dd, *J* = 8.4, 2.3 Hz, 1H), 6.87 (d, *J* = 8.4 Hz, 1H), 2.03 (s, 3H). MS(ESI): cald for C_10_H_8_N_2_O_3_ [M+H]^+^ 204.19, found 204.33; LC(ESI): t_R_ 0.84 min, purity 90%.

##### 2-(4-chlorophenyl)-N-(2,3-dioxoindolin-5-yl)acetamide (5b)

Yellow soild; yield 90%; MS(ESI): cald for C_16_H_11_ClN_2_O_3_ [M+H]^+^ 314.73, found 314.07; LC(ESI): t_R_ 2.19 min, purity 99%.

##### N-(2,3-dioxoindolin-5-yl)-3-morpholinopropanamide (5c)

Reddish brown; yield 90%; MS(ESI): cald for C_15_H_17_N_3_O_4_ [M+H]^+^ 303.32, found 303.07; LC(ESI): t_R_ 0.28 min, purity 90%.

##### 4-chloro-N-(2,3-dioxoindolin-5-yl)benzamide (5d)

Light brown solid; yield 90%; MS(ESI): cald for C_15_H_9_ClN_2_O_3_ [M+H]^+^ 300.70, found 300.17; LC(ESI): t_R_ 2.19 min, purity 95%.

#### Synthesis of target compounds 6a-6m

Intermediates **5a-5d** (100 mg, 0.333 mmol) and aryl/acylhydrazines (0.399 mmol) were successively added and dissolved in ethanol (5 mL) followed by a dropwise addition of glacial acetic acid (1 mL) under reflux at 80–85 °C. The resulting solution was further stirred for 2 h. After completion of the reaction, 100 mL water was added, and the mixture was filtered under reduced pressure. The solid product was washed with hexane under reduced pressure to obtain the expected target compounds.

##### (E)-N-(2-oxo-3-(2-(p-tolyl)hydrazineylidene)indolin-5-yl)acetamide (6a)

Yellow soild; yield 90%; ^1^H NMR (400 MHz, DMSO-d_6_) δ ppm 12.75 (s, 1H), 10.90 (s, 1H), 9.88 (s, 1H), 7.87 (d, *J* = 2.1 Hz, 1H), 7.33 (m, 3H), 7.19 (d, *J* = 8.2 Hz, 2H), 6.85 (d, *J* = 8.4 Hz, 1H), 2.29 (s, 3H), 2.04 (s, 3H). MS(ESI): cald for C_17_H_16_N_4_O_2_ [M+H]^+^ 308.34, found 309.17; LC(ESI): t_R_ 2.40 min, purity 96%.

##### (E)-N-(3-(2-(2,4-dichlorophenyl)hydrazineylidene)-2-oxoindolin-5-yl)acetamide (6b)

Orange soild; yield 90%; ^1^H NMR (400 MHz, DMSO-d_6_) δ ppm 13.10 (s, 1H), 11.10 (s, 1H), 9.92 (s, 1H), 7.96 (d, *J* = 2.1 Hz, 1H), 7.74 (d, *J* = 8.9 Hz, 1H), 7.68 (d, *J* = 2.3 Hz, 1H), 7.49 (dd, *J* = 8.8, 2.4 Hz, 1H), 7.49 (dd, *J* = 8.4, 2.2 Hz, 1H), 6.89 (d, *J* = 8.8 Hz, 1H), 2.04 (s, 3H). MS(ESI): cald for C_16_H_12_Cl_2_N_4_O_2_ [M-H]^−^ 363.20, found 363.95; LC(ESI): t_R_ 2.68 min, purity 95%.

##### (E)-N-(2-oxo-3-(2-(pyridine-4-yl)hydrazineylidene)indolin-5-yl)acetamide (6c)

Orange; yield 90%; ^1^H NMR (400 MHz, DMSO-d_6_) δ ppm 12.69 (s, 1H), 11.06 (s, 1H), 9.94 (s, 1H), 8.44 (d, *J* = 5.7 Hz, 2H), 7.94 (d, *J* = 2.0 Hz, 1H), 7.41 (dd, *J* = 13.3, 4.2 Hz, 3H), 6.87 (d, *J* = 8.4 Hz, 1H), 2.05 (s, 3H). ^13^C NMR (100 MHz, DMSO-d_6_) δ ppm 168.53 (s), 163.47 (s), 149.95 (s), 137.15 (s), 134.80 (s), 132.51 (s), 121.62 (s), 120.95 (s), 111.58 (s), 111.30 (s), 109.30 (s), 24.35 (s), 21.52 (s). MS(ESI): cald for C_15_H_13_N_5_O_2_ [M+H^]+^ 295.30, found 295.07; LC(ESI): t_R_ 85 min, purity 85%.

##### (E)-2-(4-chlorophenyl)-N-(2-oxo-3-(2-(p-tolyl)hydrazineylidene)indolin-5-yl)acetamide (6d)

Yellow solid; yield 90%; ^1^H NMR (400 MHz, DMSO-d_6_) δ ppm 12.73 (s, 1H), 10.92 (s, 1H), 10.14 (s, 1H), 7.90 (d, *J* = 2.1 Hz, 1H), 7.40 (d, *J* = 8.6 Hz, 2H), 7.36 (m, 3H), 7.30 (m, 2H), 7.19 (d, *J* = 8.4 Hz, 2H), 6.86 (d, *J* = 8.4 Hz, 1H), 3.64 (s, 2H), 2.28 (s, 3H). ^13^C NMR (100 MHz, DMSO-d_6_) δ ppm 168.88 (s), 163.86 (s), 140.65 (s), 135.90 (s), 135.55 (s), 134.24 (s), 132.59 (s), 131.74 (s), 131.50 (s), 130.43 (s), 128.69 (s), 127.62 (s), 121.79 (s), 119.96 (s), 114.58 (s), 110.93 (s), 110.48 (s), 42.89 (s), 20.86 (s). MS(ESI): cald for C_23_H_19_ClN_4_O_2_ [M+H]^+^ 418.88, found 418.04; LC(ESI): t_R_ 2.84 min, purity 98%.

##### (E)-2-(4-chlorophenyl)-N-(3-(2-(2,4-dichlorophenyl)hydrazineylidene)-2-oxoindolin-5-yl)acetamide (6e)

Orange solid; yield 90%; ^1^H NMR (400 MHz, DMSO-d_6_) δ ppm 13.06 (s, 1H), 11.12 (s, 1H), 10.18 (s, 1H), 7.99 (d, *J* = 2.1 Hz, 1H), 7.74 (d, *J* = 8.9 Hz, 1H), 7.68 (d, *J* = 2.4 Hz, 2H), 7.47 (dd, *J* = 8.9, 2.4 Hz, 2H), 7.38 (qd, *J* = 8.4, 7.3, 2.4 Hz, 5H), 3.64 (s, 2H). ^13^C NMR (100 MHz, DMSO-d_6_) δ ppm 168.96 (s), 164.00 (s), 138.47 (s), 136.94 (s), 135.49 (s), 134.54 (s), 131.75 (s), 131.51 (s), 129.43 (s), 129.26 (s), 128.69 (s), 126.54 (s), 121.30 (s), 120.89 (s), 119.24 (s), 115.66 (s), 111.41 (s), 111.37 (s), 42.85 (s). MS(ESI): cald for C_22_H_15_Cl_3_N_4_O_2_ [M+H]^+^ 473.74, found 473.93; LC(ESI): t_R_ 3.10 min, purity 90%.

##### (E)-2-(4-chlorophenyl)-N-(2-oxo-3-(2-(4(trifluoromethyl)phenyl)hydrazineylidene)indolin-5-yl)acetamide (6f)

Yellow solid; yield 90%; ^1^H NMR (400 MHz, DMSO-d_6_) δ ppm 12.76 (s, 1H), 11.02 (s, 1H), 10.18 (s, 1H), 7.95 (d, *J* = 2.1 Hz, 1H), 7.71 (d, *J* = 8.5 Hz, 2H), 7.58 (d, *J* = 8.5 Hz, 2H), 7.40 (m, 5H), 6.88 (d, *J* = 8.4 Hz, 1H), 3.65 (s, 2H). ^13^C NMR (100 MHz, DMSO-d_6_) δ ppm 171.17 (s), 165.81 (s), 148.52 (s), 139.02 (s), 137.71 (s), 136.66 (s), 133.97 (s), 133.72 (s), 132.70 (s), 130.91 (s), 129.43 (s), 123.54 (s), 123.23 (s), 116.86 (s), 113.40 (s), 45.07 (s). MS(ESI): cald for C_23_H_16_ClF_3_N_4_O_2_ [M+H]^+^ 472.85, found 472.03; LC(ESI): t_R_ 2.90 min, purity 98%.

##### (E)-3-morpholino-N-(2-oxo-3-(2-(p-tolyl)hydrazineylidene)indolin-5-yl)acetamide (6g)

Yellow solid; yield 90%; ^1^H NMR (400 MHz, DMSO-d_6_) δ ppm 12.74 (s, 1H), 10.90 (s, 1H), 9.95 (s, 1H), 7.91 (d, *J* = 2.1 Hz, 1H), 7.19 (d, *J* = 8.1 Hz, 2H), 6.85 (d, *J* = 8.4 Hz, 1H), 3.58 (m, 5H), 2.63 (t, *J* = 7.0 Hz, 2H), 2.47 (d, *J* = 6.9 Hz, 2H), 2.41 (s, 5H), 2.34 (s, 1H), 2.29 (s, 3H). ^13^C NMR (100 MHz, DMSO-d_6_) δ ppm 170.23 (s), 163.88 (s), 140.68 (s), 135.74 (s), 134.43 (s), 132.56 (s), 130.44 (s), 127.72 (s), 121.75 (s), 119.86 (s), 114.57 (s), 110.90 (s), 110.41 (s), 66.68 (s), 54.74 (s), 53.54 (s), 34.37 (s), 20.87 (s). MS(ESI): cald for C_22_H_25_N_5_O_3_ [M+H]^+^ 407.47, found 407.14; LC(ESI): t_R_ 1.92 min, purity 97%.

##### (E)-N-(3-(2-(2,4-dichlorophenyl)hydrazineylidene)-2-oxoindolin-5-yl)-3-morpholinopropanamide (6h)

Orange solid; yield 90%; ^1^H NMR (400 MHz, DMSO-d_6_) δ ppm 13.09 (s, 1H), 11.13 (s, 1H), 10.09 (s, 1H), 8.01 (d, *J* = 2.1 Hz, 1H), 7.71 (m, 2H), 7.50 (dd, *J* = 8.9, 2.4 Hz, 1H), 7.37 (dd, *J* = 8.4, 2.2 Hz, 1H), 6.91 (d, *J* = 8.4 Hz, 1H), 3.71 (s, 8H), 3.04 (s, 1H), 2.66 (s, 2H). ^13^C NMR (100 MHz, DMSO-d_6_) δ ppm 164.02 (s), 138.47 (s), 136.94 (s), 134.44 (s), 131.79 (s), 129.47 (s), 129.27 (s), 126.57 (s), 121.28 (s), 120.89 (s), 119.28 (s), 115.57 (s), 111.44 (s), 111.35 (s), 52.72 (s). MS(ESI): cald for C_21_H_21_Cl_2_N_5_O_3_ [M+H]^+^ 462.33, found 462.04; LC(ESI): t_R_ 2.13 min, purity 90%.

##### (E)-3-morpholino-N-(2-oxo-3-(2-(4(trifluoromethyl)phenyl)hydrazineylidene)indolin-5-yl)propanamide (6i)

Yellow solid; yield 90%; ^1^H NMR (400 MHz, DMSO-d_6_) δ ppm 12.78 (s, 1H), 11.06 (s, 1H), 10.55 (s, 1H), 7.97 (d, *J* = 2.1 Hz, 1H), 7.72 (d, *J* = 8.4 Hz, 2H), 7.58 (d, *J* = 8.4 Hz, 2H), 7.40 (dd, *J* = 8.4, 2.2 Hz, 1H), 6.90 (d, *J* = 8.4 Hz, 1H), 3.99 (d, *J* = 17.4 Hz, 2H), 3.36 (s, 2H), 3.43 (d, *J* = 10.3 Hz, 4H), 3.12 (s, 2H), 2.91 (s, 2H). ^13^C NMR (100 MHz, DMSO-d_6_) δ ppm 163.61 (s), 146.32 (s), 136.93 (s), 130.52 (s), 127.26 (s), 126.38 (s), 121.33 (s), 121.10 (s), 114.64 (s), 111.25 (s), 51.84 (s). MS(ESI): cald for C_22_H_22_F_3_N_5_O_3_ [M+H]^+^ 461.45, found 461.04; LC(ESI): t_R_ 2.04 min, purity 97%.

##### (E)-4-chloro-N-(2-oxo-3-(2-(p-tolyl)hydrazineylidene)indolin-5-yl)benzamide (6j)

Yellow soild; yield 90%; ^1^H NMR (400 MHz, DMSO-d_6_) δ ppm 12.75 (s, 1H), 10.97 (s, 1H), 10.27 (s, 1H), 8.02 (dd, *J* = 8.2, 1.7 Hz, 3H), 7.60 (m, 3H), 7.33 (m, 2H), 7.20 (d, *J* = 8.2 Hz, 2H), 6.91 (d, *J* = 8.4 Hz, 1H), 2.29 (s, 3H). ^13^C NMR (100 MHz, DMSO-d_6_) δ ppm 164.54 (s), 163.91 (s), 140.69 (s), 136.78 (s), 136.35 (s), 134.09 (s), 134.01 (s), 132.61 (s), 130.44 (s), 130.00 (s), 128.94 (s), 127.64 (s), 121.75 (s), 121.30 (s), 114.60 (s), 111.81 (s), 110.82 (s), 20.87 (s). MS(ESI): cald for C_22_H_17_ClN_4_O_2_ [M+H]^+^ 404.85, found 404.04; LC(ESI): t_R_ 2.94 min, purity 98%.

##### (E)-4-chloro-N-(3-(2-(2,4-dichlorophenyl)hydrazineylidene)-2-oxoindolin-5-yl)benzamide (6k)

Yellow solid; yield 90%; ^1^H NMR (400 MHz, DMSO-d_6_) δ ppm 13.09 (s, 1H), 11.17 (s, 1H), 10.31 (s, 1H), 8.11 (d, *J* = 2.1 Hz, 1H), 8.01 (d, *J* = 8.3 Hz, 2H), 7.76 (d, *J* = 8.9 Hz, 1H), 7.71 (s, 2H), 7.69 (d, *J* = 2.3 Hz, 1H), 7.62 (dd, *J* = 8.9, 2.3 Hz, 3H). ^13^C NMR (100 MHz, DMSO-d_6_) δ ppm 164.59 (s), 164.07 (s), 138.49 (s), 137.38 (s), 136.85 (s), 134.33 (s), 133.99 (s), 131.76 (s), 130.01 (s), 129.45 (s), 129.26 (s), 128.96 (s), 126.56 (s), 122.62 (s), 120.84 (s), 119.26 (s), 115.63 (s), 112.65 (s), 111.29 (s). MS(ESI): cald for C_22_H_13_C_l3_N_4_O_2_ [M+H]^+^ 459.71, found 459.74; LC(ESI): t_R_ 3.01 min, purity 85%.

##### (E)-4-chloro-N-(2-oxo-3-(2-(4(trifluoromethyl)phenyl)hydrazineylidene)indolin-5-yl)benzamide (6l).

Yellow solid; yield 90%; ^1^H NMR (400 MHz, DMSO-d_6_) δ ppm 12.79 (s, 1H), 11.08 (s, 1H), 10.31 (s, 1H), 8.08 (d, *J* = 2.1 Hz, 1H), 8.02 (m, 2H), 7.72(d, *J* = 8.4 Hz, 2H), 7.62 (m, 5H), 6.93 (d, *J* = 8.6 Hz, 1H). ^13^C NMR (100 MHz, DMSO-d_6_) δ ppm 164.58 (s), 163.66 (s), 146.35 (s), 137.25 (s), 136.83 (s), 134.24 (s), 134.02 (s), 130.53 (s), 130.01 (s), 128.96 (s), 127.20 (s), 122.36 (s), 121.29 (s), 114.67 (s), 112.49 (s), 111.09 (s). MS(ESI): cald for C_22_H_14_ClF_3_N_4_O_2_ [M+H]^+^ 458.83, found 458.94; LC(ESI): t_R_ 3.01 min, purity 95%.

##### (E)-4-chloro-N-(2-oxo-3-(2-(pyridine-4-yl)hydrazineylidene)indolin-5-yl)benzamide (6m)

Orange; yield 90%; ^1^H NMR (400 MHz, DMSO-d_6_) δ ppm 12.75 (s, 1H), 10.90 (s, 1H), 9.88 (s, 1H), 7.87 (d, *J* = 2.1 Hz, 1H), 7.33 (m, 3H), 7.19 (d, *J* = 8.2 Hz, 2H), 6.85 (d, *J* = 8.4 Hz, 1H), 2.29 (s, 3H), 2.04 (s, 3H). ^13^C NMR (100 MHz, DMSO-d_6_) δ ppm 164.63 (s), 163.57 (s), 150.91 (s), 149.37 (s), 137.63 (s), 136.85 (s), 134.29 (s), 133.99 (s), 131.75 (s), 130.02 (s), 128.97 (s), 122.88 (s), 121.08 (s), 112.88 (s), 111.19 (s), 109.23 (s). MS(ESI): cald for C_20_H_14_ClN_5_O_2_ [M+H]^+^ 391.82, found 391.05; LC(ESI): t_R_ 1.78 min, purity 90%.

### Biochemical assays

#### AlphaScreen binding assays

AlphaScreen assays were performed as described previously [37]. For RBD-ACE2 assays, 2 nM of ACE2-Fc (Sino Biological, Chesterbrook, PA, USA) was incubated with 5 nM HIS-tagged SARS-CoV-2 Spike-RBDs representing the parental USA-WA/2020 (“Wild-type” (WT)) sequence (SinoBiological) in the presence of 5 μg/mL nickel chelate donor bead in a total of 10 μL of 20 mM Tris (pH 7.4), 150 mM KCl, and 0.05% CHAPS. Test compounds were diluted to 100x final concentration in DMSO. 5 μL of ACE2-Fc/Protein A acceptor bead was first added to the reaction, followed by 100 nL test compound and then 5 μL of RBD-HIS/Nickel chelate donor beads. All conditions were performed in duplicate. Following incubation at room temperature for 2 h, luminescence signals were measured using a ClarioStar plate reader (BMC Labtech, Cary, NC, USA). Data were then normalized to percent inhibition, where 100% equaled the AlphaScreen signal in the absence of RBD-HIS, and 0% denoted AlphaScreen signal in the presence of both protein and DMSO vehicle control. To measure PD1/PD-L1 binding, 0.5 nM of human PD-L1-Fc (Sino Biological) was incubated with 5 nM HIS-tagged human PD1 (Sino Biological) in the presence of 5 μg/mL protein A and 5 μg/mL nickel chelate donor beads in a total volume of 10 μL of 20 mM HEPES (pH 7.4), 150 mM NaCl, and 0.005% Tween-20. Proteins and test agents were then added, incubated, and analyzed as described above.

#### In vitro bioassay against Aurora A kinase

The activities of the synthesized compounds were evaluated using Invitrogen Z′-LYTE® Kinase Assay Kitser/thr 01 peptide. The bioassay was performed using Aurora A kinase at an optimum concentration of 2.7–4.7 nM and ATP at a concentration of 500 μM. Stock solutions of molecules were prepared at 10 mM in DMSO, and then serially diluted in buffer solution to yield final hit concentrations ranging from 0.01 μM to 100 μM. DMSO did not exceed 1% in the final kinase reaction (10 μL). The percentage of inhibition and IC_50_ value were calculated using nonlinear regression of the log (concentration) vs inhibition percentage values using GraphPad Prism 7.04.

### Computer modeling

#### Selection of crystal structure of Spike/ACE2 and Aurora A kinase receptor

In this study, the need for high resolution and well-defined domain completeness led to the selection of two protein crystal structures among the several protein crystal structures present in the Protein Data Bank (PDB) [49] with PDB IDs: 6M0J and 4BYI. For the spike/ACE2 complex, 6M0J crystal structure, bound metallic cofactors (Zn^2+^ and Cl^−^), *N*-Acetyl glucosamine (NAG), and water molecules were chosen [50]. Meanwhile, 4BYI was chosen for Aurora A kinase. This structure was found to have a co-crystalized ligand, imidazo[4,5-b]pyridine in its structure [50].

#### Molecular docking procedures

Generally, molecular docking procedures were performed using similar methods as reported in our previous published papers [51–53].

#### Ligand preparation

The software Molecular Operating Environment (MOE) [54] was used to generate the 3D structures of all ligands. For these ligands, all possible tautomeric forms were generated using the LigPrep tool implemented in the Maestro package licensed by Schrödinger (version 2017.2) [55]. These ligands were further energy minimized using the integrated Optimised Potentials for Liquid Simulations (OPLS_2005) forced field [56]. At the end of this ligand preparation, 60 conformers were computed for each molecule using the ConfGen tool implemented in the Schrodinger package, while allowing the minimization of the output conformations, and allowing the default settings of all other parameters [57].

#### Protein preparation

The crystal structures of the SARS-CoV-2 viral spike/ACE2 complex (PDB ID: 6M0J), which is the Wuhan variant, along with the human Aurora A kinase (PDB ID: 4BYI) were downloaded from the Protein Data Bank (PDB; www.rcsb.org) [49]. All water molecules were deleted using MOE software [54]. Further preparation of the protein structures preparation was done using the Protein Preparation Wizard of Schrödinger software (version 2017.2) [55]. Here, bond orders were assigned, hydrogen atoms were added, missing side chains were filled using PRIME implemented in the Maestro package, while the H-bond network was subsequently optimized. The protonation states at pH 7.0 were predicted using the Epik tool in the Maestro package commercialized by Schrödinger (version 2017.2) [55]. The structures were finally [58] subjected to a restrained energy minimization step (RMSD of the atom displacement for terminating the minimization was 0.3 Å) using the OPLS2005 force field [57].

#### Docking towards the SARS-CoV-2 Spike RBD/ACE2 and the human Aurora A kinase

Docking procedures were performed similarly using the Glide program as previously demonstrated [52–54]. Two grid boxes were generated, one for the SARS-CoV-2 viral protein RBD/ACE2 human receptor (PDB ID: 6M0J) using specific protein residues and one grid box for the human protein Aurora A kinase (PDB ID: 4BYI) taking the co-crystallized ligand in the protein as the centroid [39]. For the ACE2/SARS-CoV-2 protein (PDB ID: 6M0J), the whole structure was explored for the generation of the grid to know where the ligands would preferably bind. Hence, the following amino acids; Asp597, Thr598, Lys516, Val321, Gln121, Lys578, Ala283, Ser91, Asn746, Gln68, Pro744, Glu518, and Thr610 were used for the generation of the docking grid around the SARS-CoV-2/ACE2 protein, that is at the angiotensin II binding site [59]. For both generated grid boxes, the sides were set to 36 Å. The generated 3D conformers of the prepared ligand were docked into the different receptor grid files. For the docking process, default settings were used except input ring conformation as well as writing a total of 10 poses per ligand conformer from the 20 retained poses that were included for each ligand conformer. The GlideScore Standard Precision (SP) mode was used as the scoring function [44].

### Supplementary information

Supplementary Data
