# Supplementary material for: Design, synthesis, and biochemical and computational screening of novel oxindole derivatives as inhibitors of Aurora A kinase and SARS-CoV-2 spike/host ACE2 interaction
Source: Med Chem Res. 2024 Mar 5;33(4):620–34. doi: 10.1007/s00044-024-03201-7 (PMC11024012; doi:10.1007/s00044-024-03201-7)

**SUPPLEMENTARY DATA**

**Design, synthesis, and biochemical and computational screening of novel oxindole derivatives as inhibitors of Aurora A kinase and SARS-CoV-2 spike/host ACE2 interaction**

Donatus B. Eni^1,2*^, Joel Cassel^3^, Cyril T. Namba-Nzanguim^1,2^, Conrad V. Simoben^1^, Ian Tietjen^3^, Ravikumar Akunuri^3^, Joseph M. Salvino^3*^, Fidele Ntie-Kang^1,2,4*^

^1^Center for Drug Discovery, Faculty of Science, University of Buea, Buea, Cameroon. ^2^Department of Chemistry, Faculty of Science, University of Buea, Buea, Cameroon.

^3^The Wistar Institute, Philadelphia, Pennsylvania, PA, USA

^4^Institute of Pharmacy, Martin-Luther University Halle-Wittenberg, Halle (Saale), Germany

*Corresponding author: [jsalvino@wistar.org](mailto:jsalvino@wistar.org) (JMS); [fidele.ntie-kang@ubuea.cm](mailto:fidele.ntie-kang@ubuea.cm) (FNK)

**Author ORCID**

Donatus B. Eni: 0000-0002-4148-6372

Joel Cassel: 0000-0001-8465-8739

Cyril T. Namba-Nzanguim: 0000-0002-8404-7214

Conrad V. Simoben: 0000-0002-5958-6961

Ian Tietjen: 0000-0002-8991-6490

Ravikumar Akunuri: 0000-0003-4898-5753

Joseph M. Salvino: 0000-0002-2184-5980

Fidele Ntie-Kang: 0000-0003-0795-394X


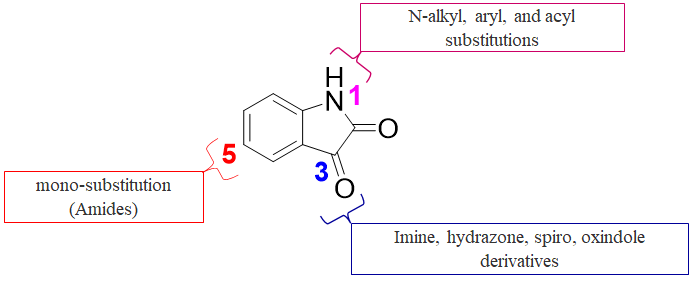


**Fig. S1:** The various targets modification positions on the isatin scaffold


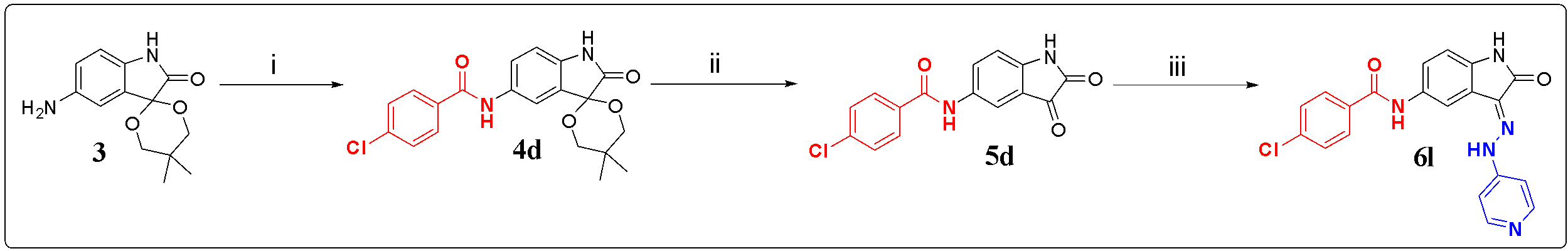


**Scheme S1:** Synthesis of compound **6m.**

**CODES IN THE LAB NOTEBOOK AND THE ARTICLE**

| **S/N** | **CODE IN LAB NOTEBOOK** | **CODE IN ARTICLE** |
| --- | --- | --- |
| 1 | DBE-01-023 or DBE-Glyol | 2 |
| 2 | DBE-01-025 | 3 |
| 3 | DBE-01-027 | 4a |
| 4 | DBE-01-045 | 4b |
| 5 | DBE-01-053 | 4c |
| 6 | DBE-01-033 | 4d |
| 7 | DBE-01-041 | 4e |
| 8 | DBE-01-031 | 6a |
| 9 | DBE-01-043 | 6b |
| 10 | DBE-01-037 | 6c |
| 11 | DBE-01-049 | 6d |
| 12 | DBE-01-051 | 6e |
| 13 | DBE-01-061 | 6g |
| 14 | DBE-01-063 | 6h |
| 15 | DBE-01-065 | 6i |
| 16 | DBE-01-067 | 6j |
| 17 | DBE-01-071 | 6k |
| 18 | DBE-01-069 | 6l |
| 19 | DBE-01-039 | 6m |

**C-13 NMR DATA**


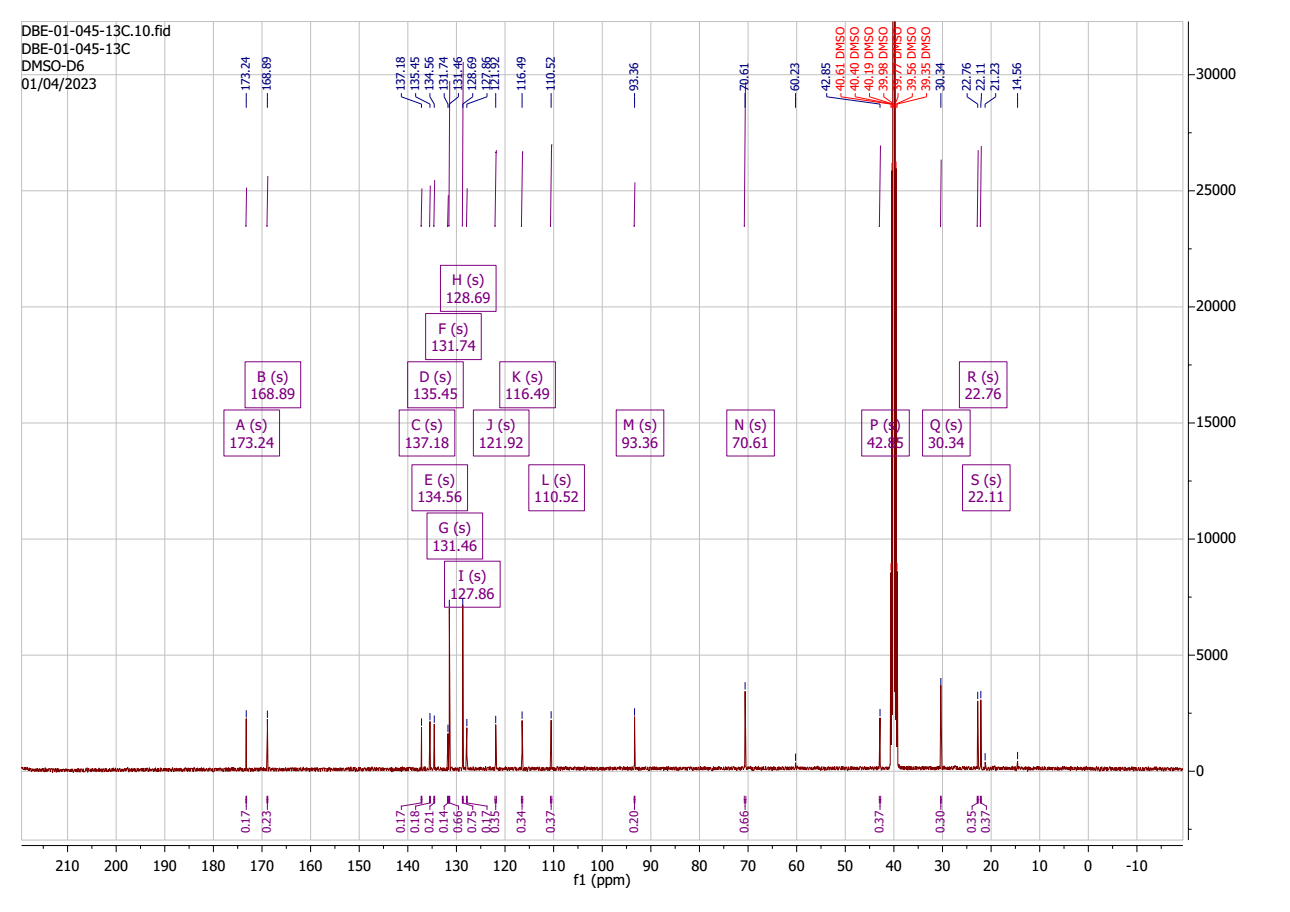


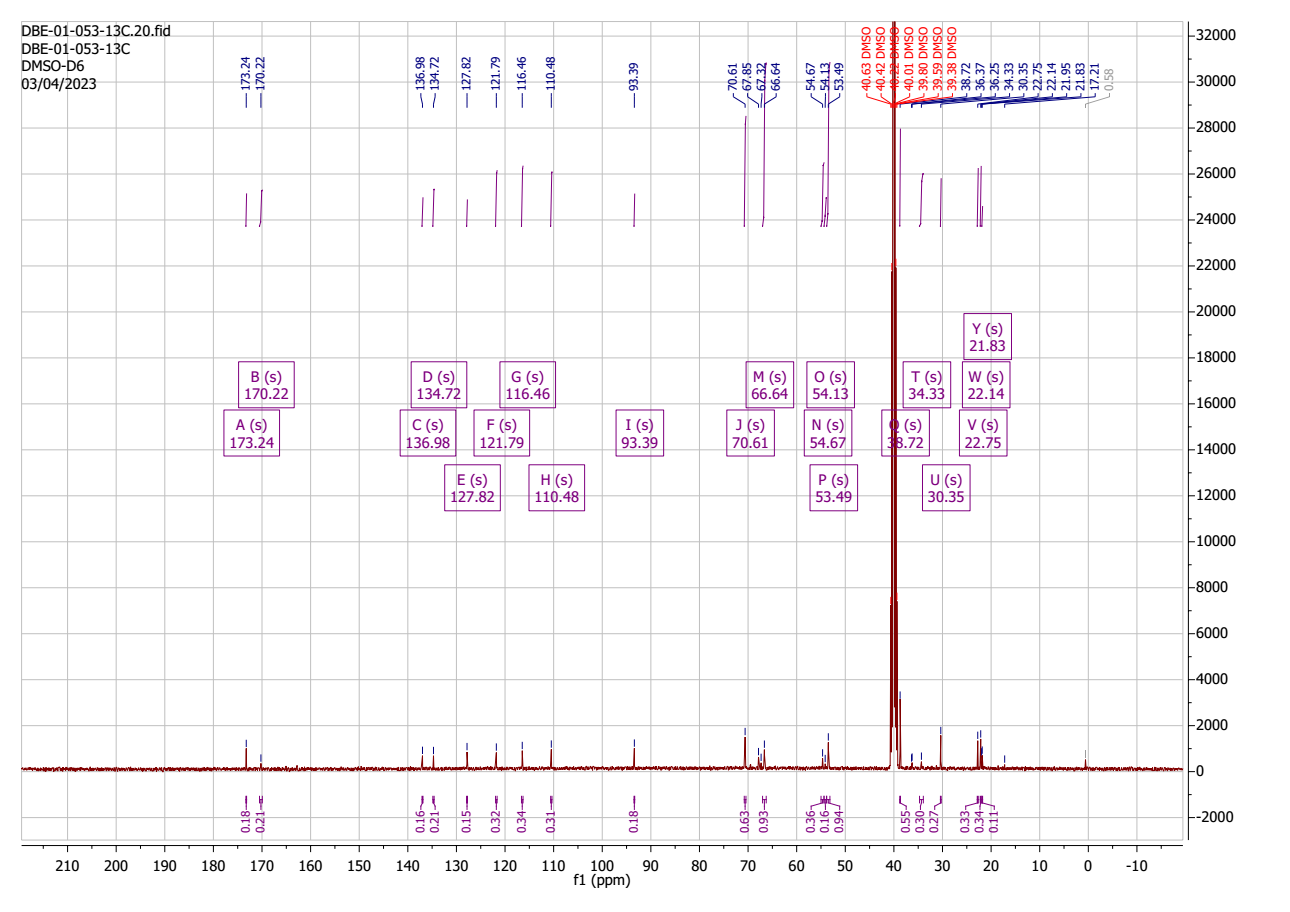


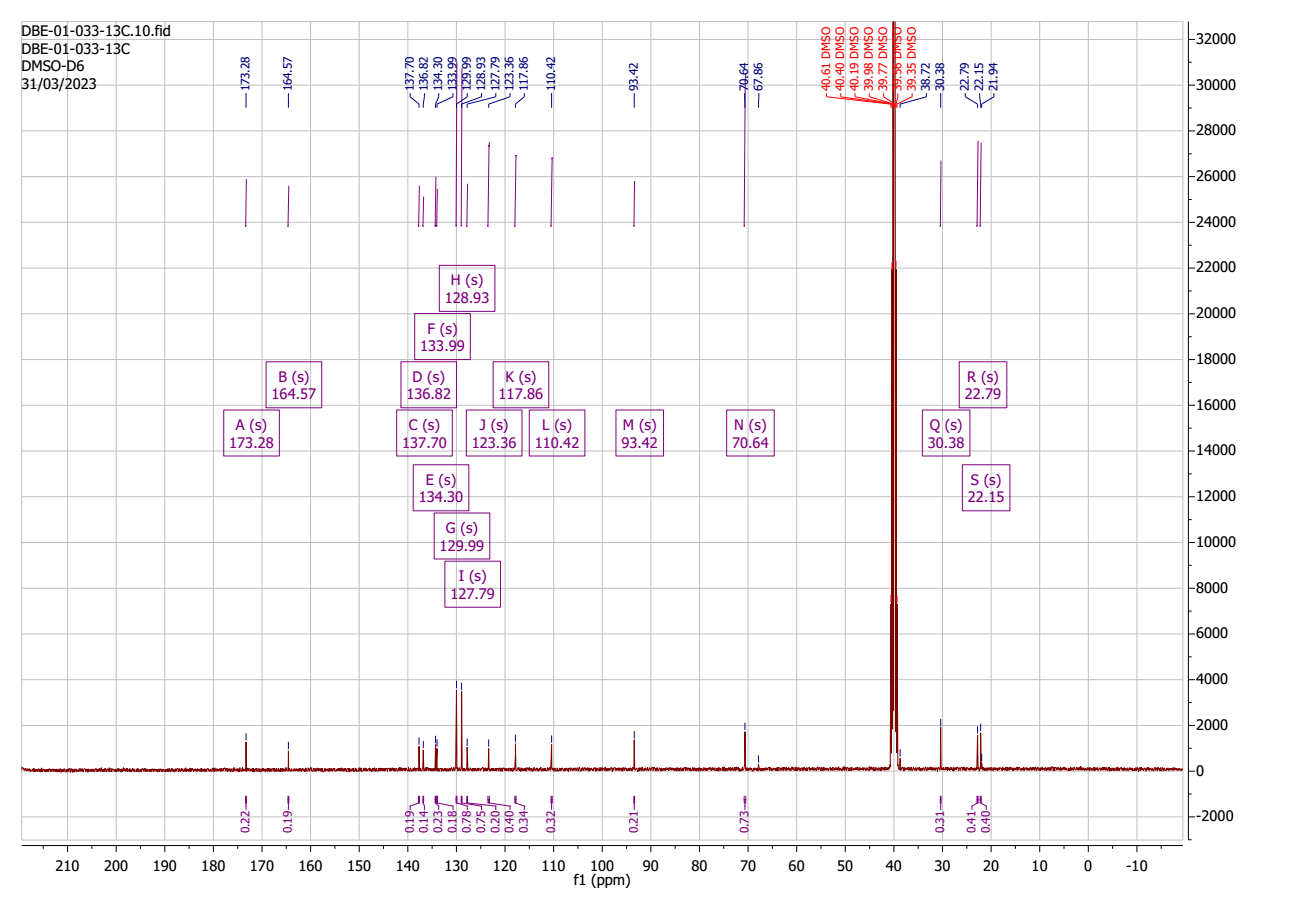

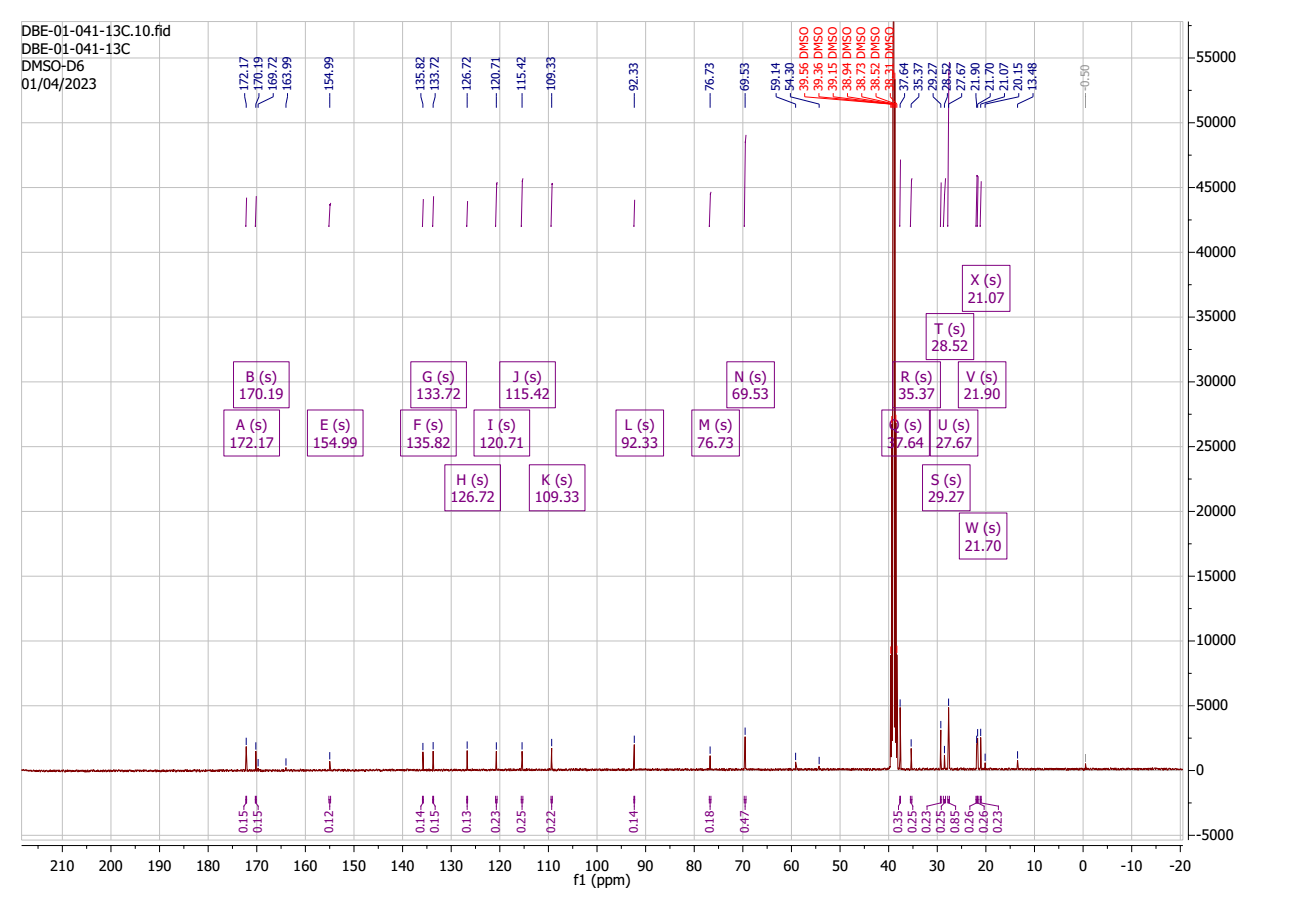


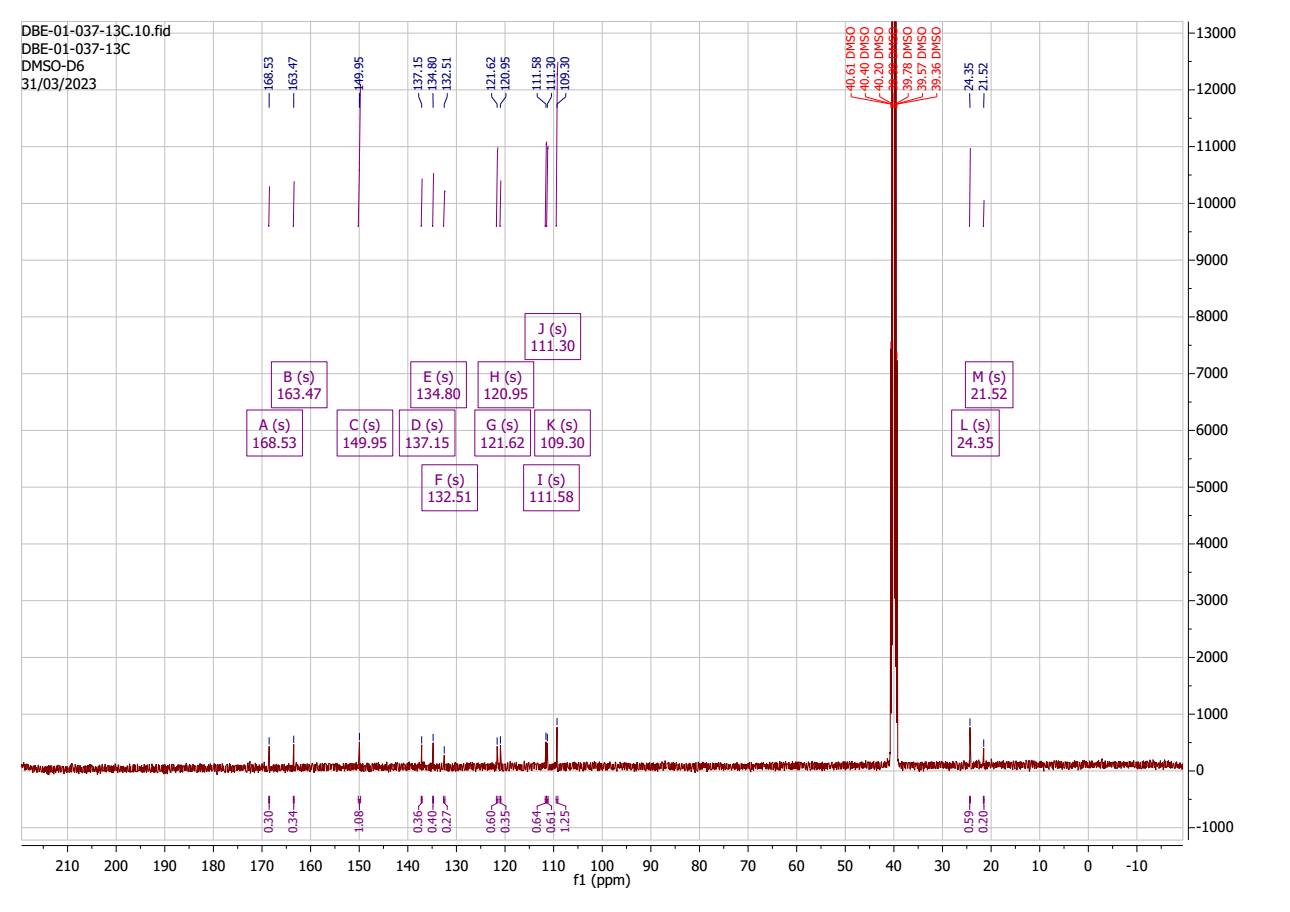


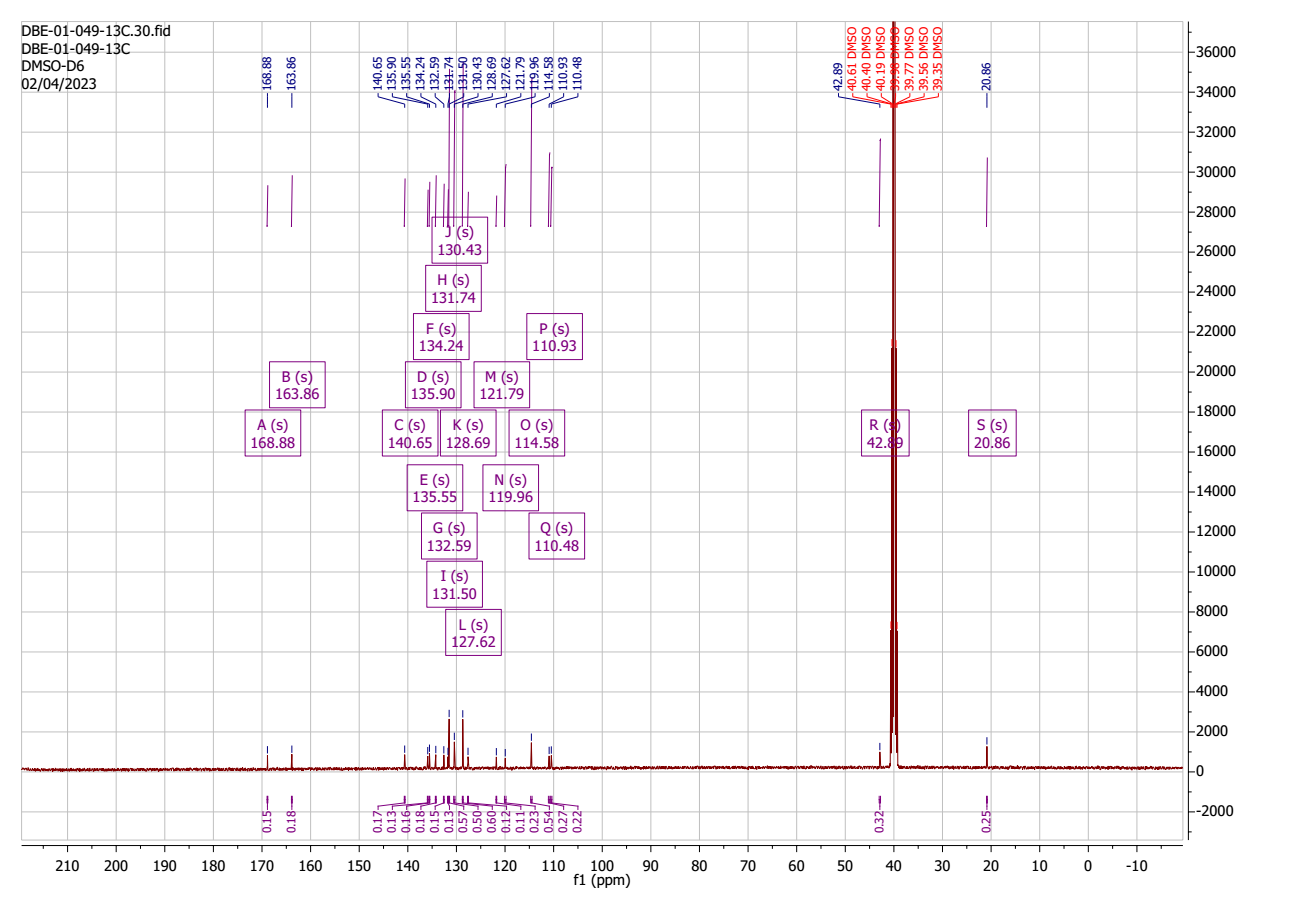


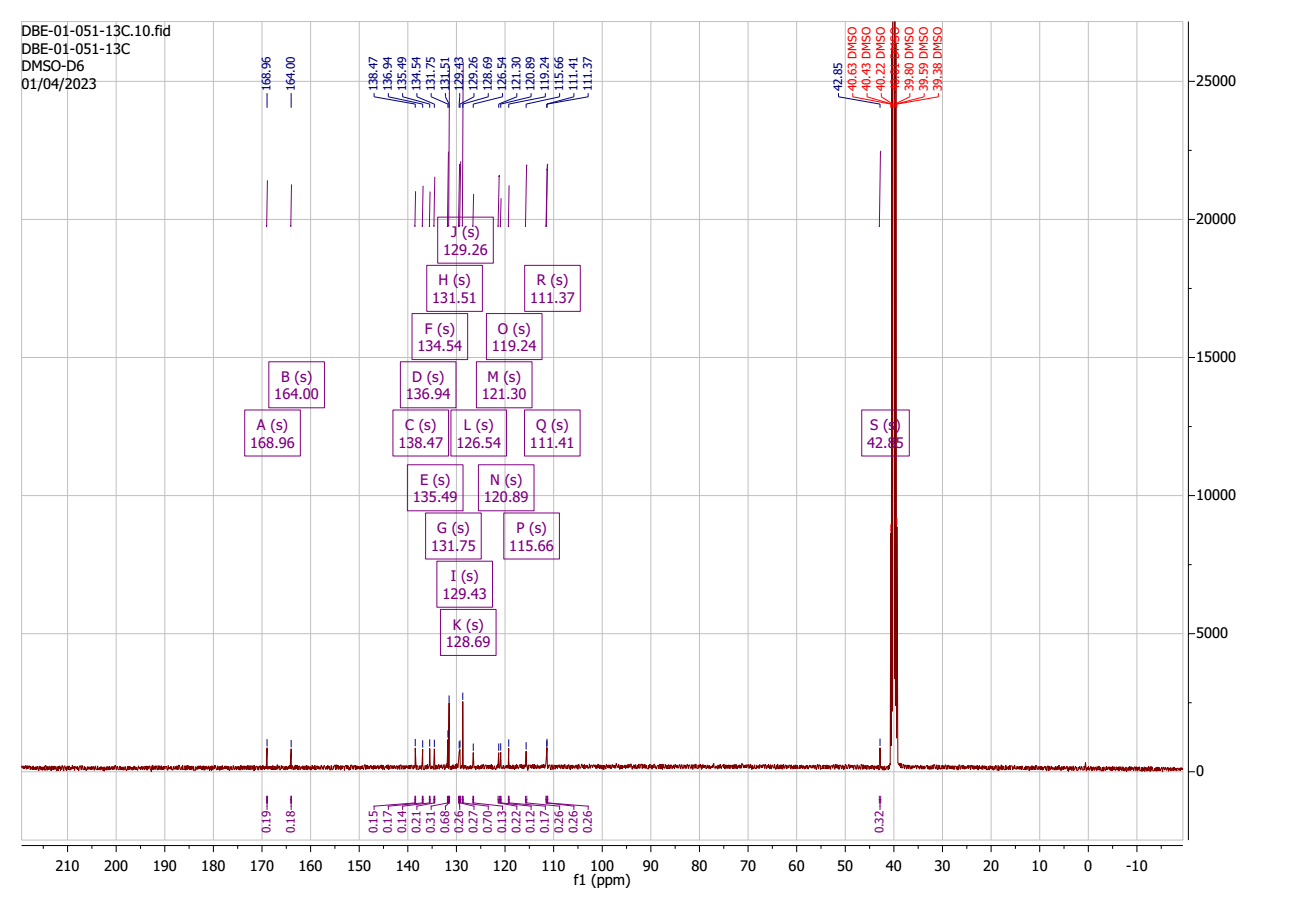


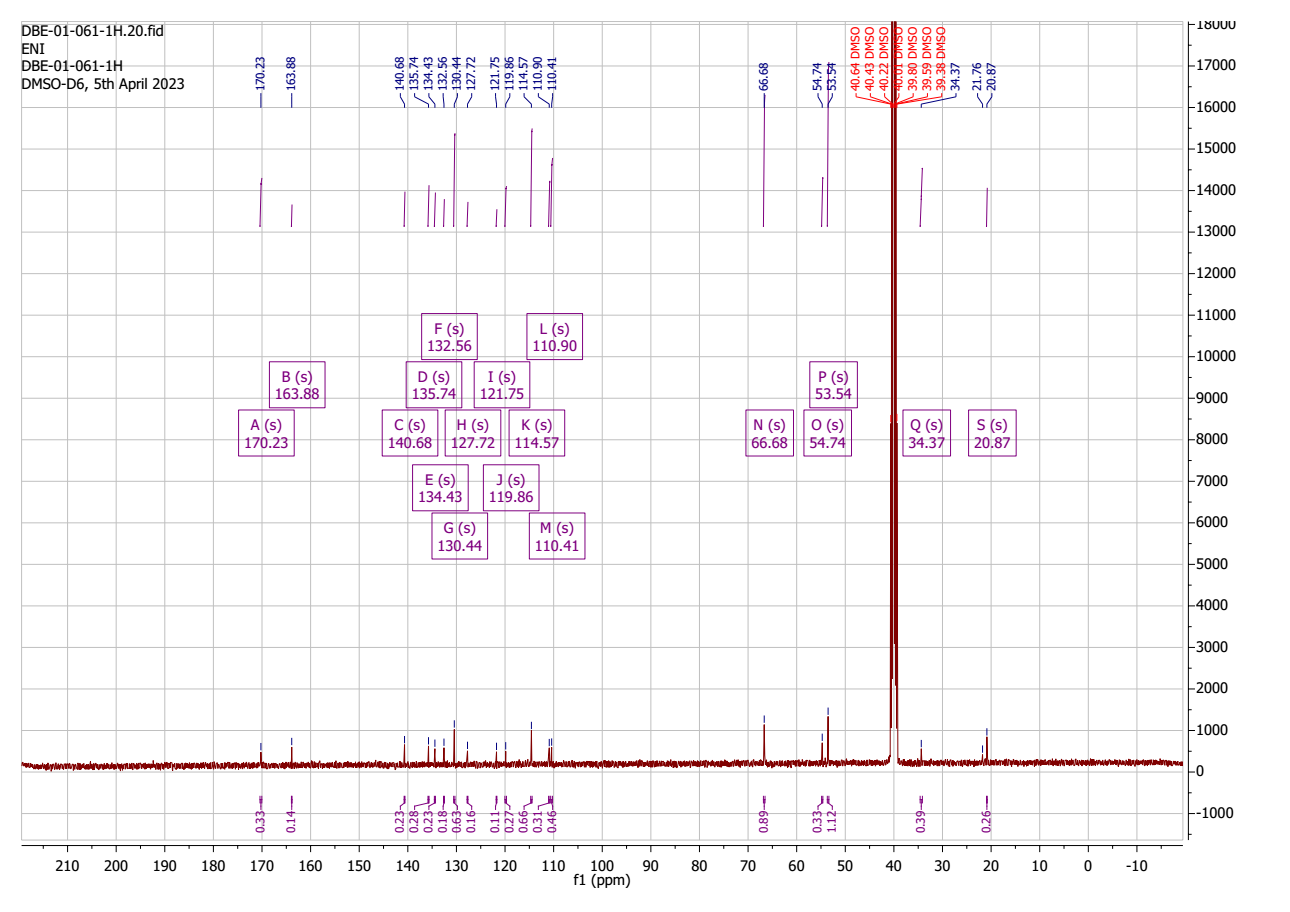


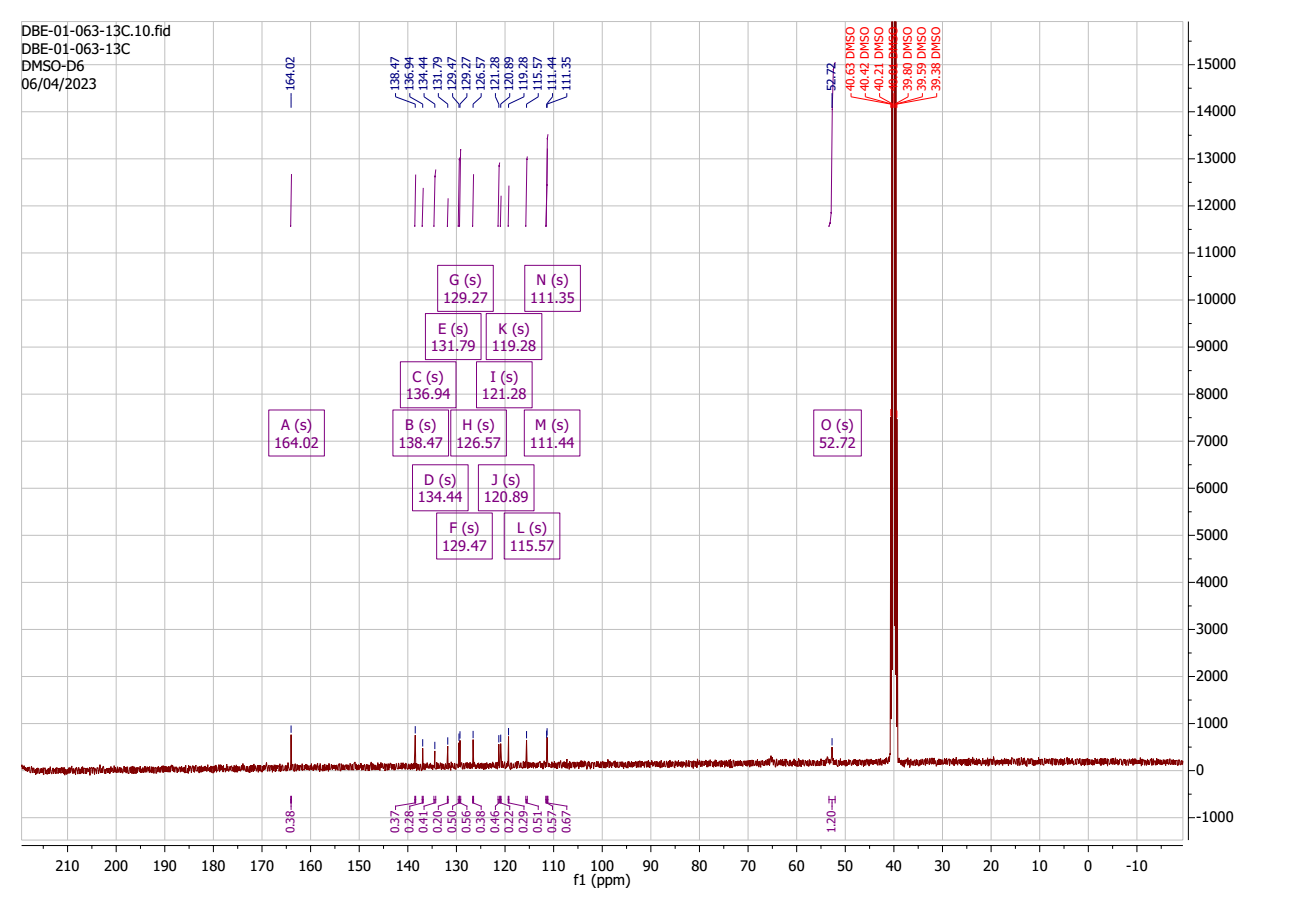


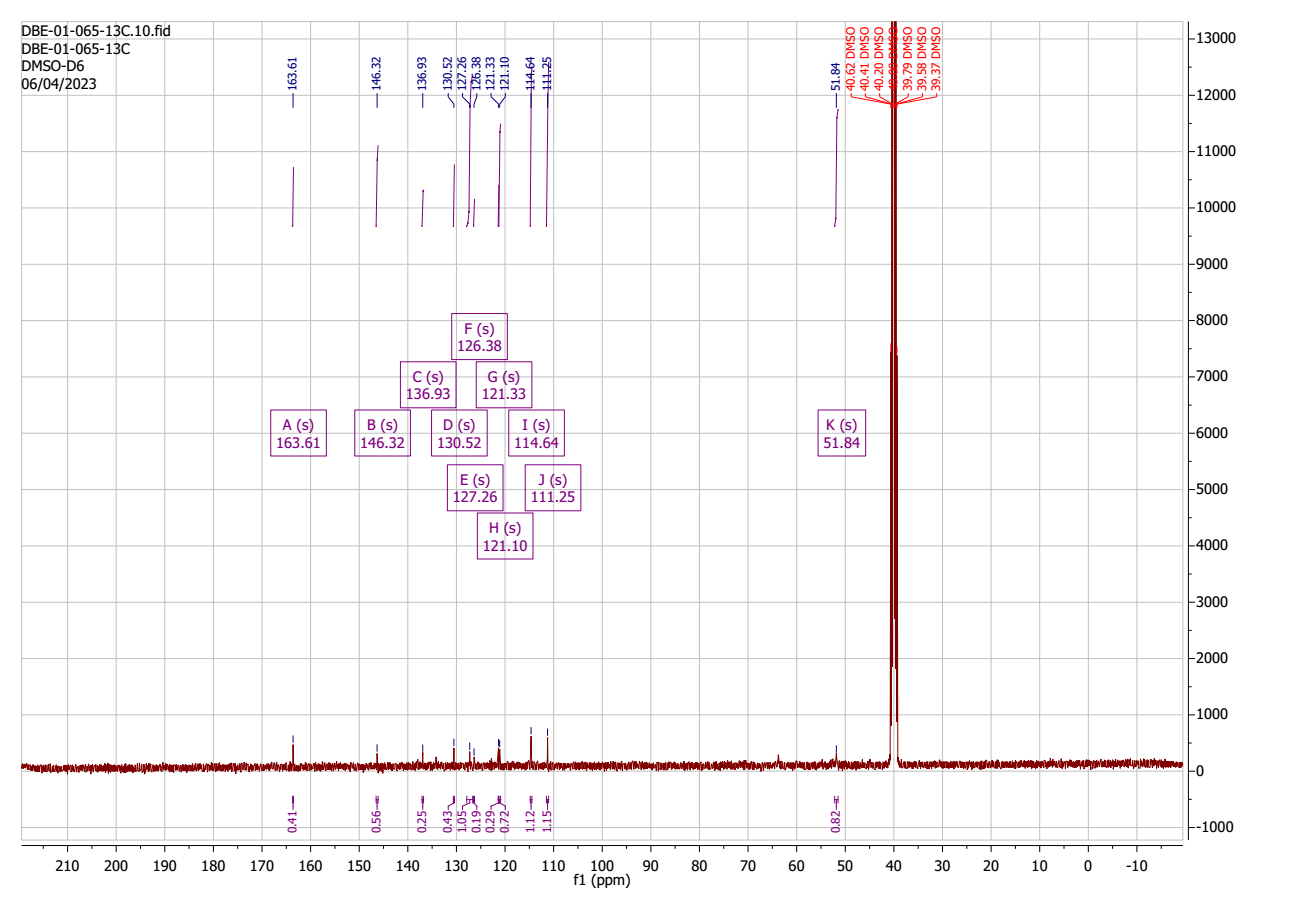


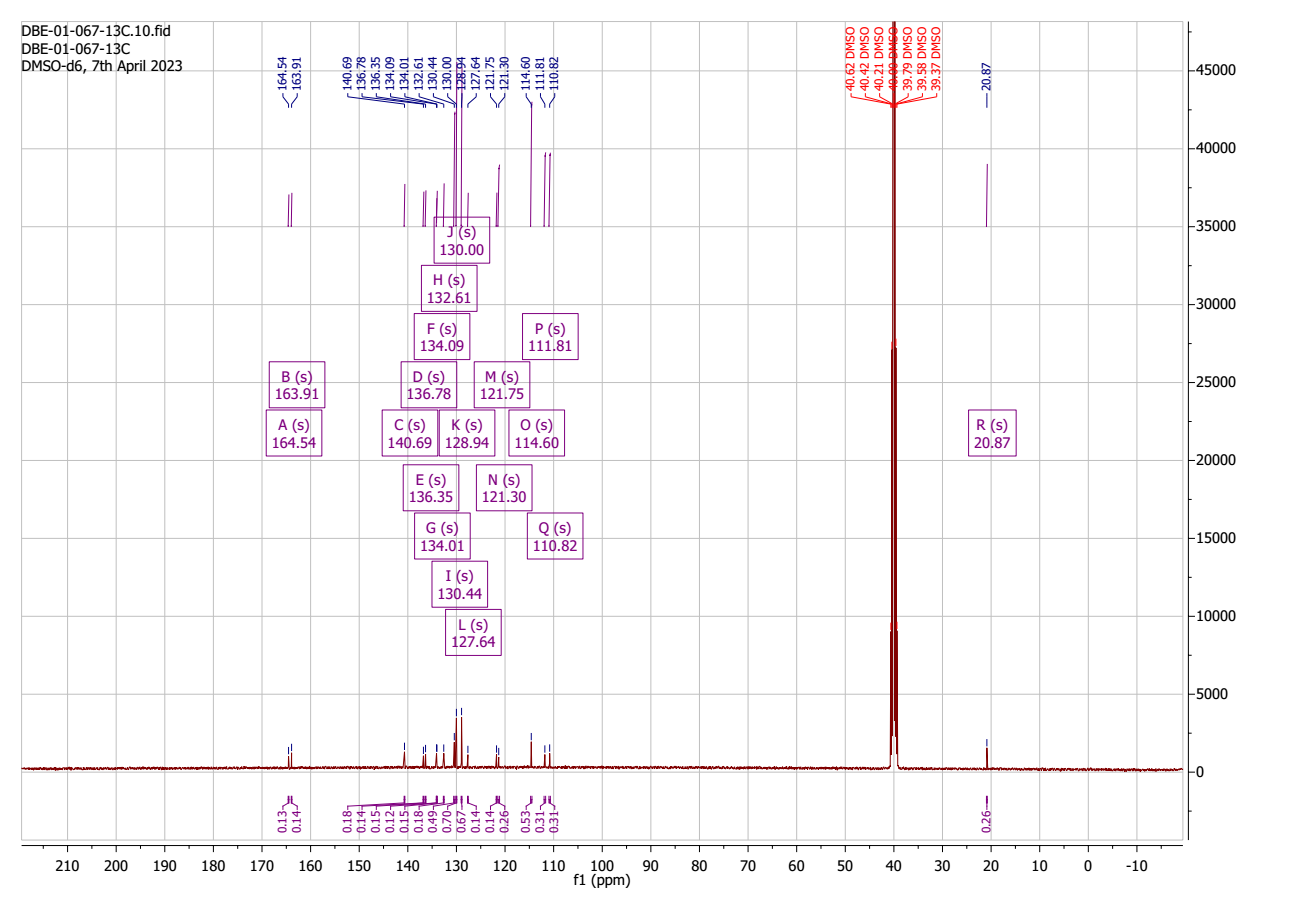


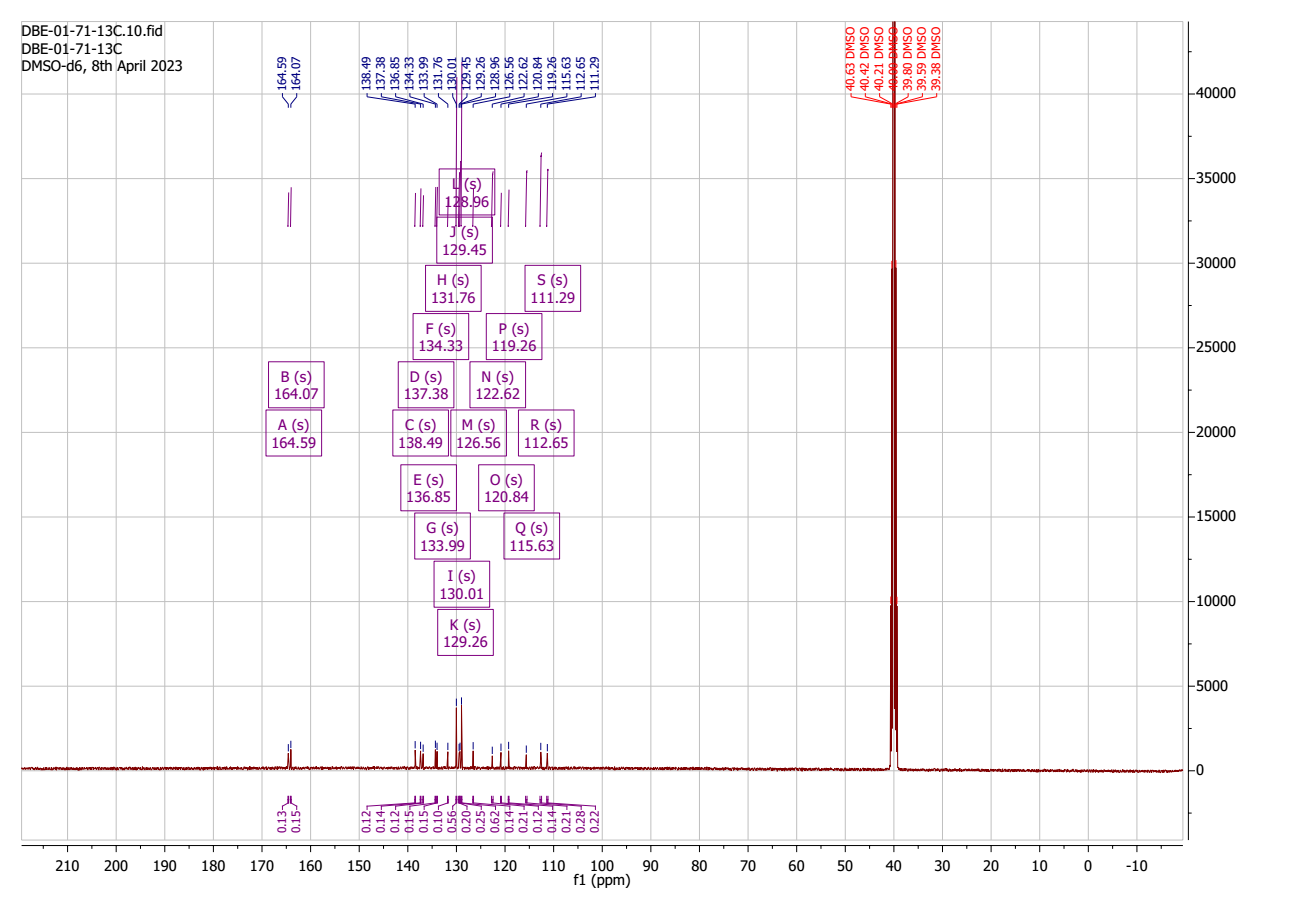


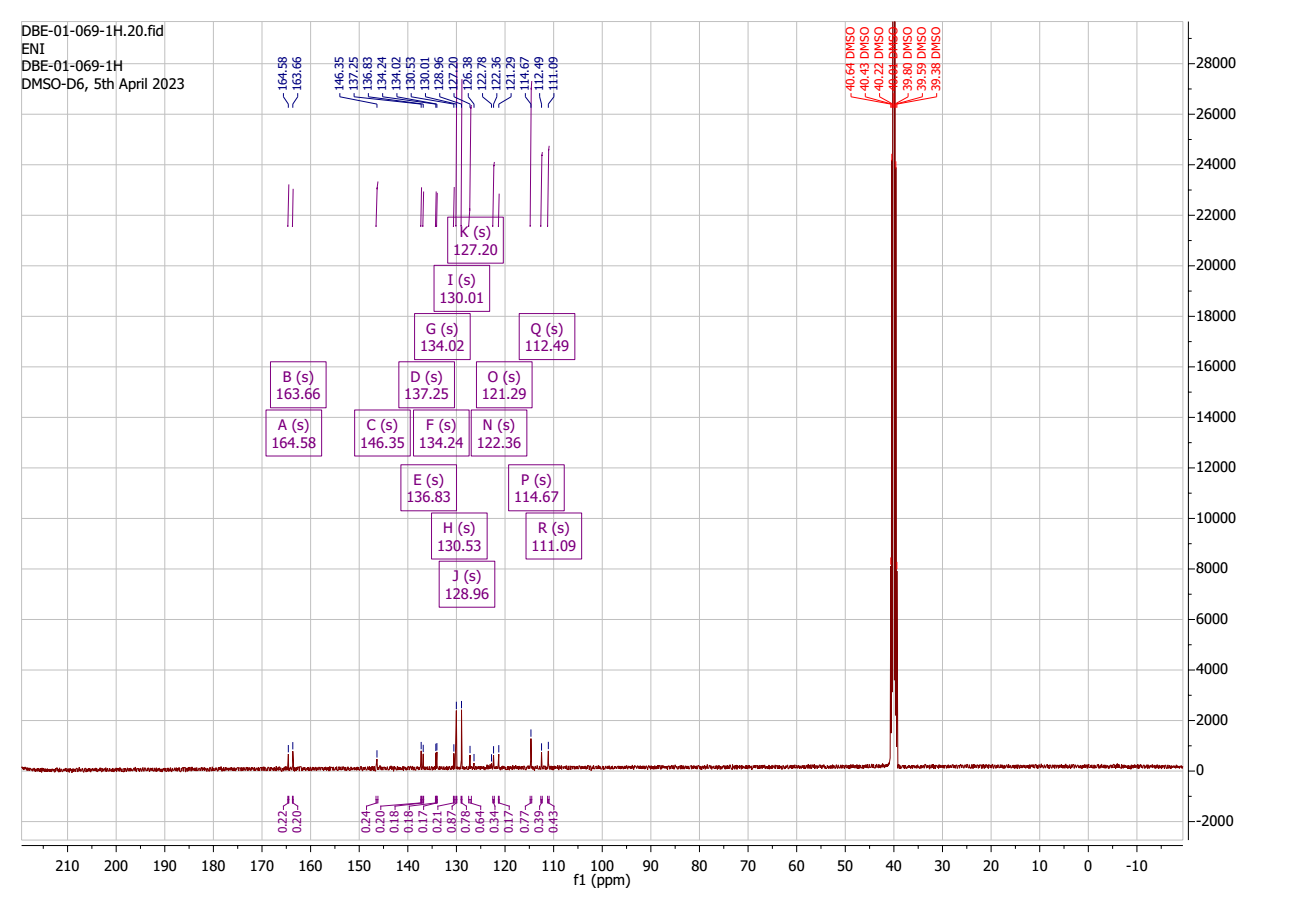


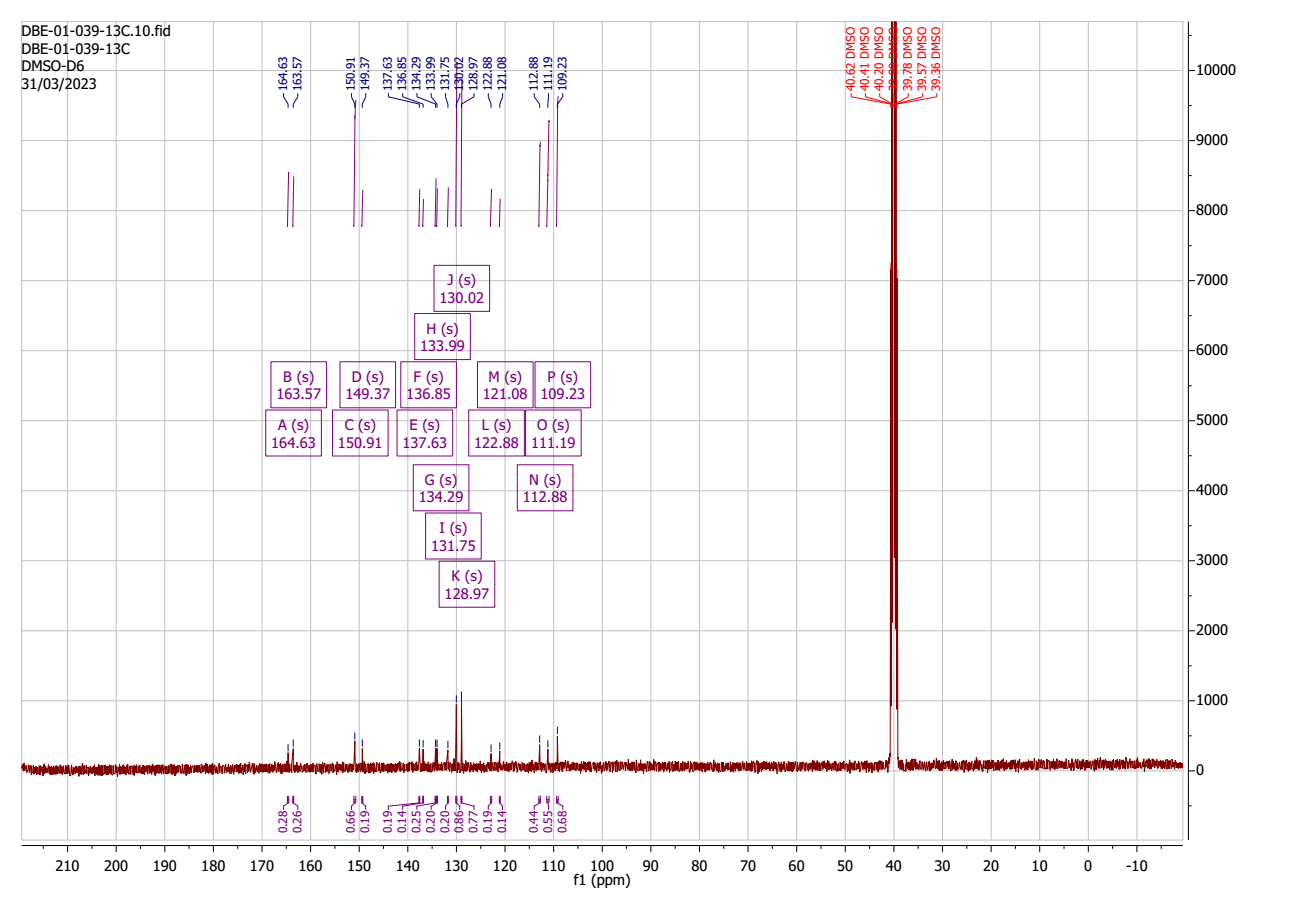


**LC-MS DATA**


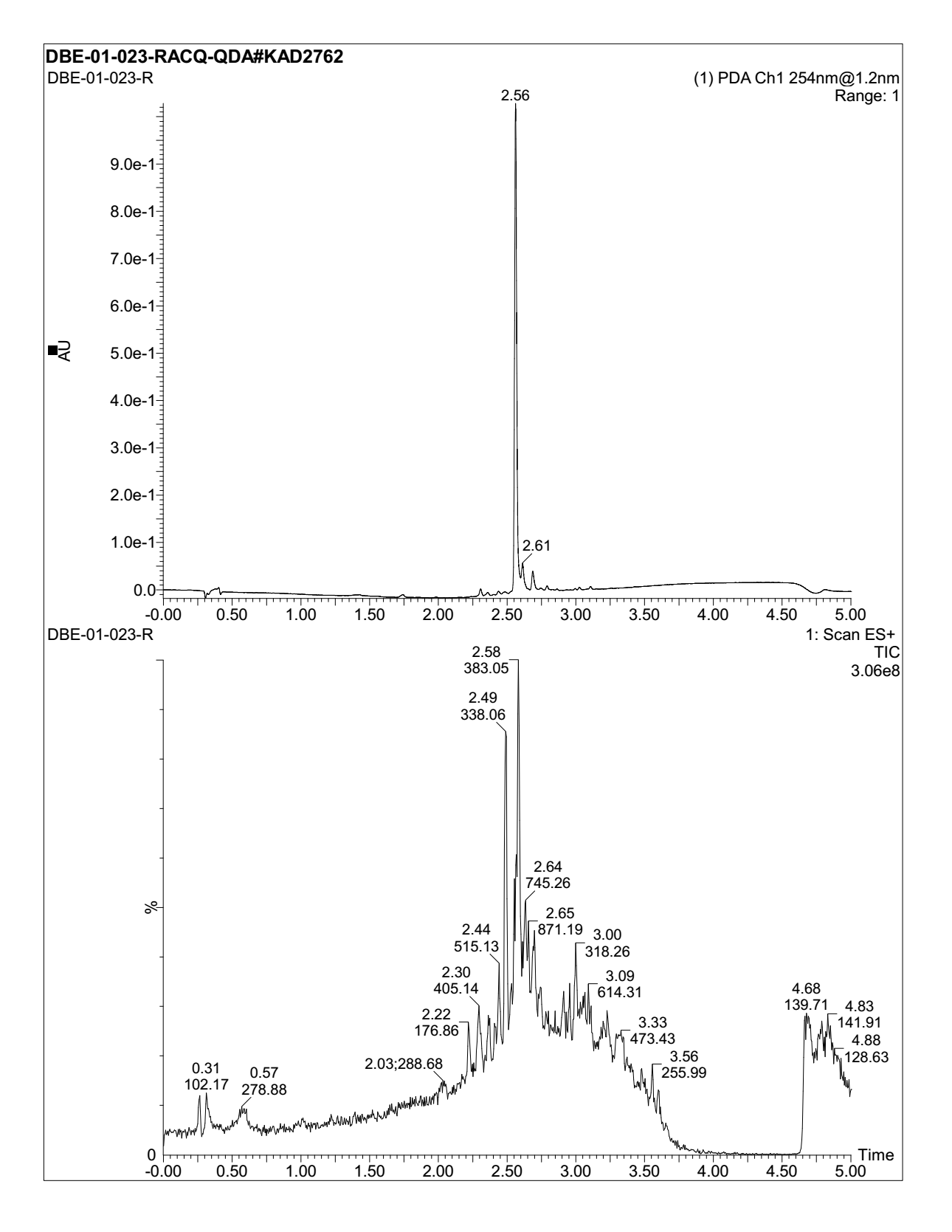


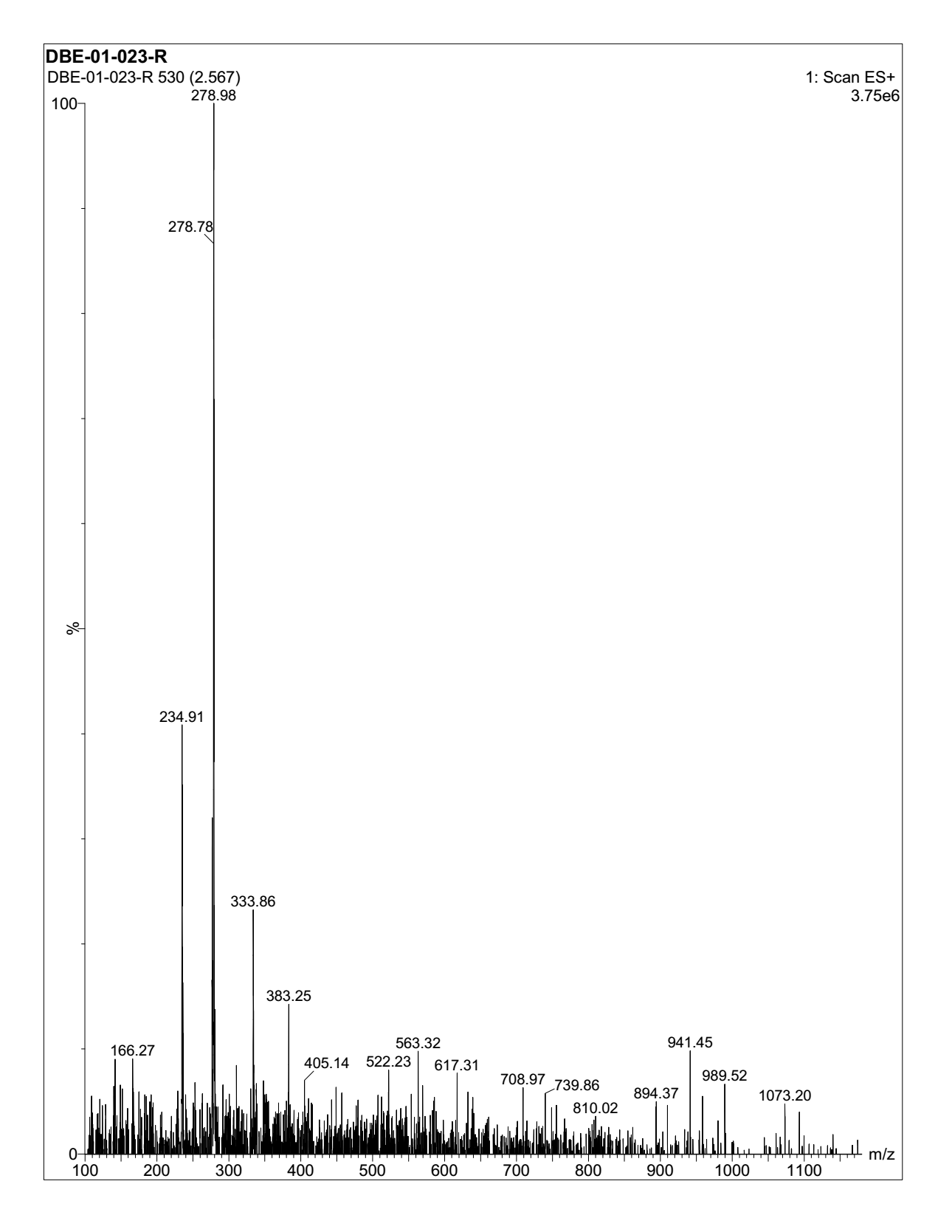


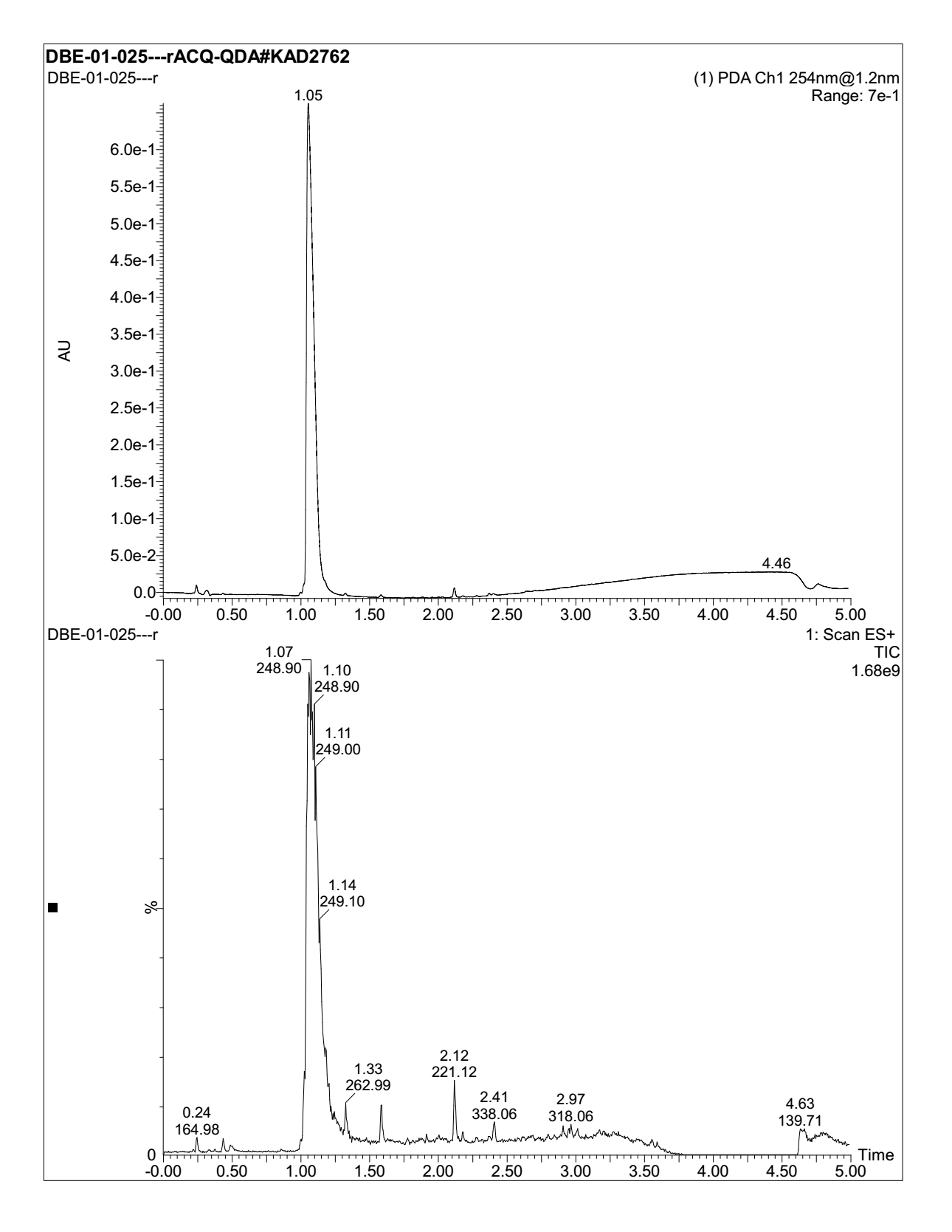


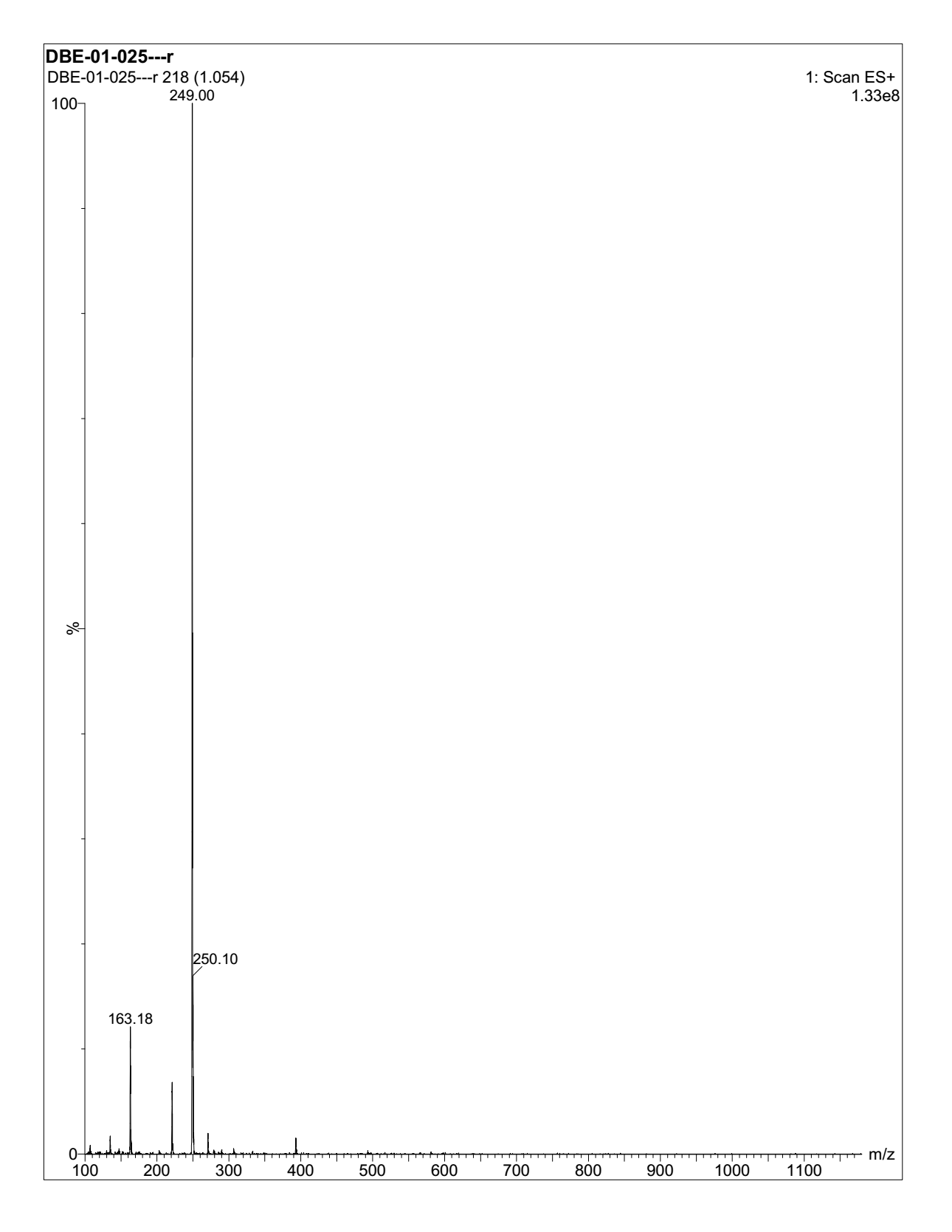


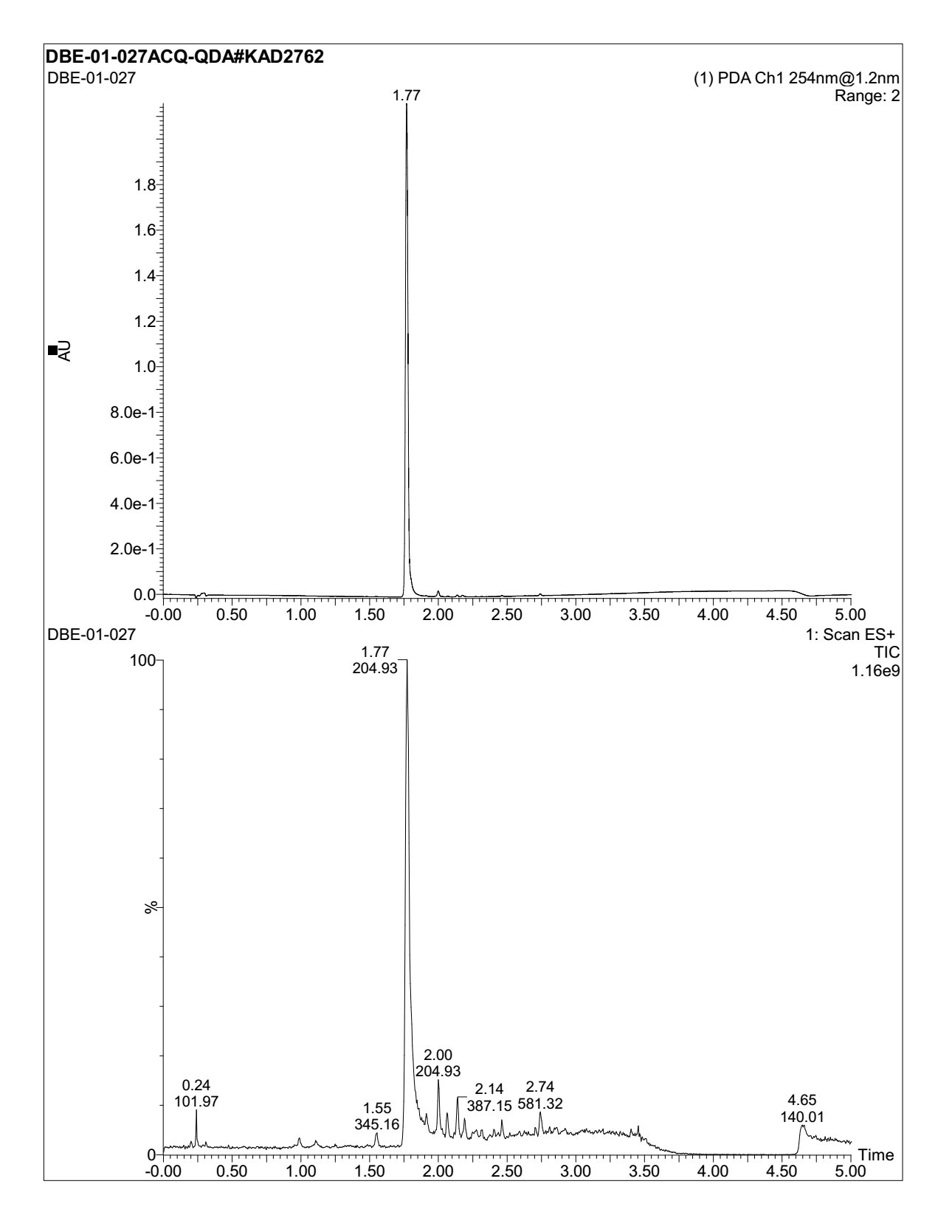


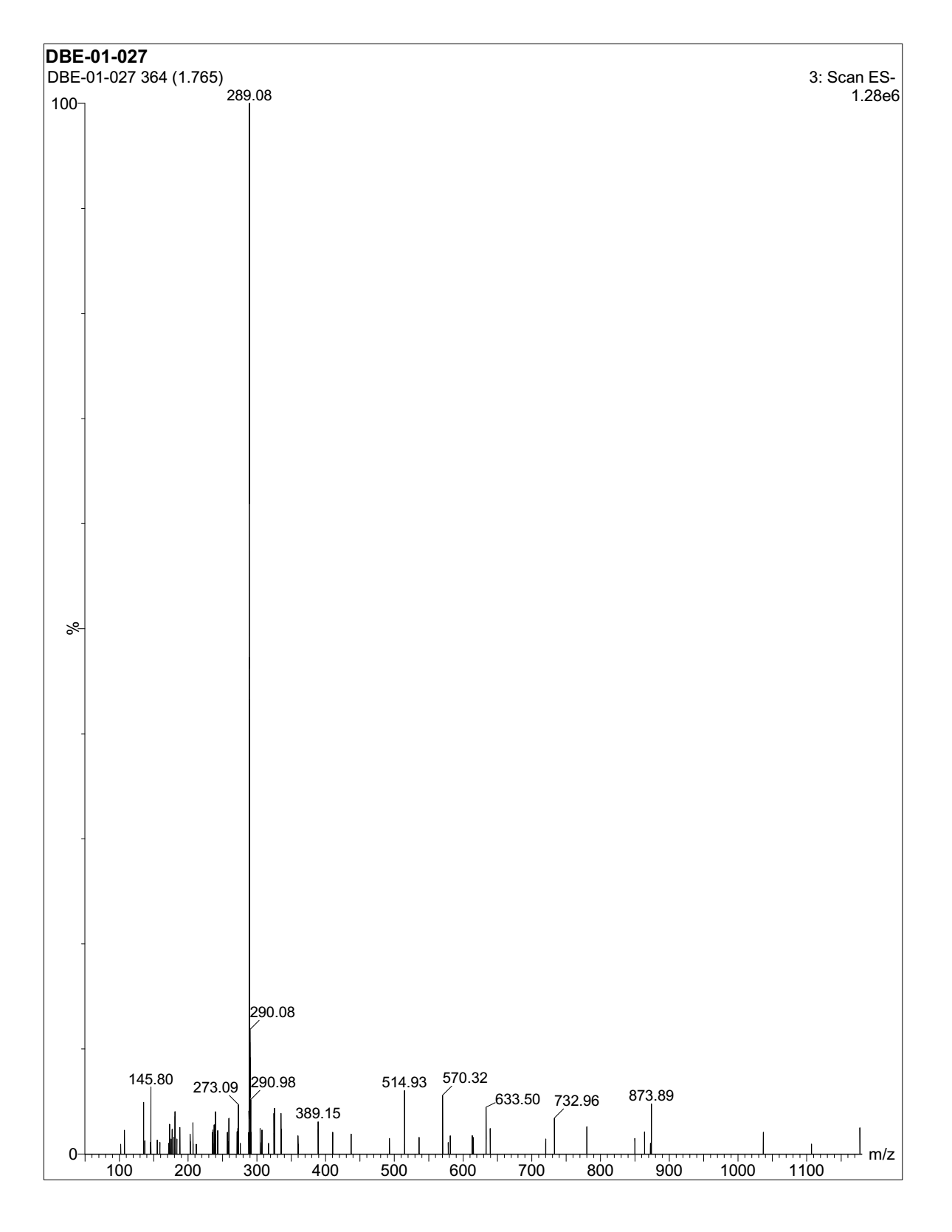


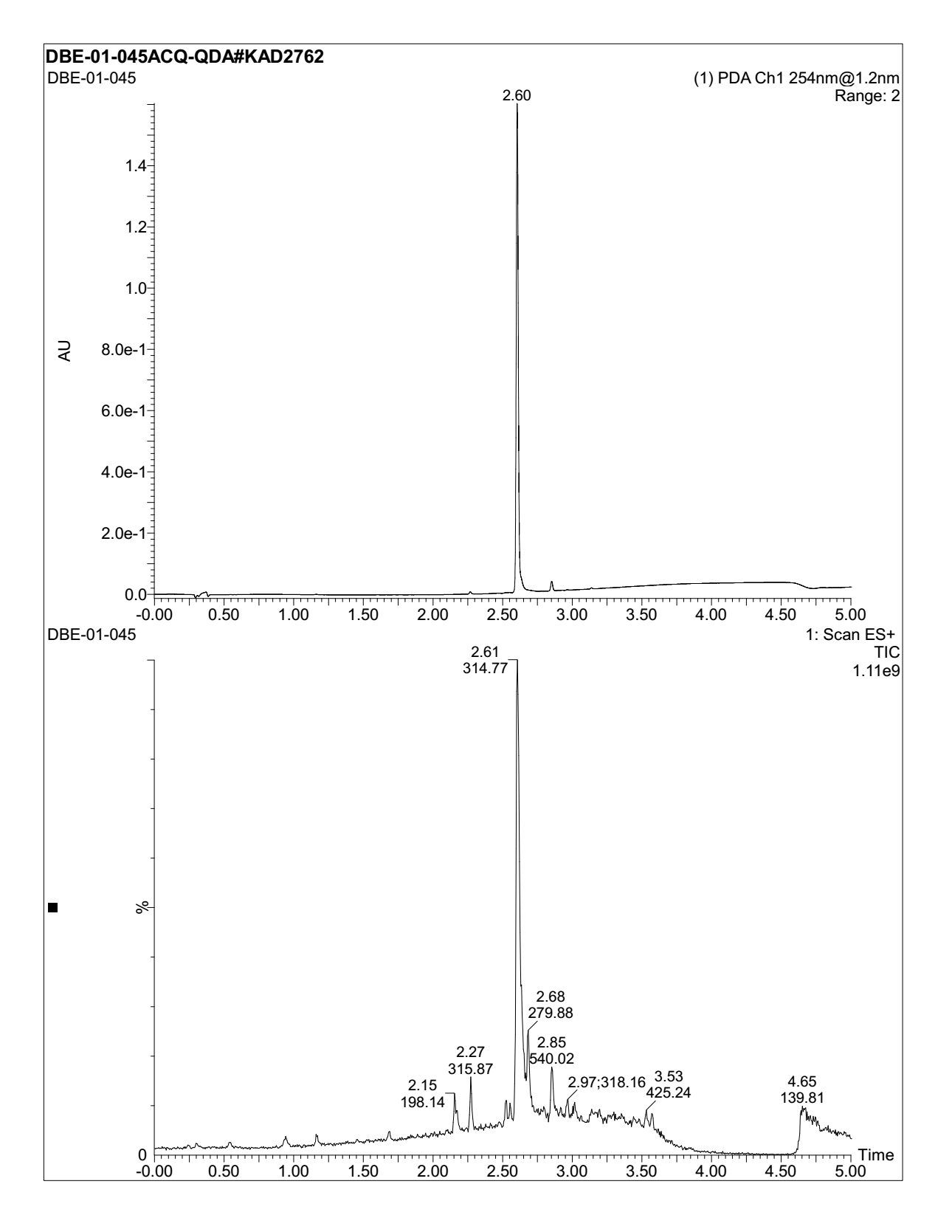


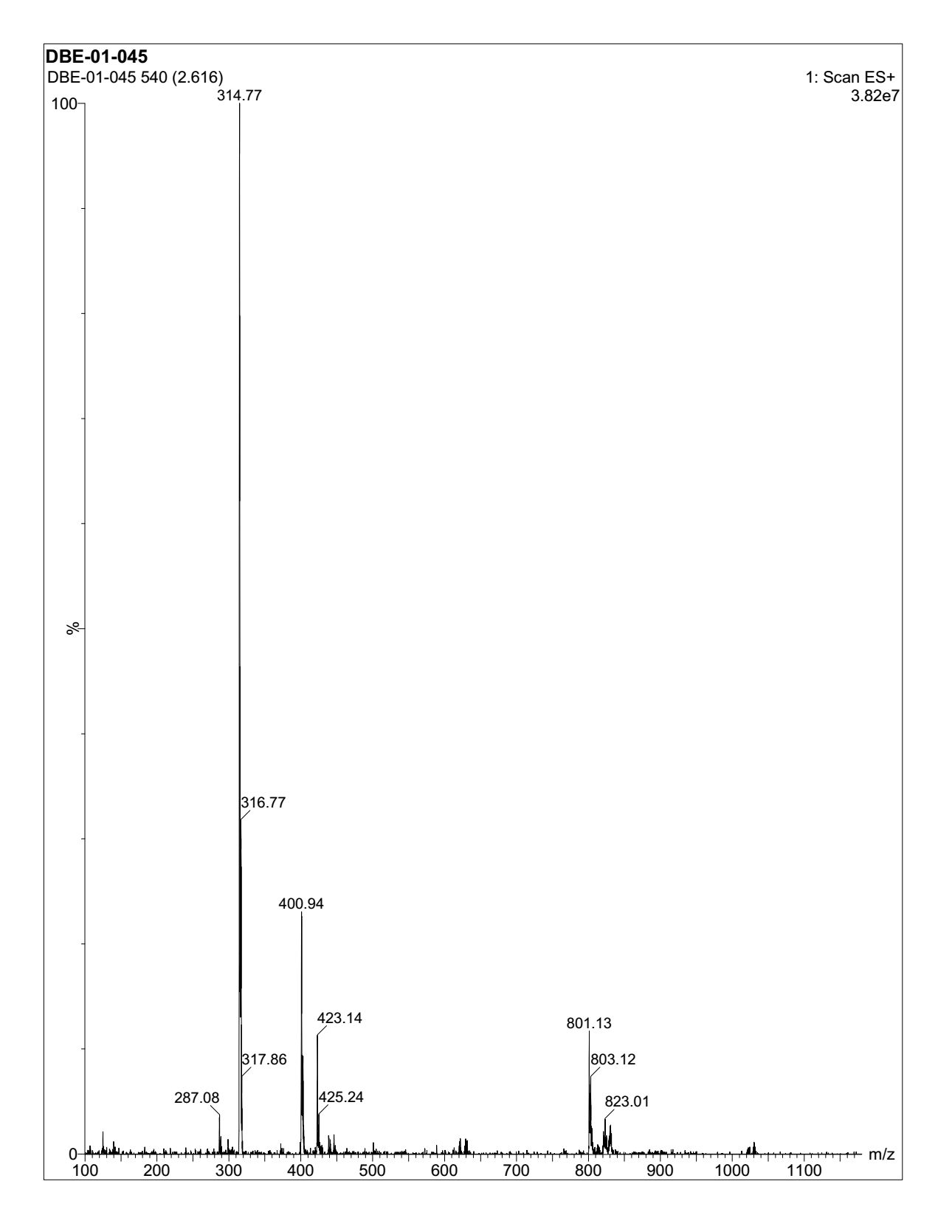


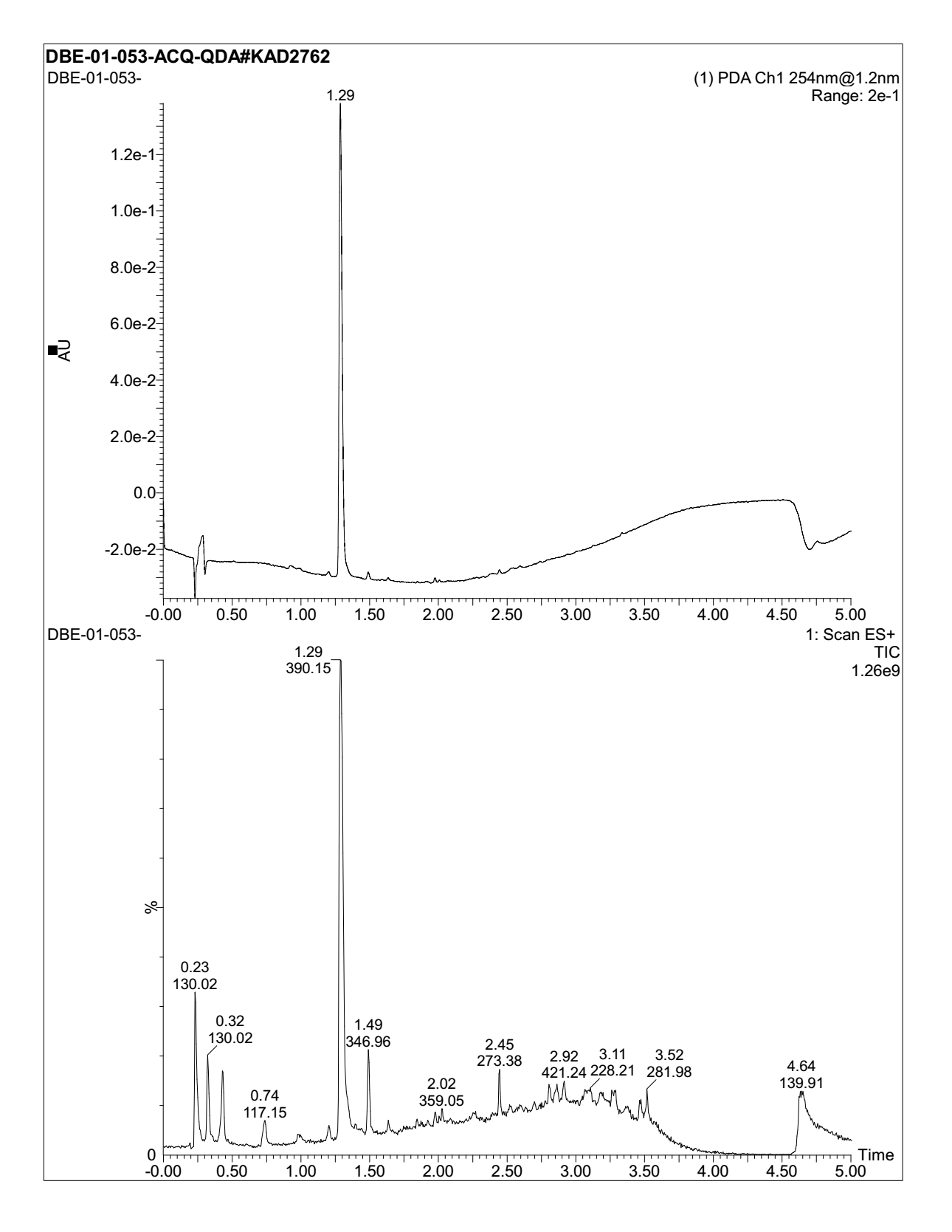


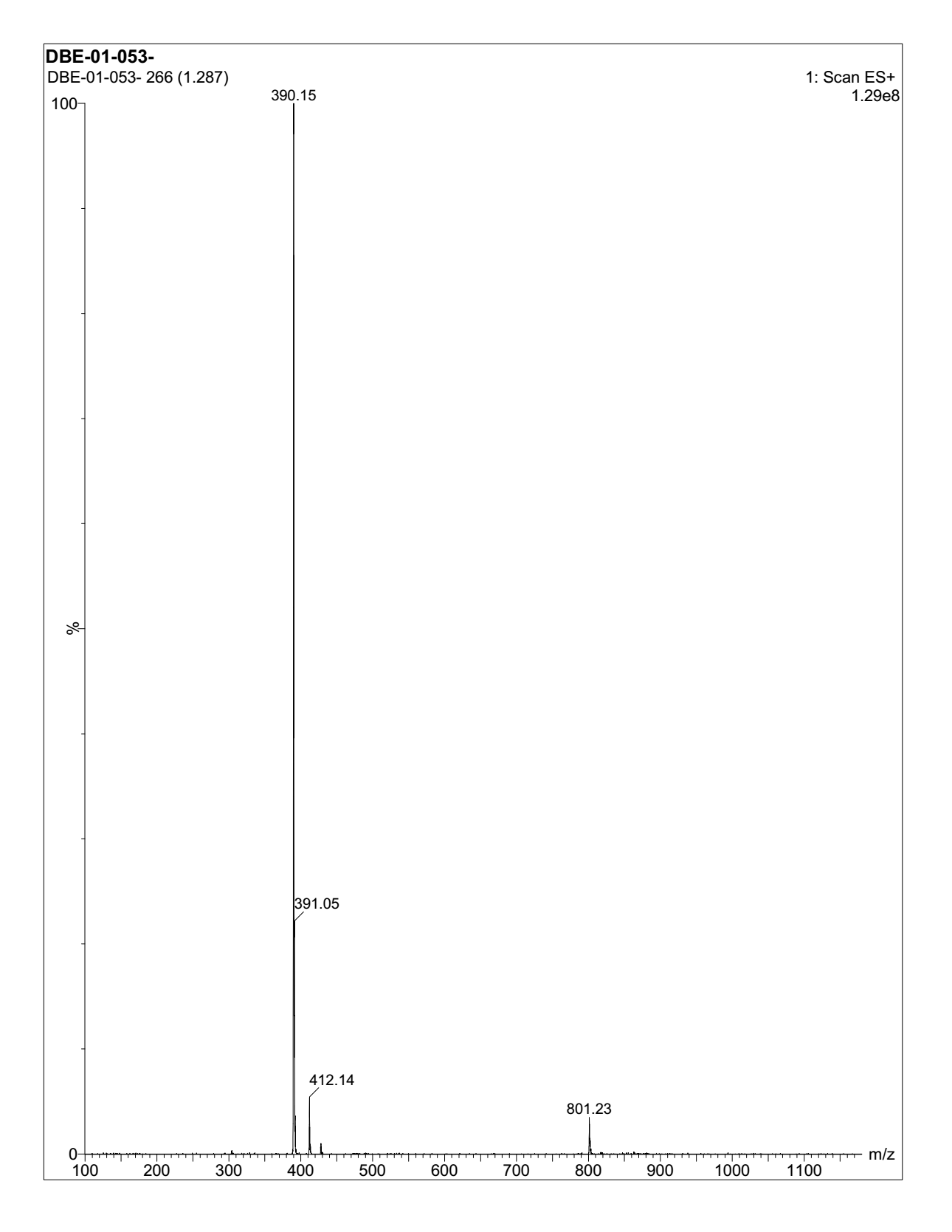


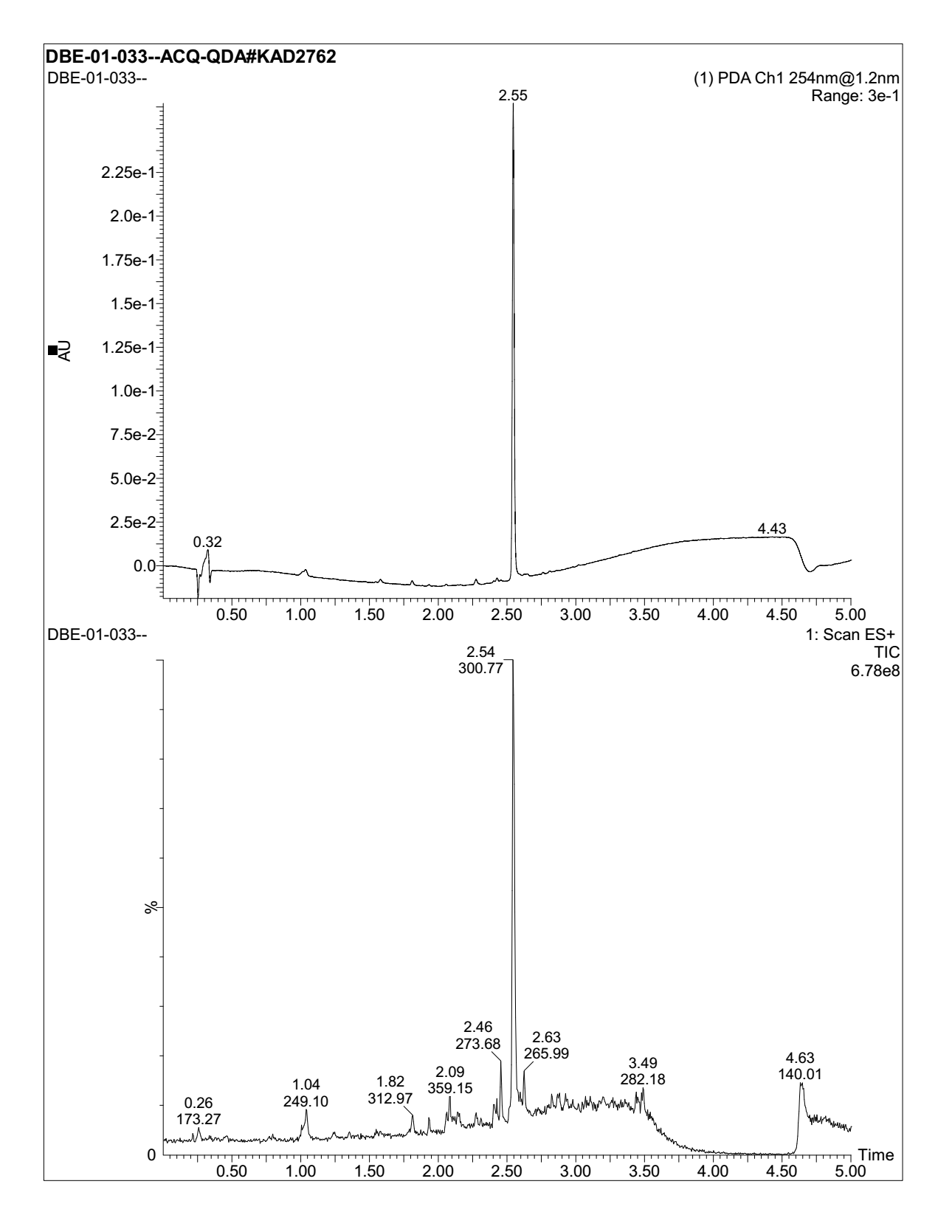


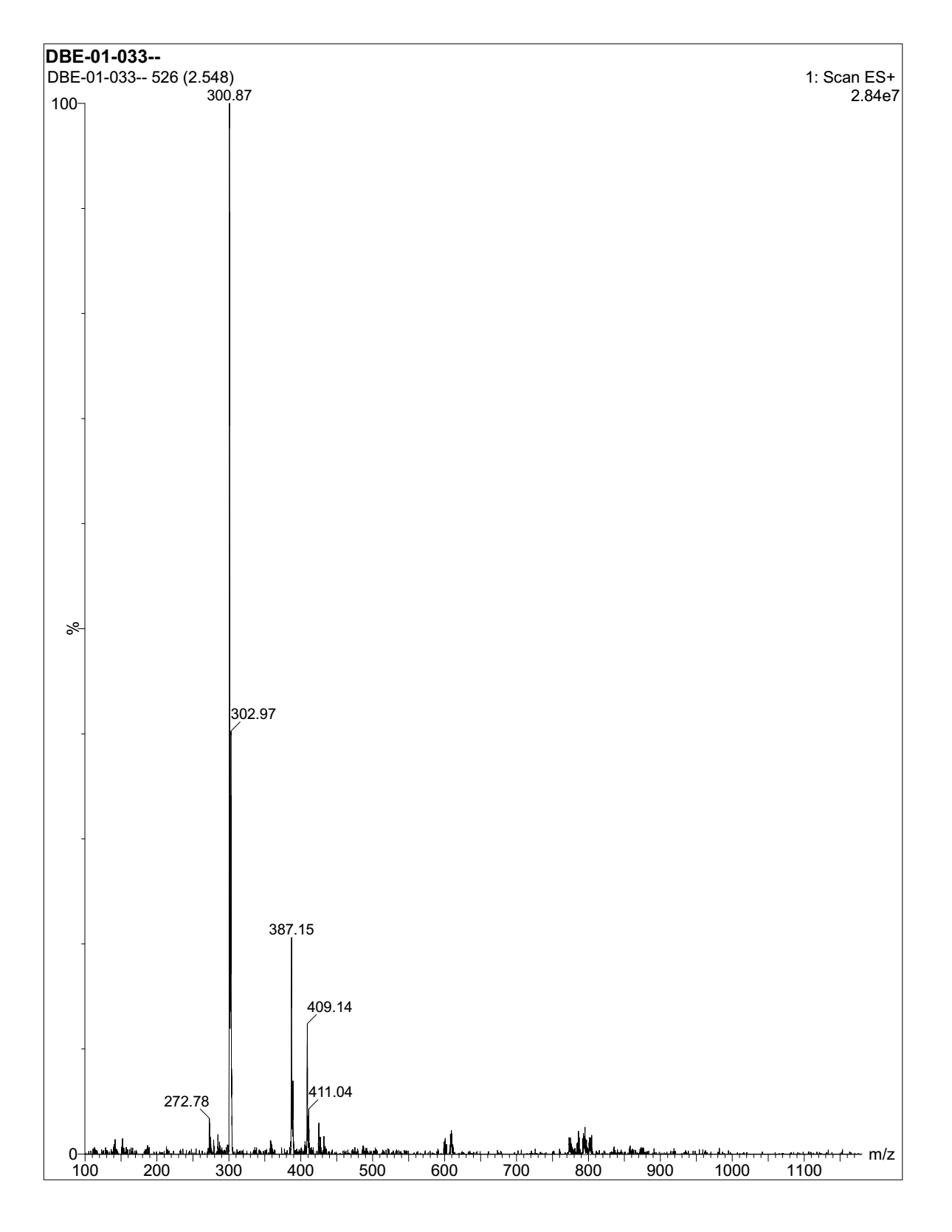


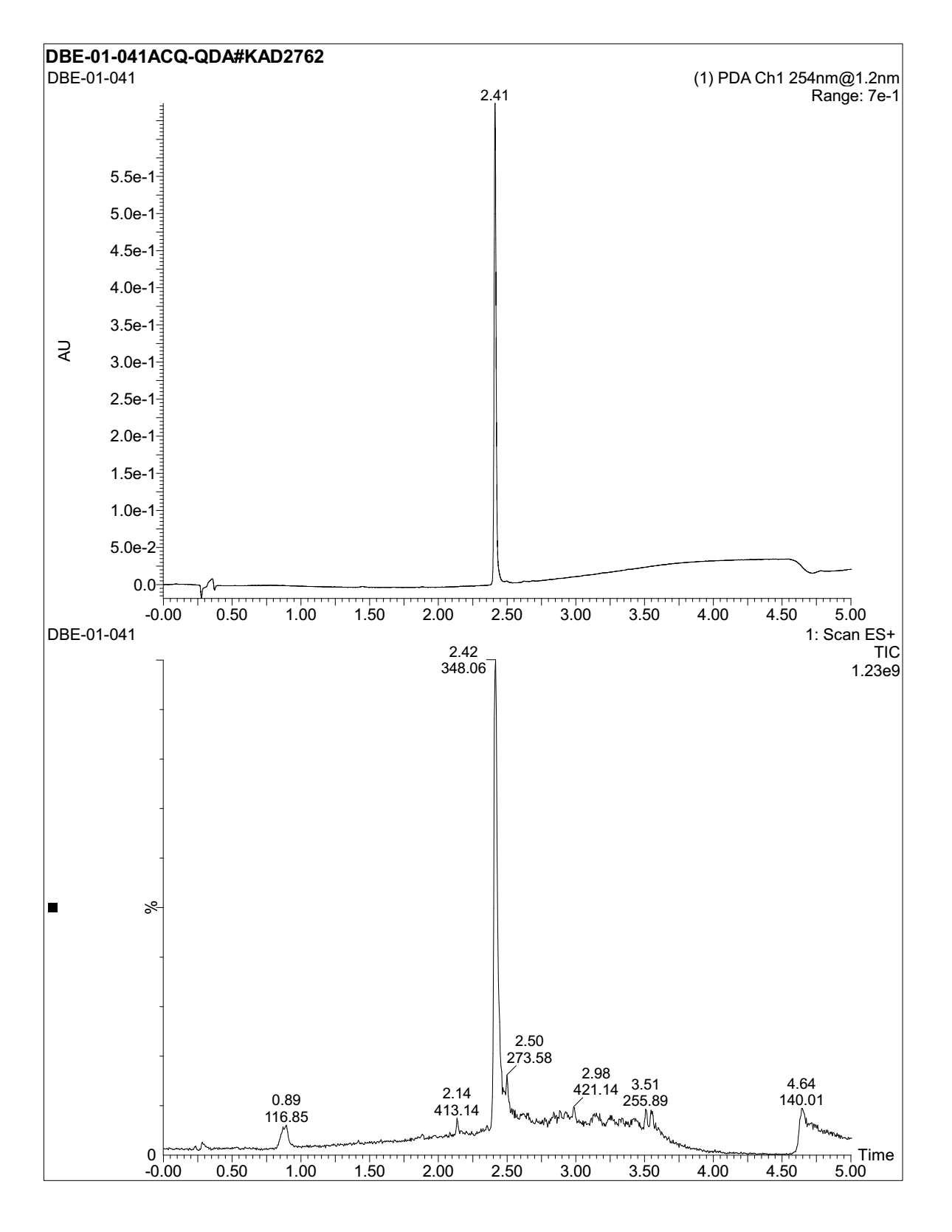


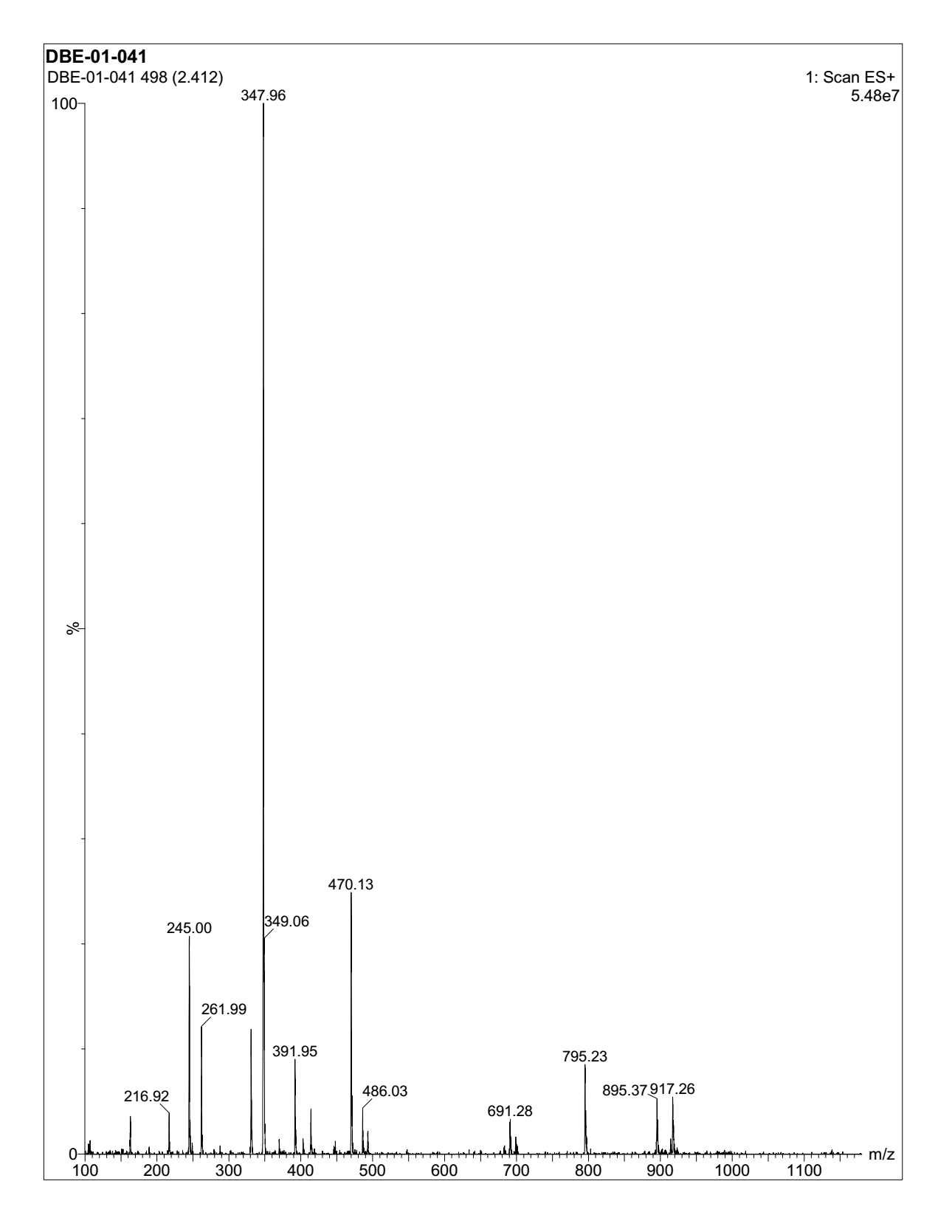


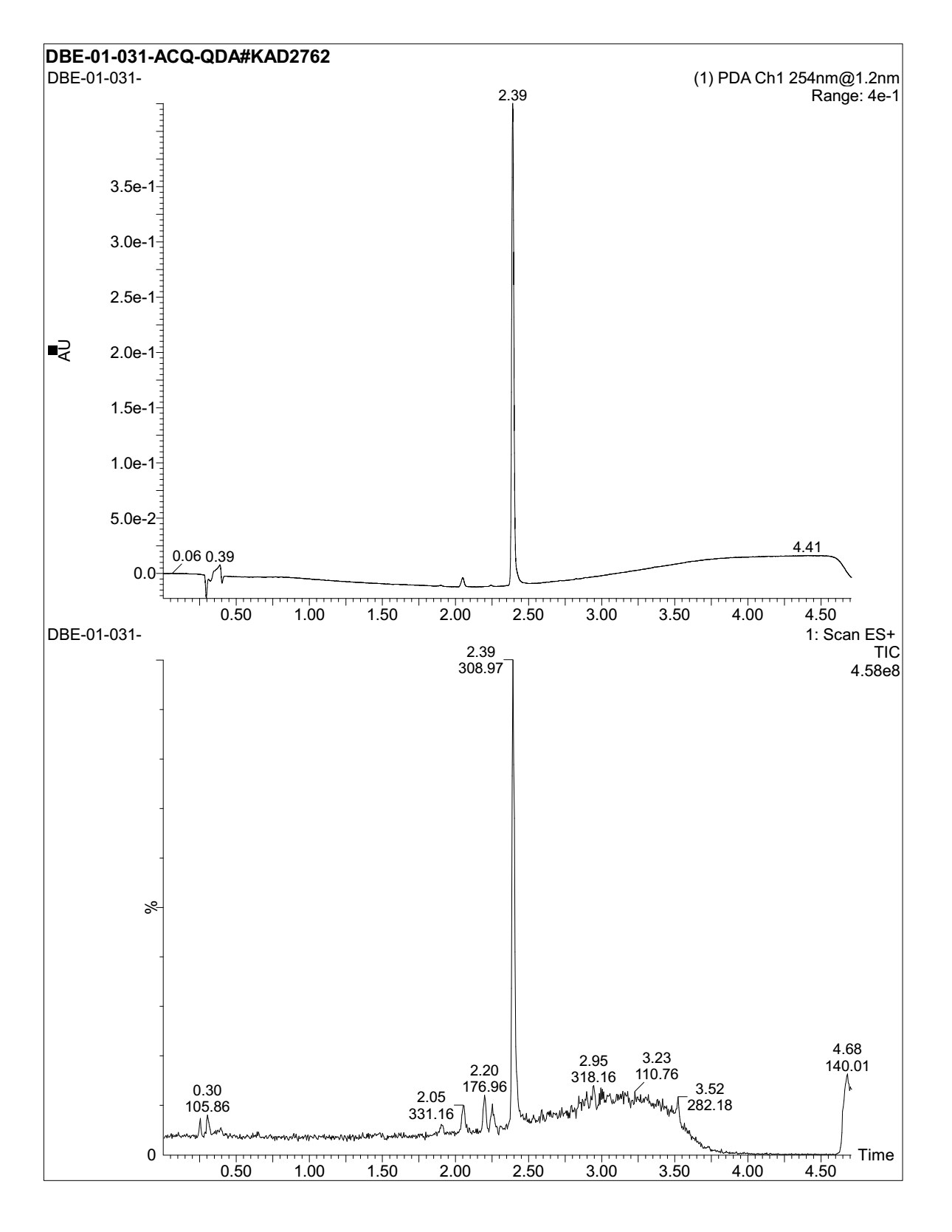


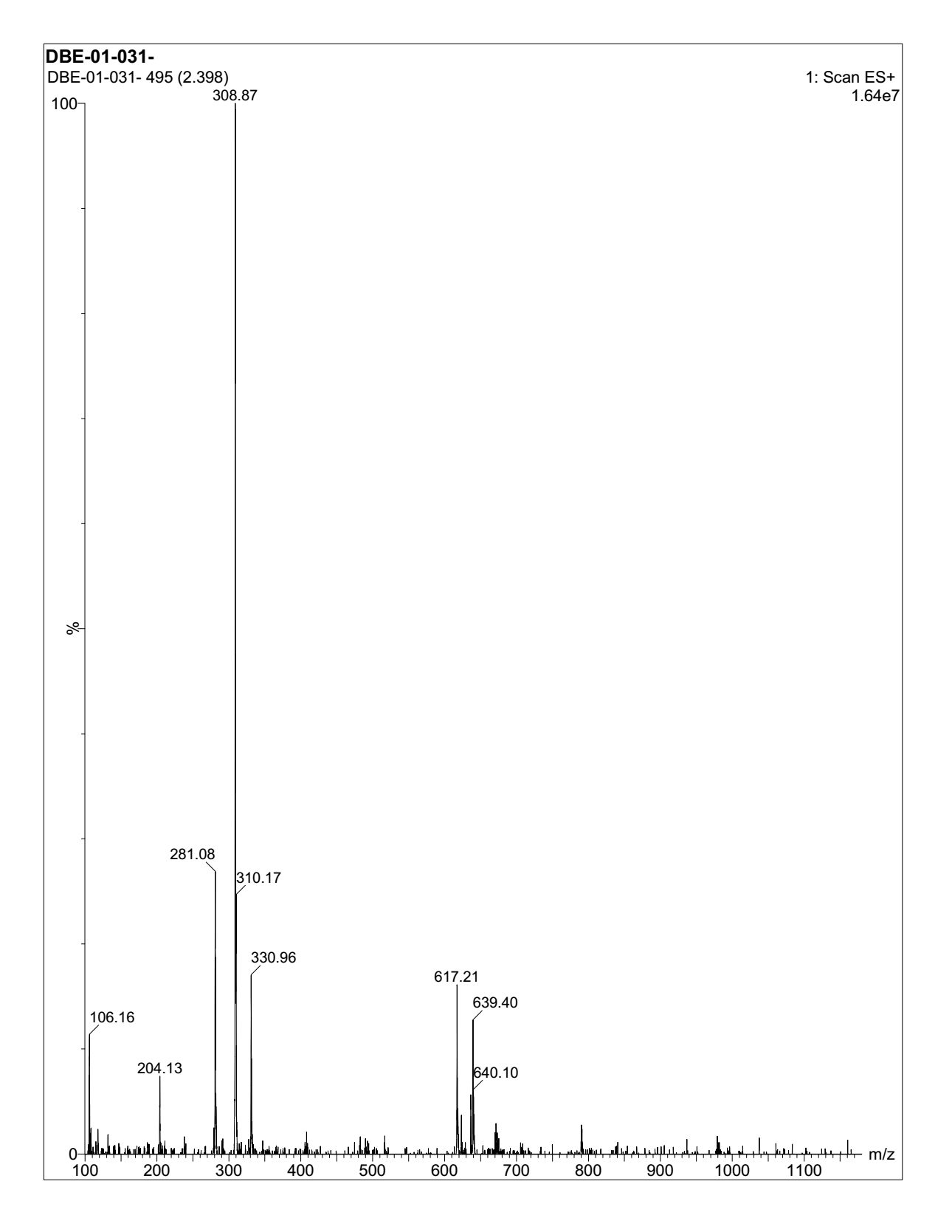


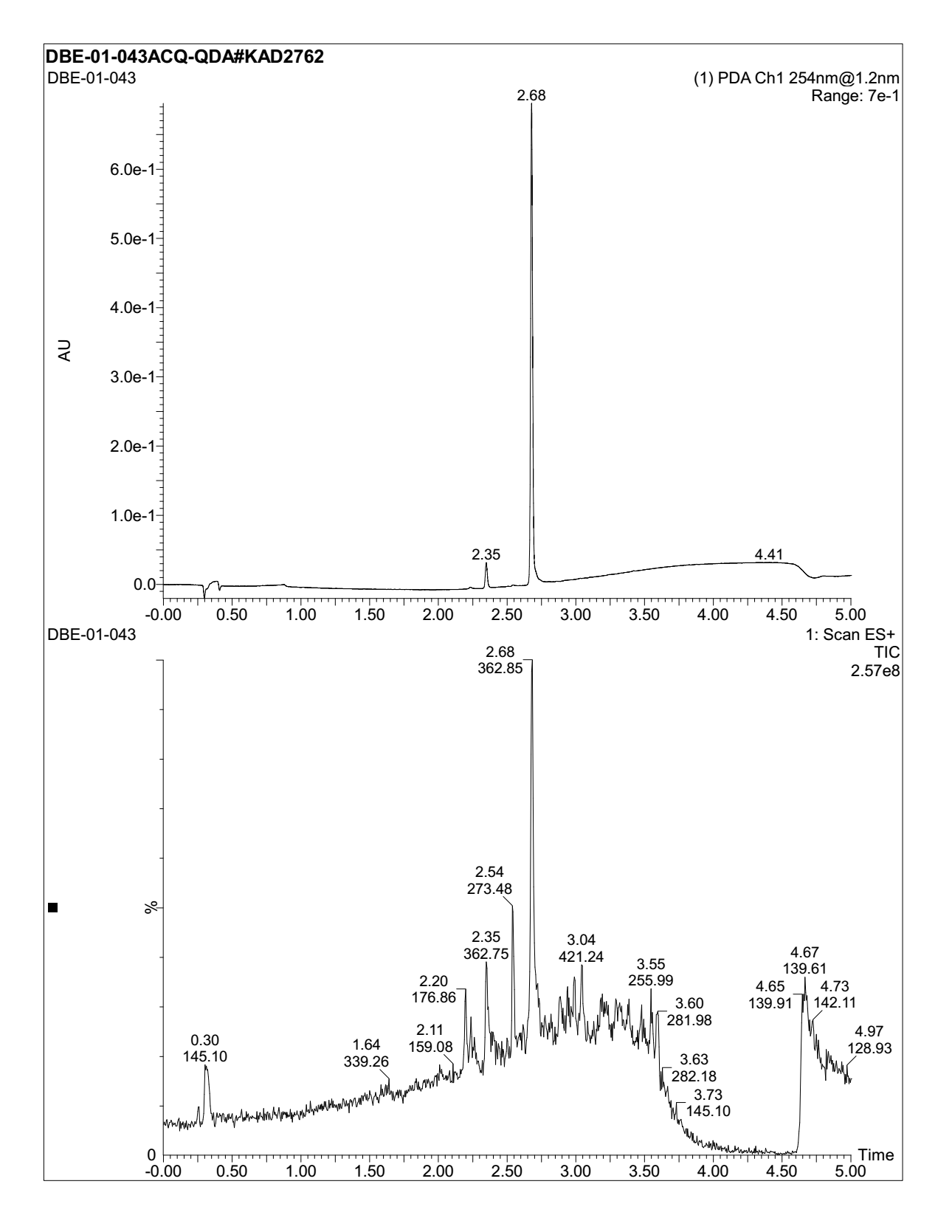


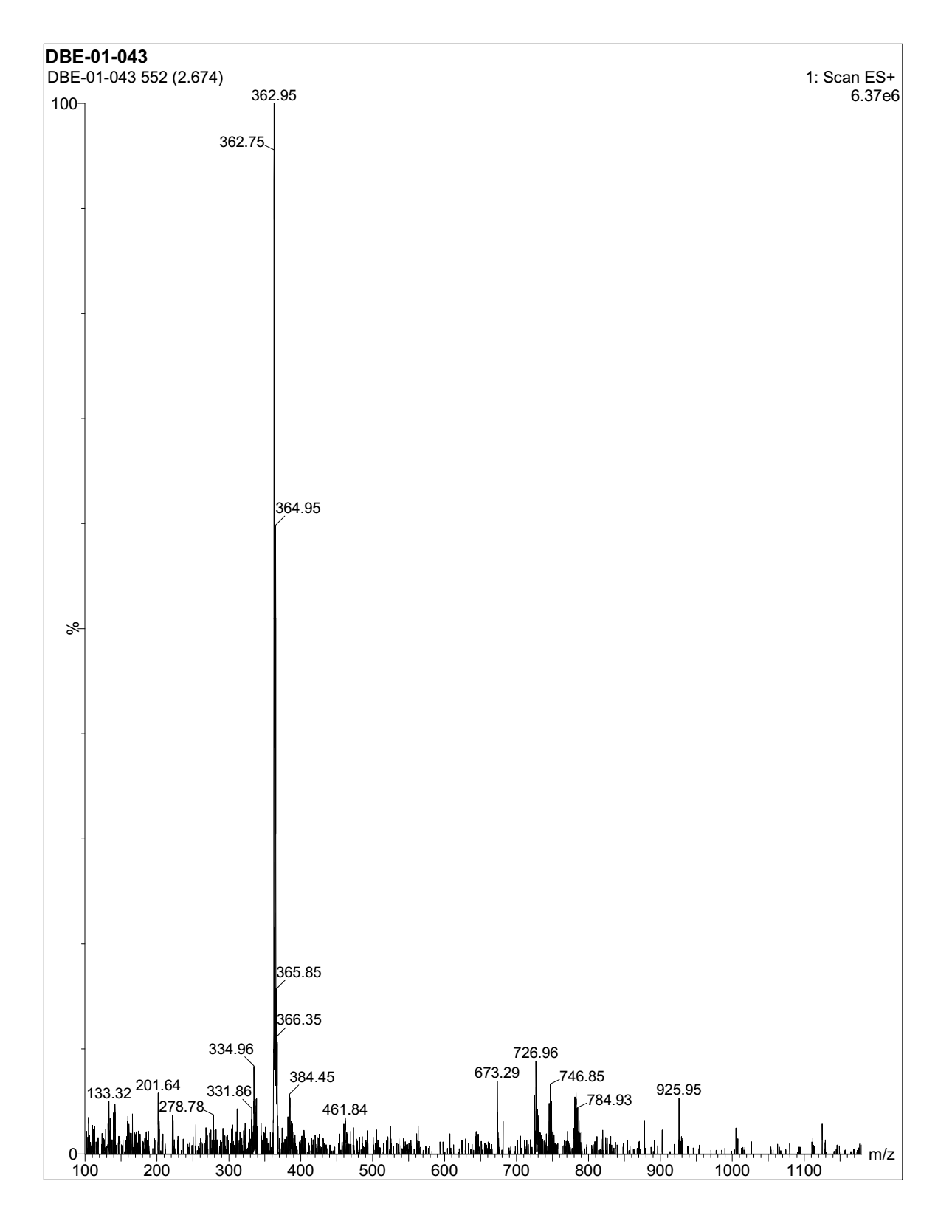


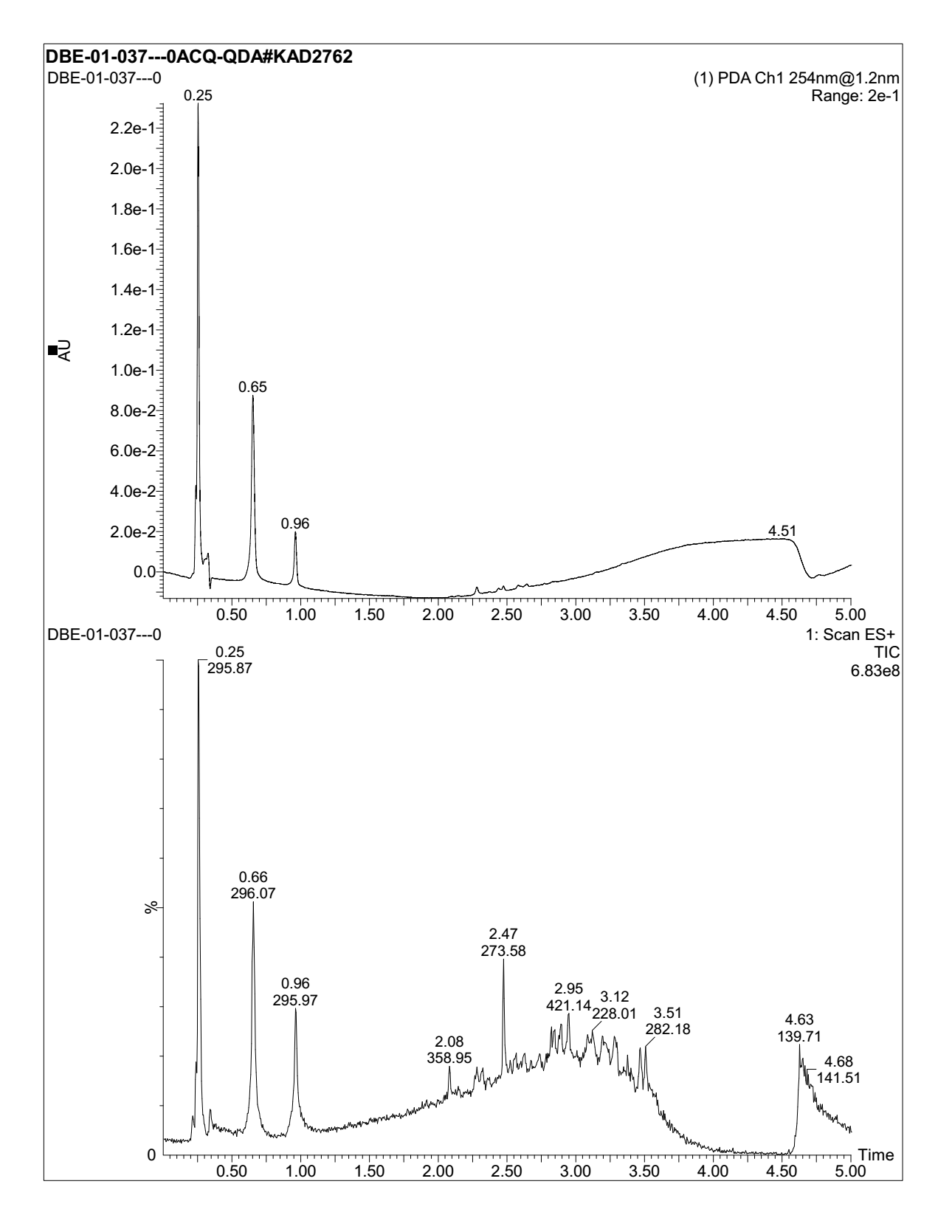


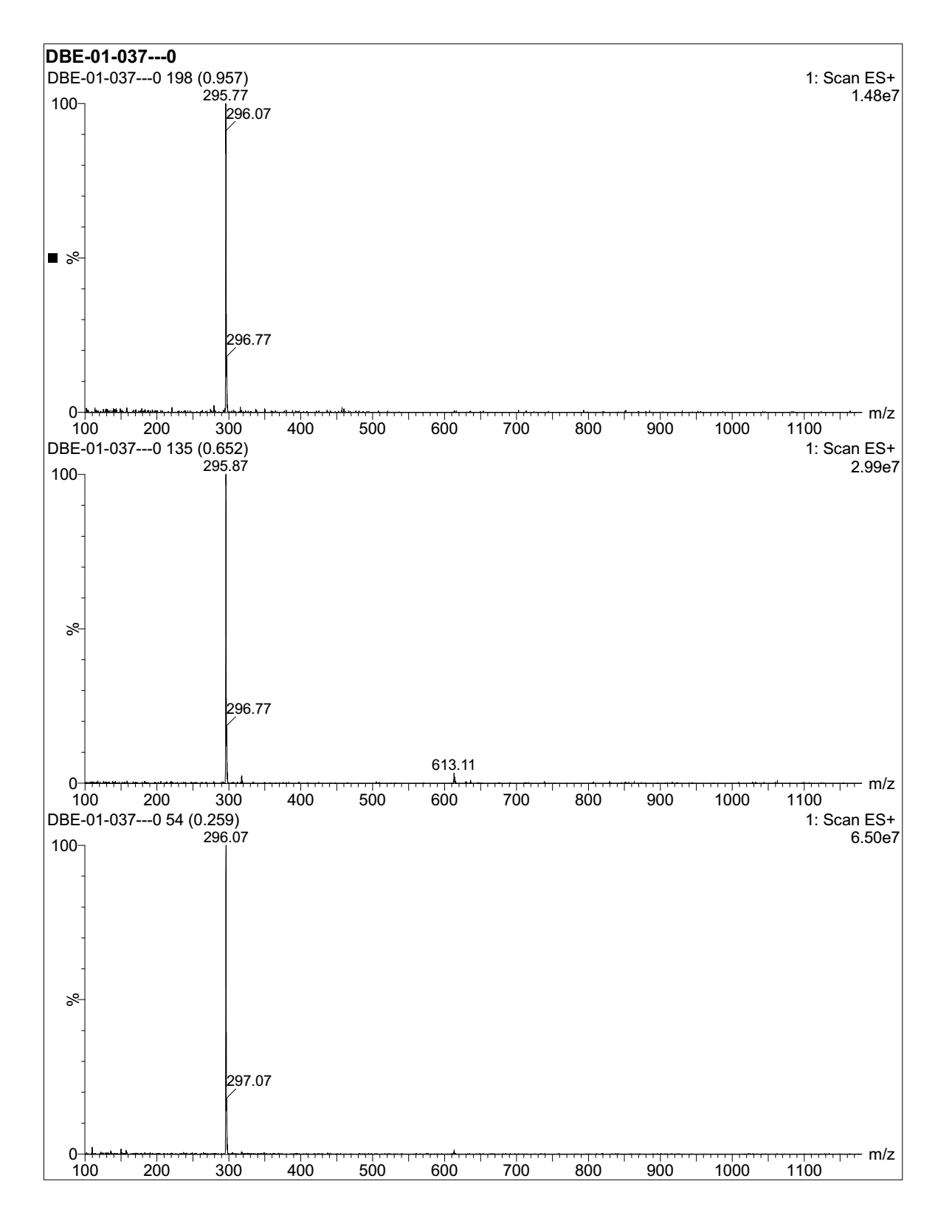


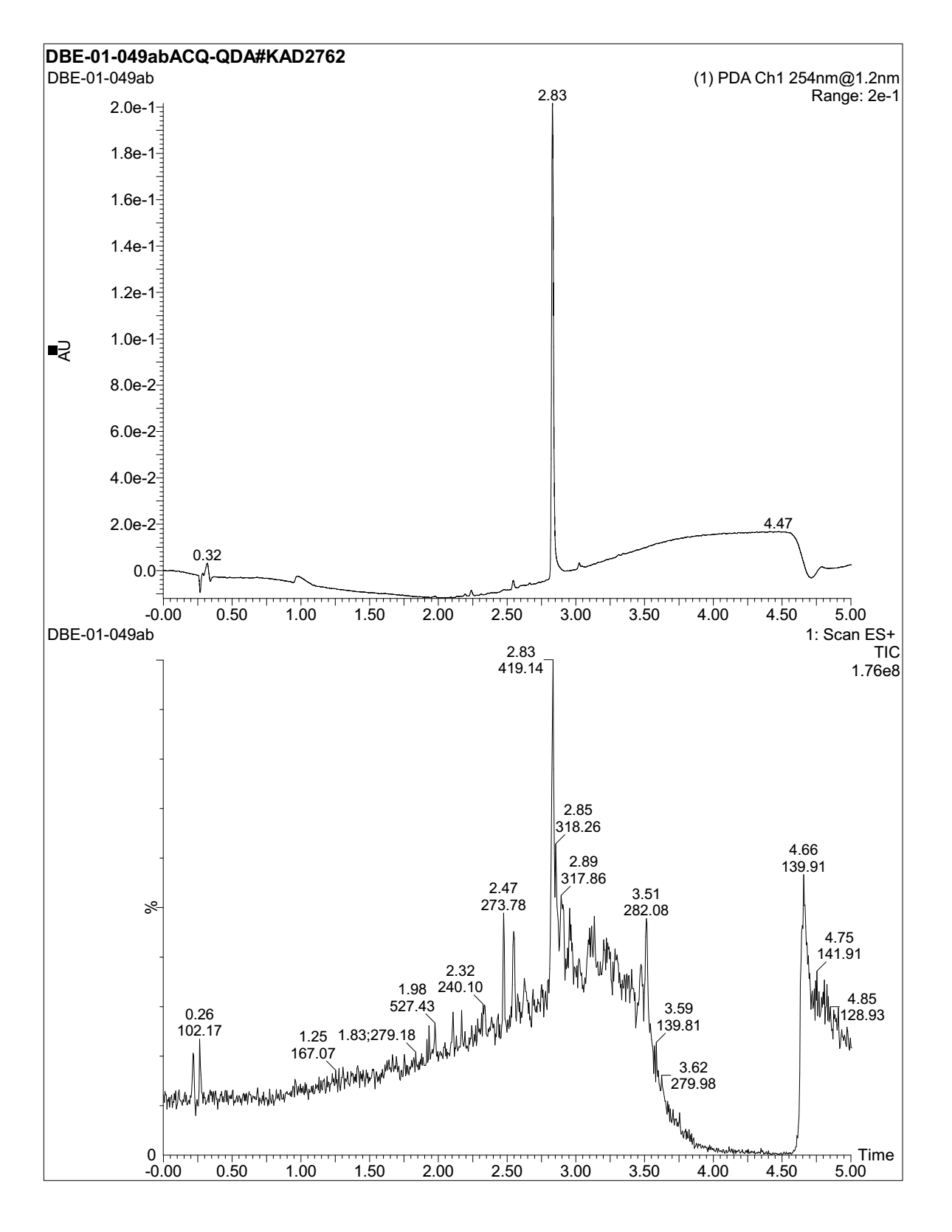


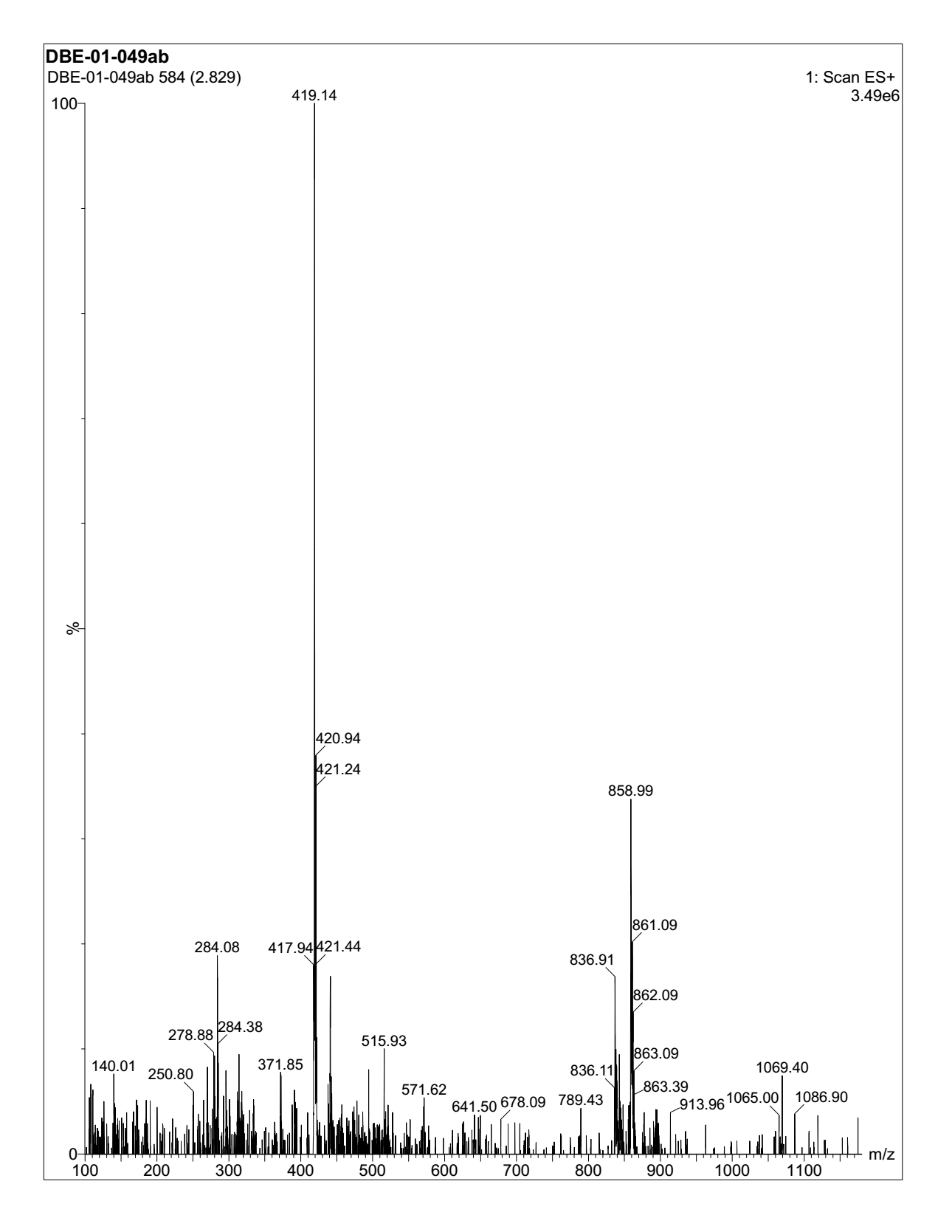


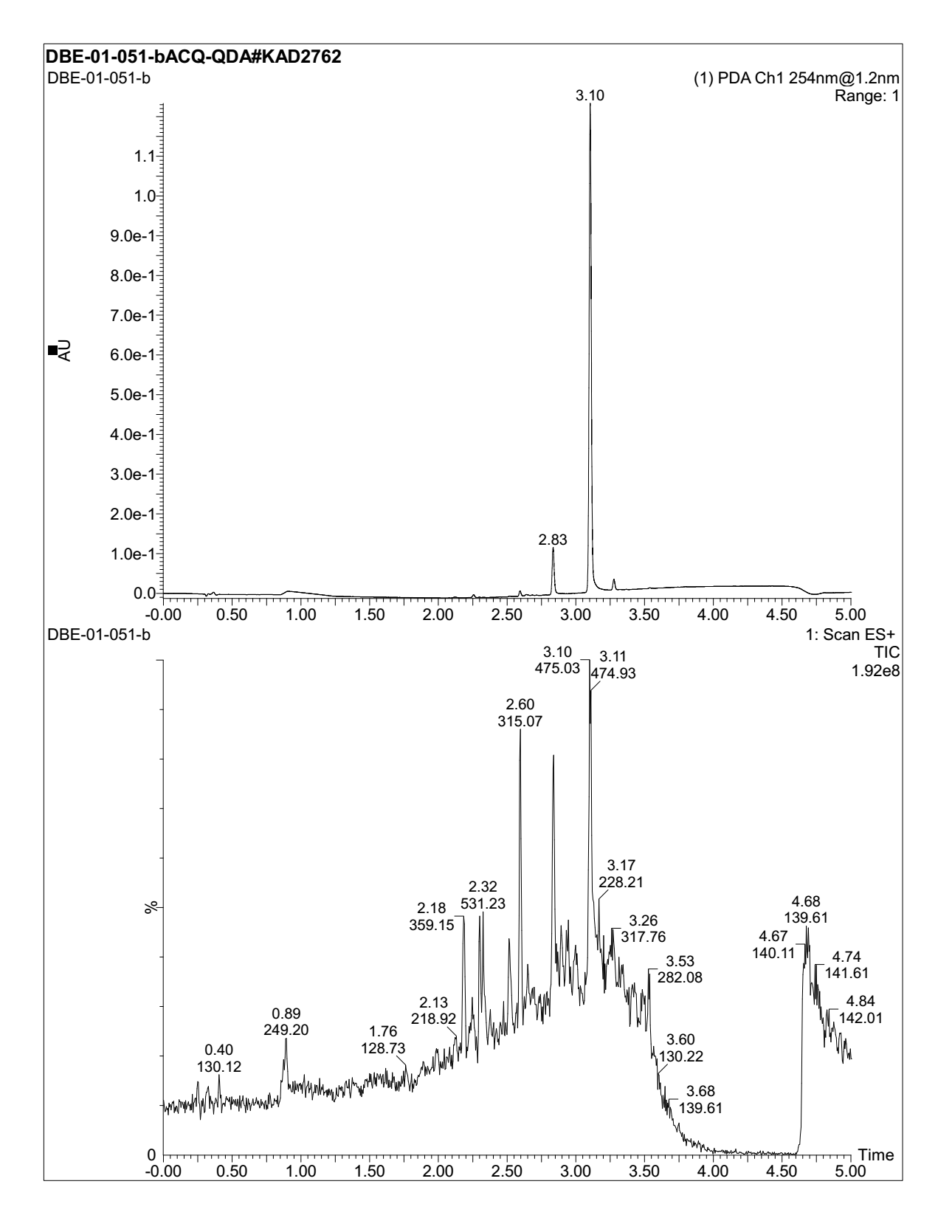


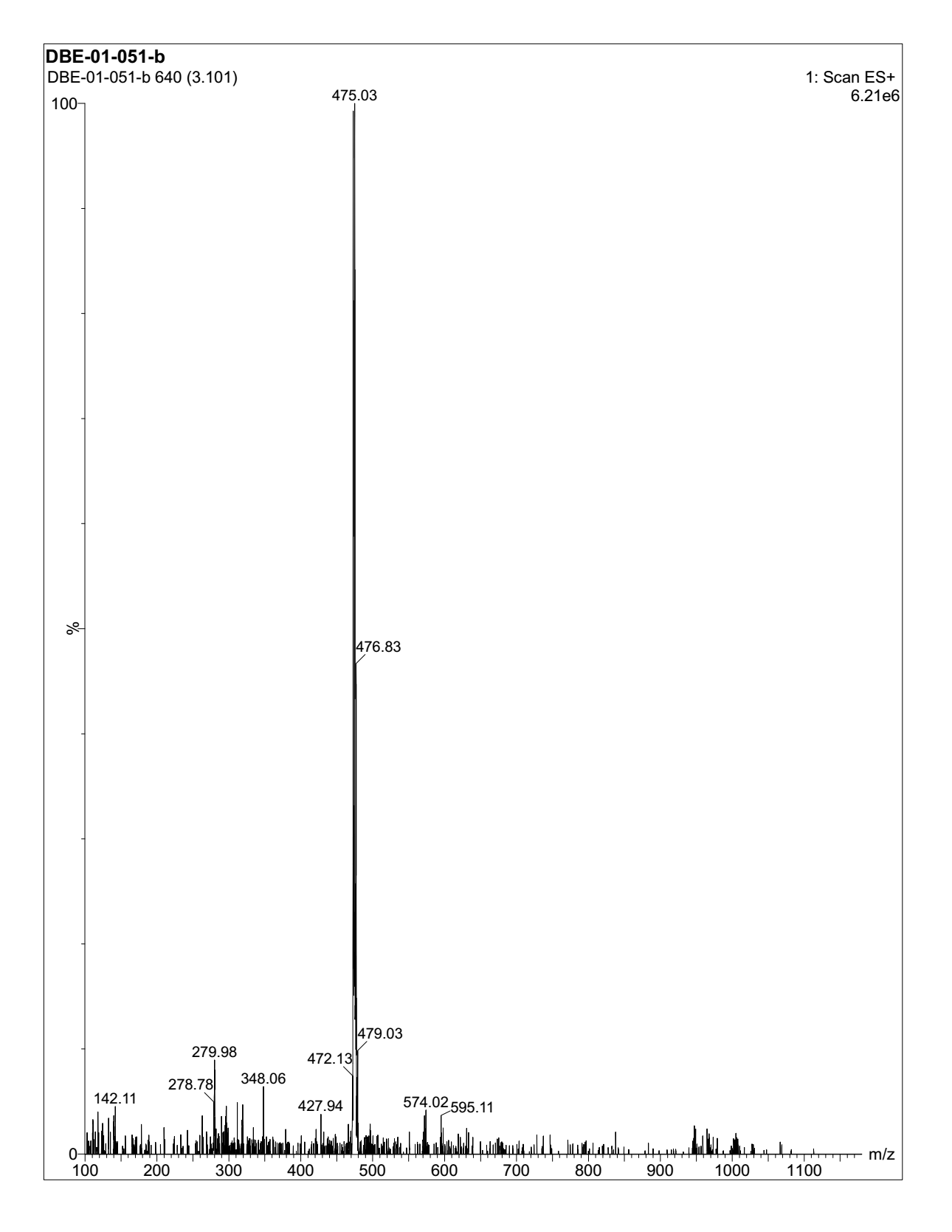


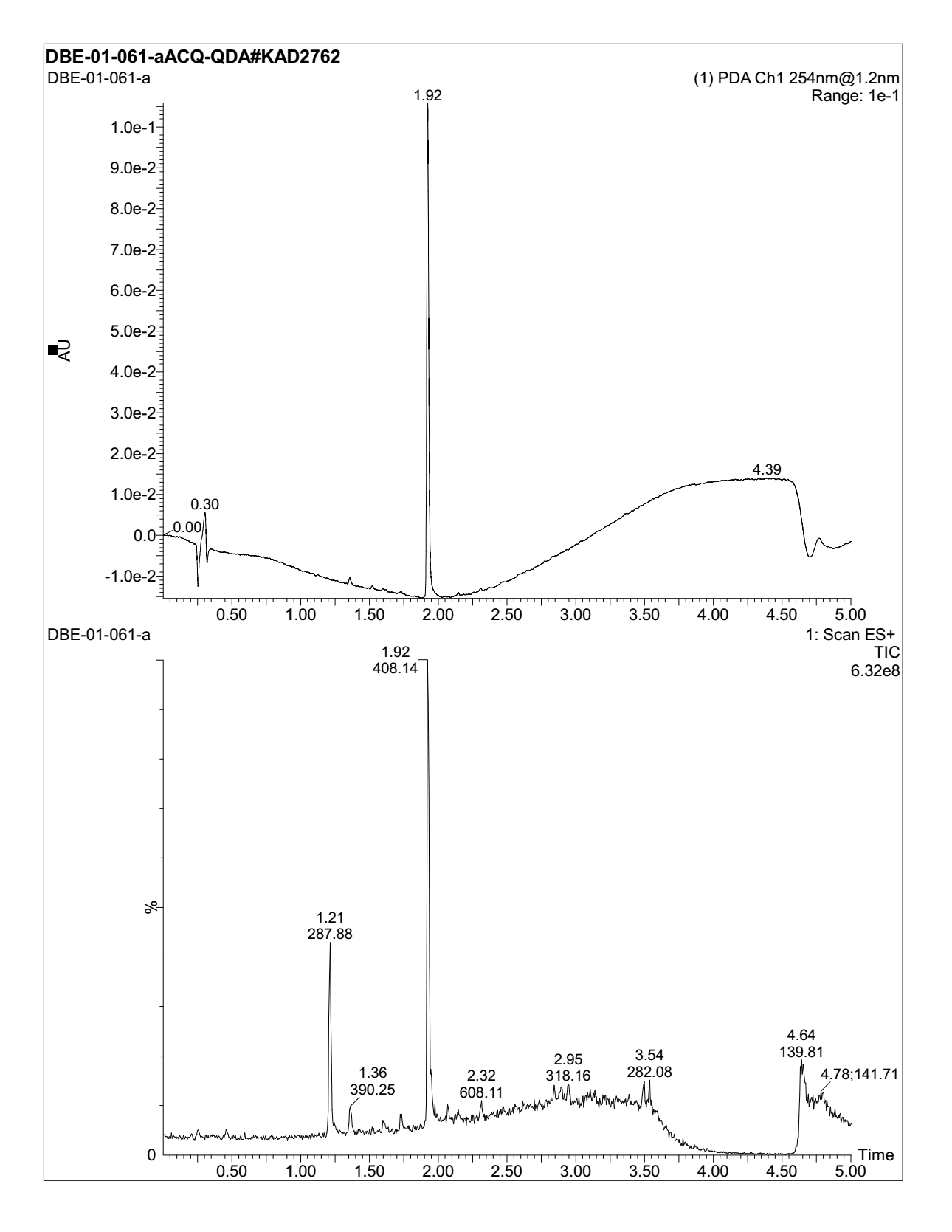


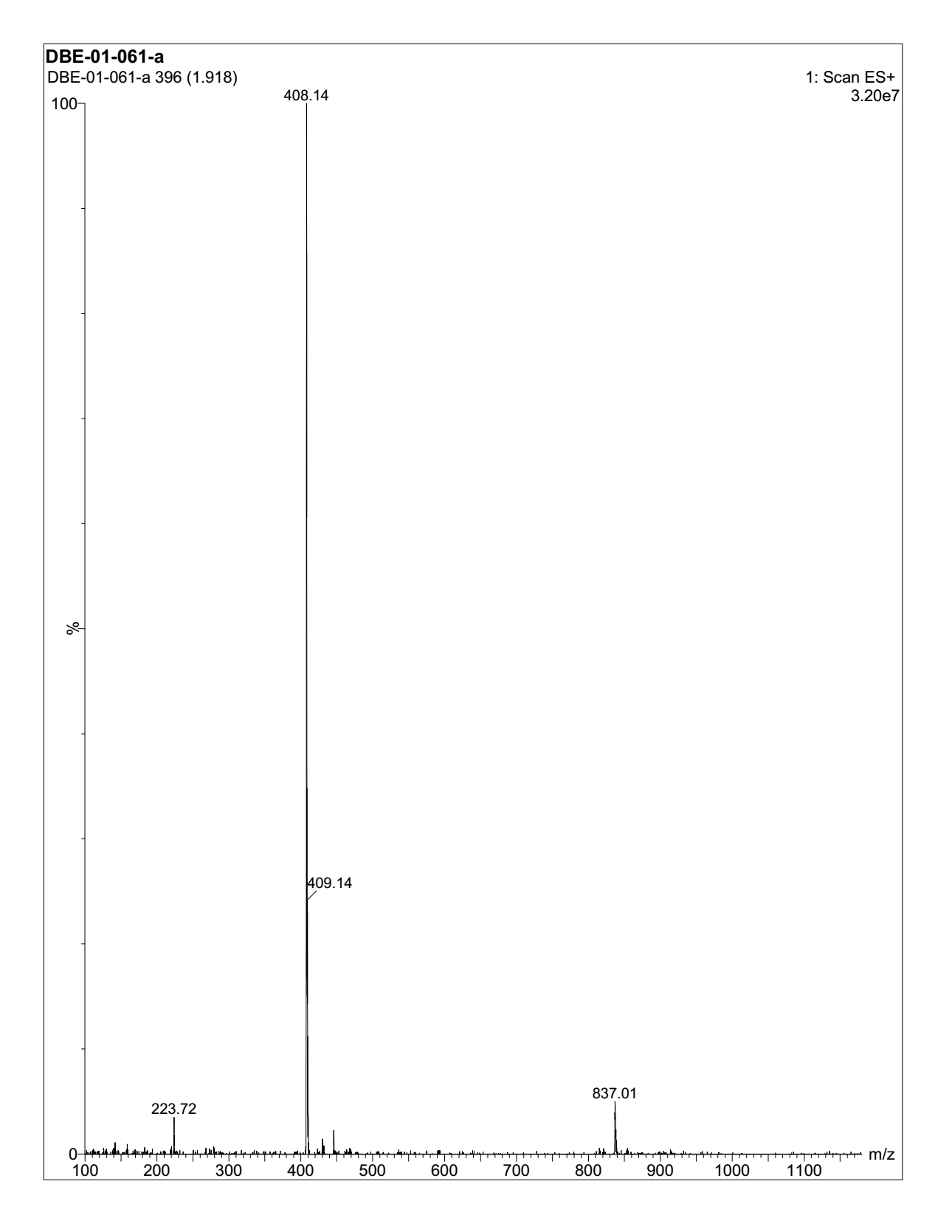


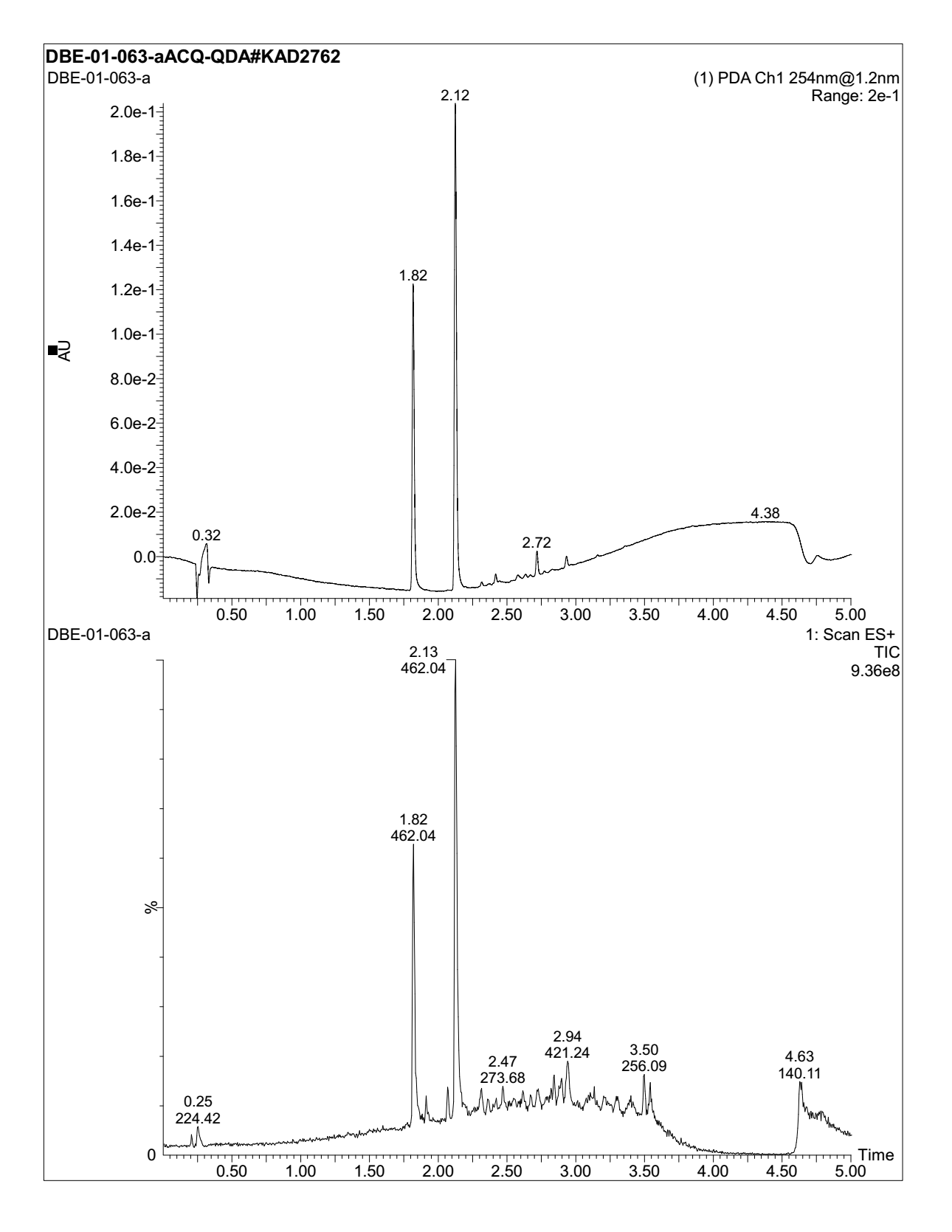


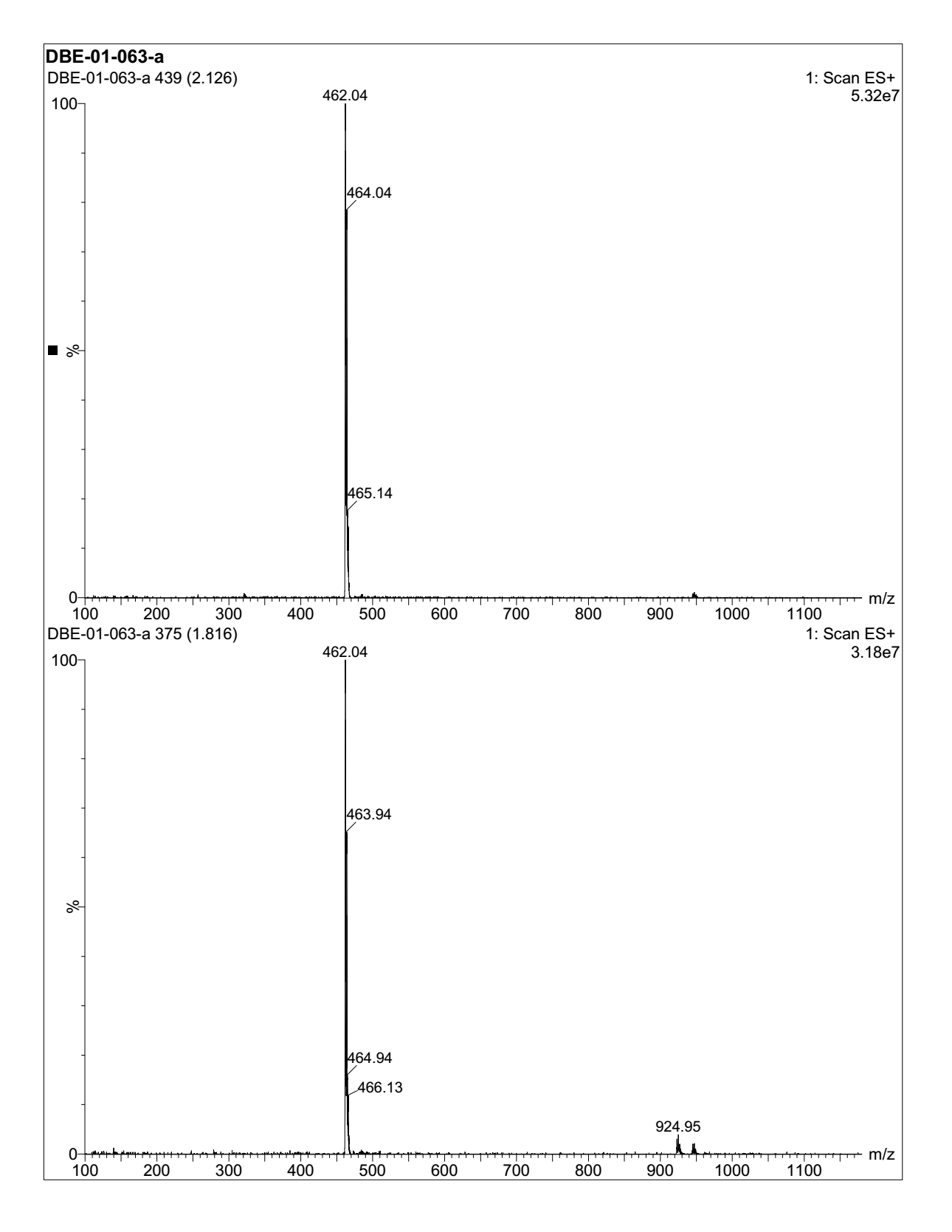


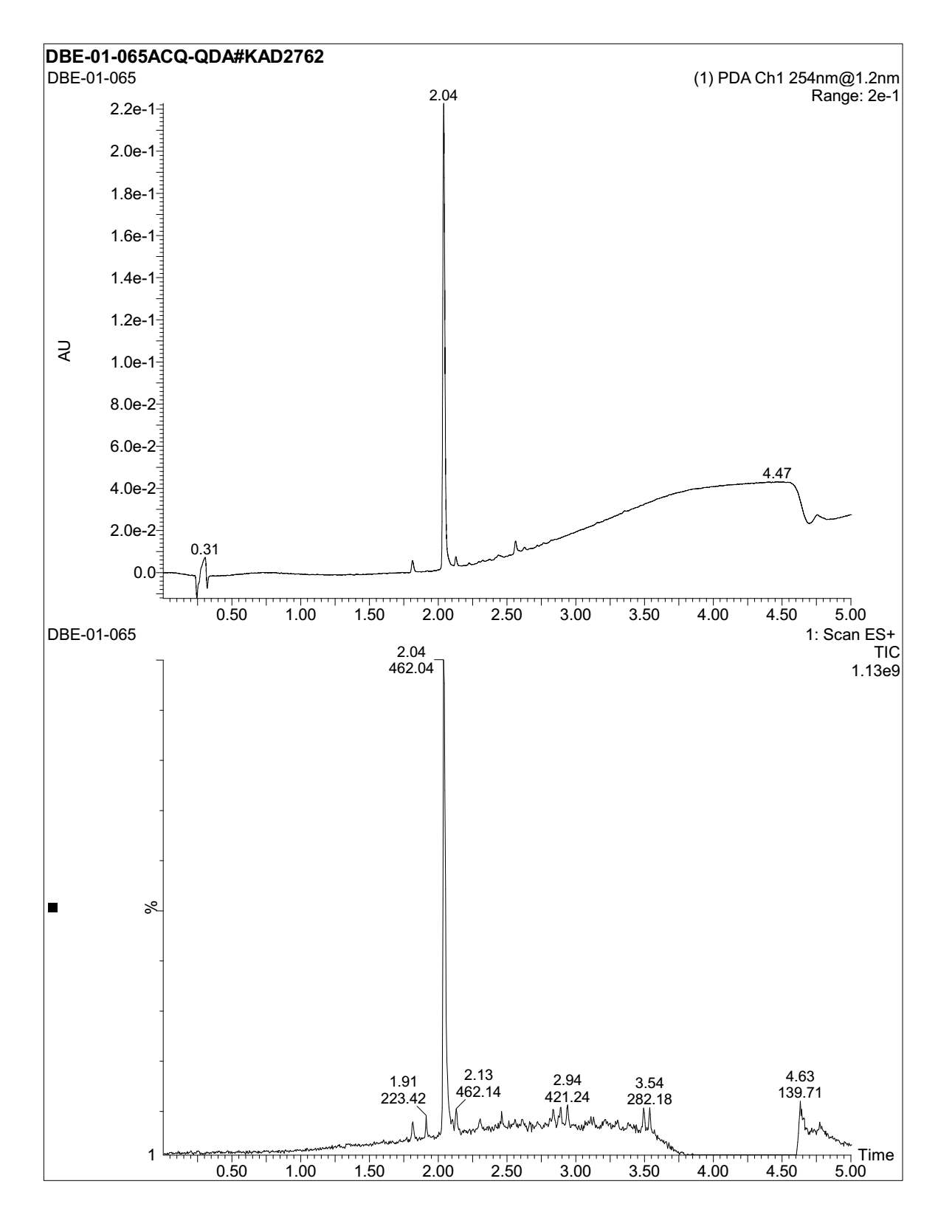


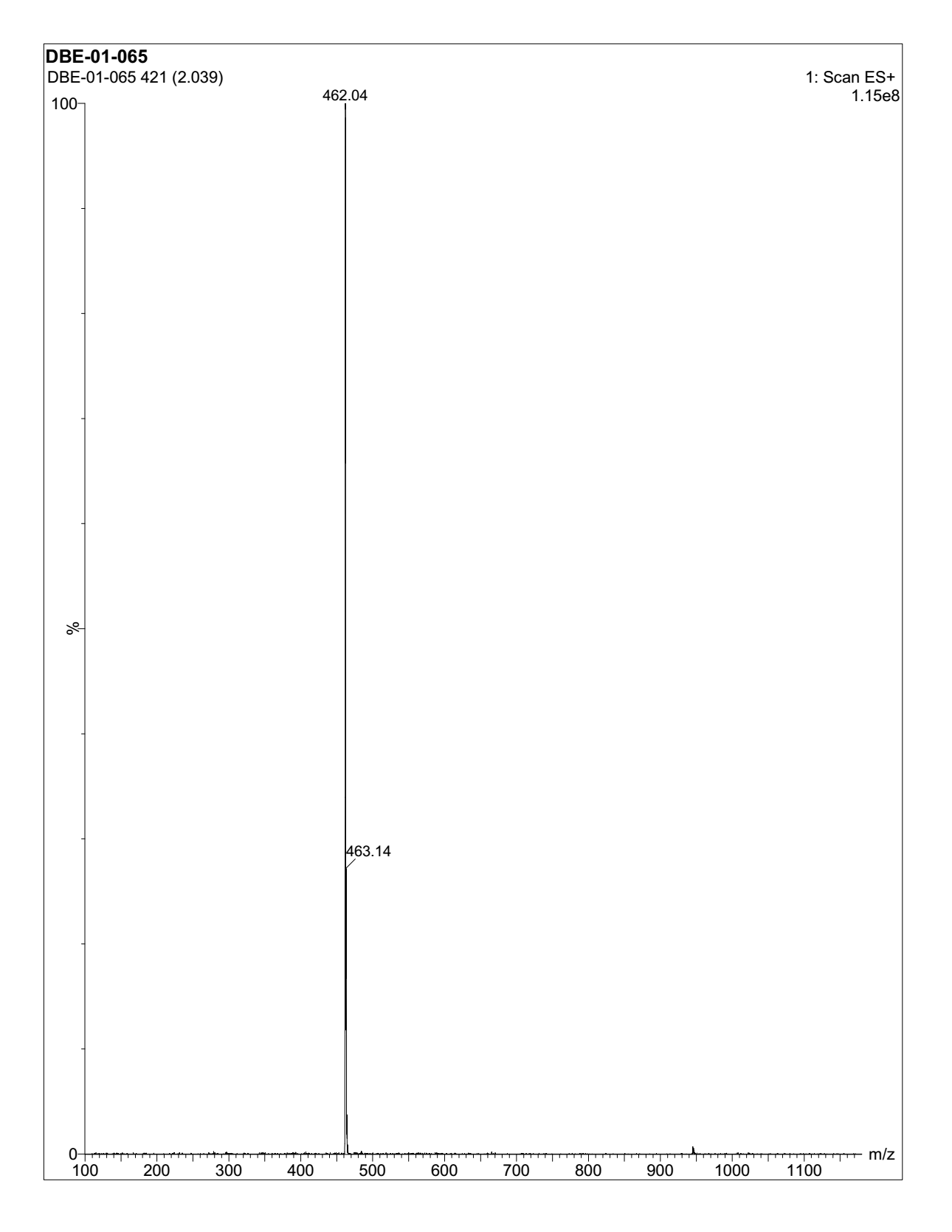


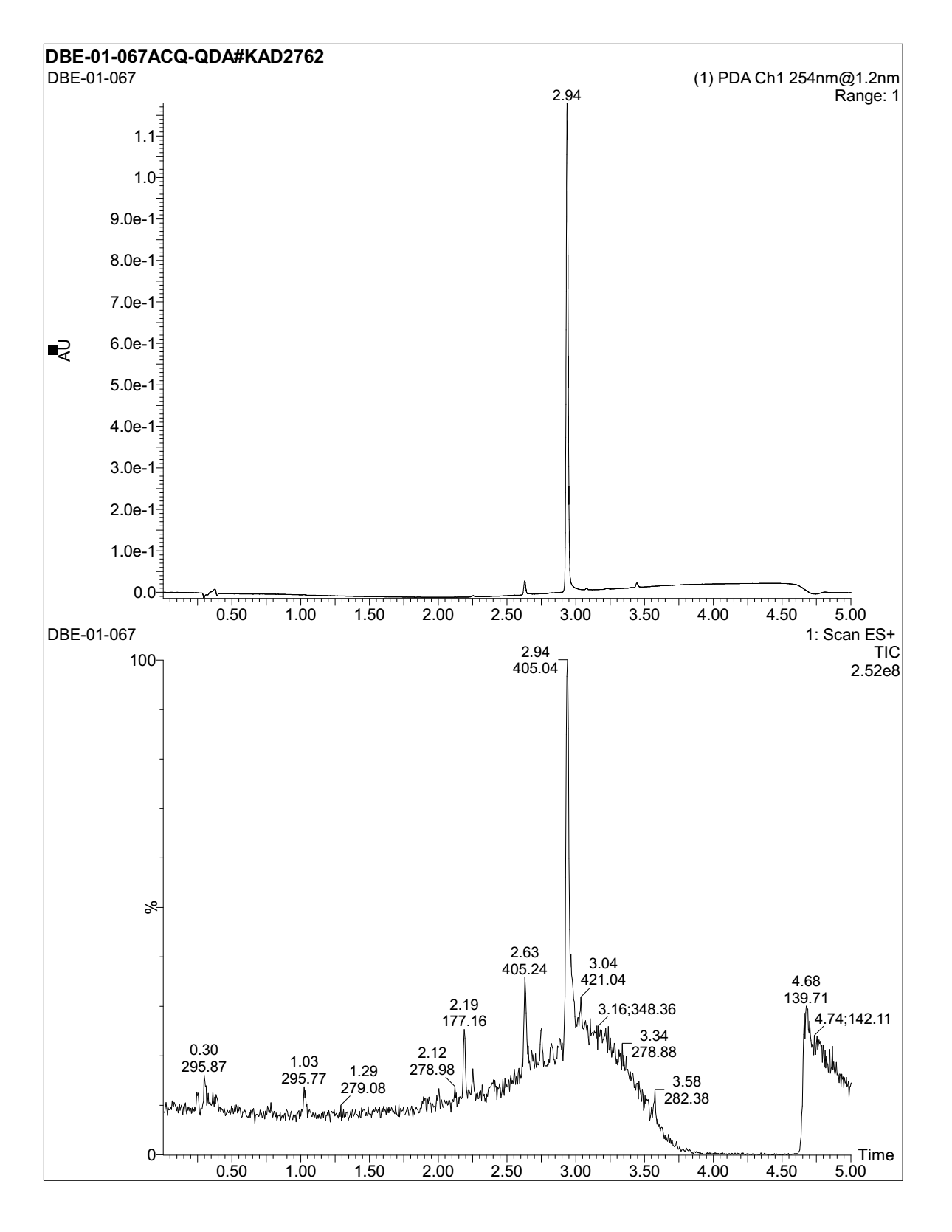


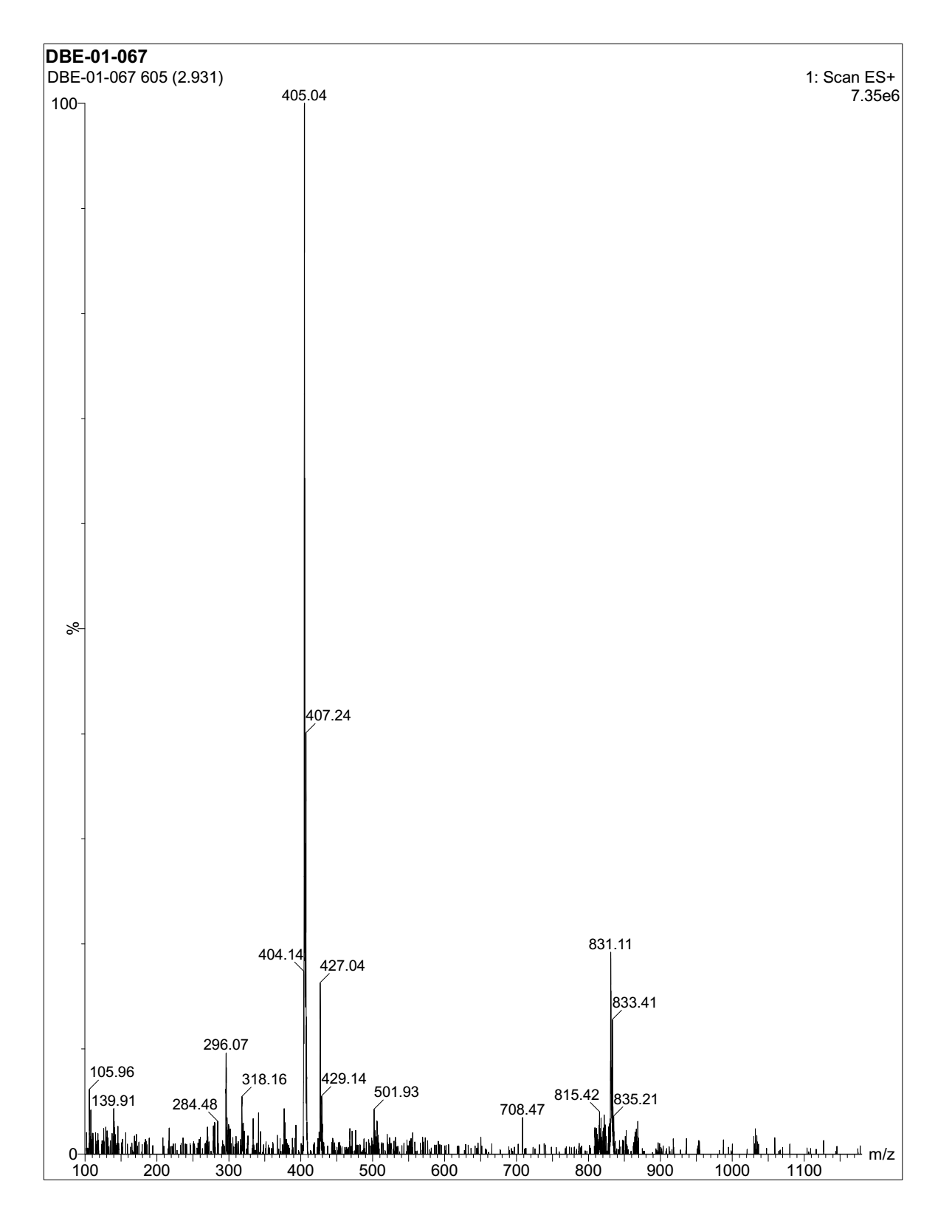


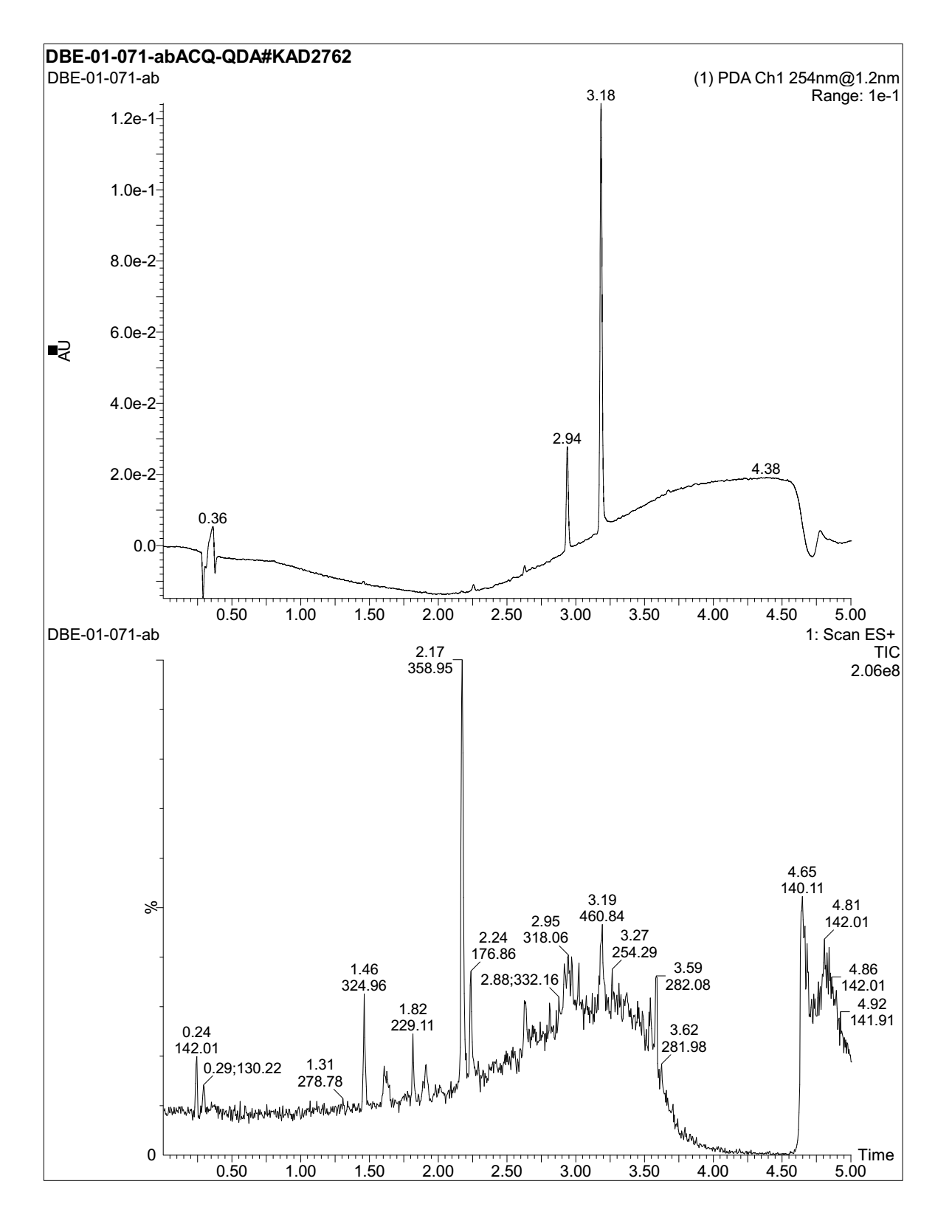


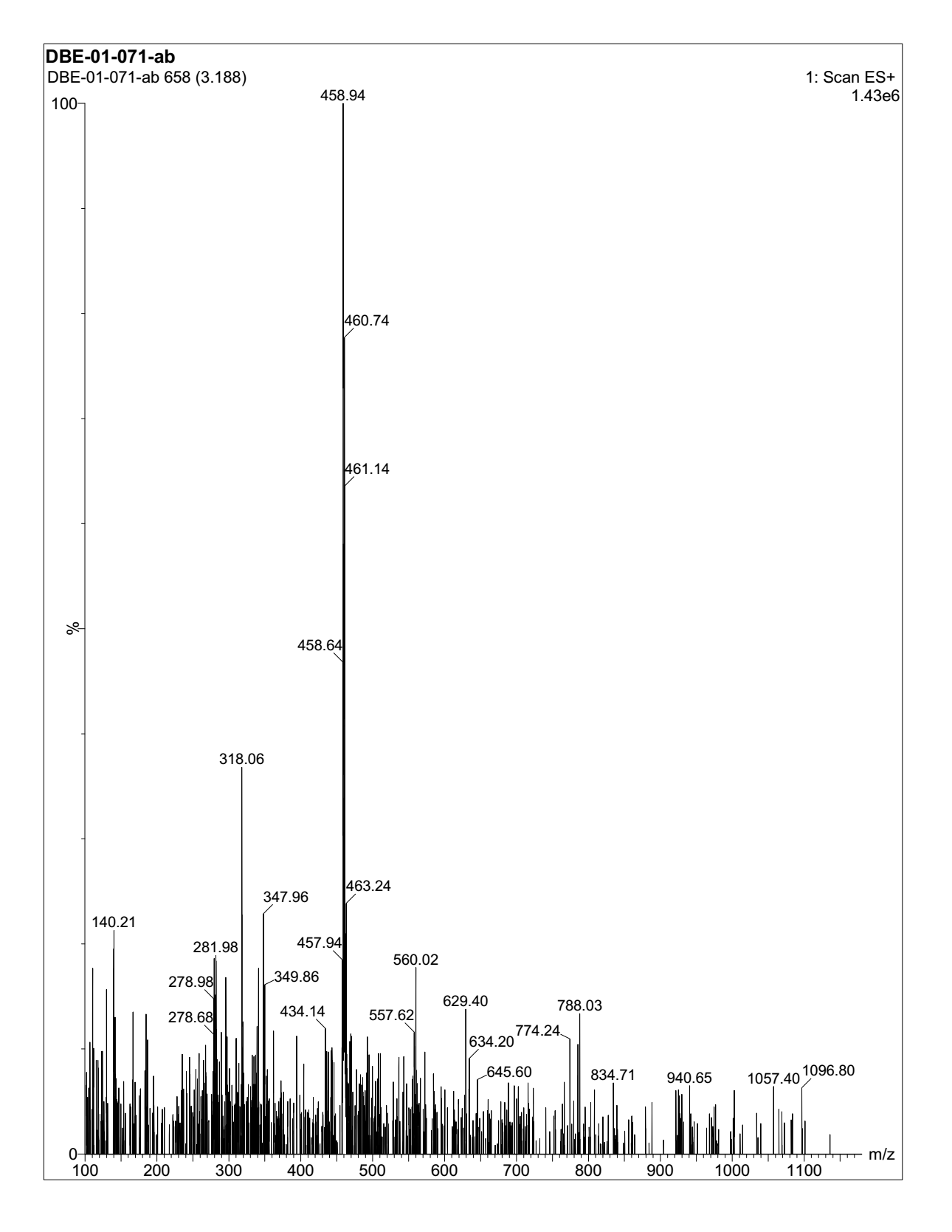


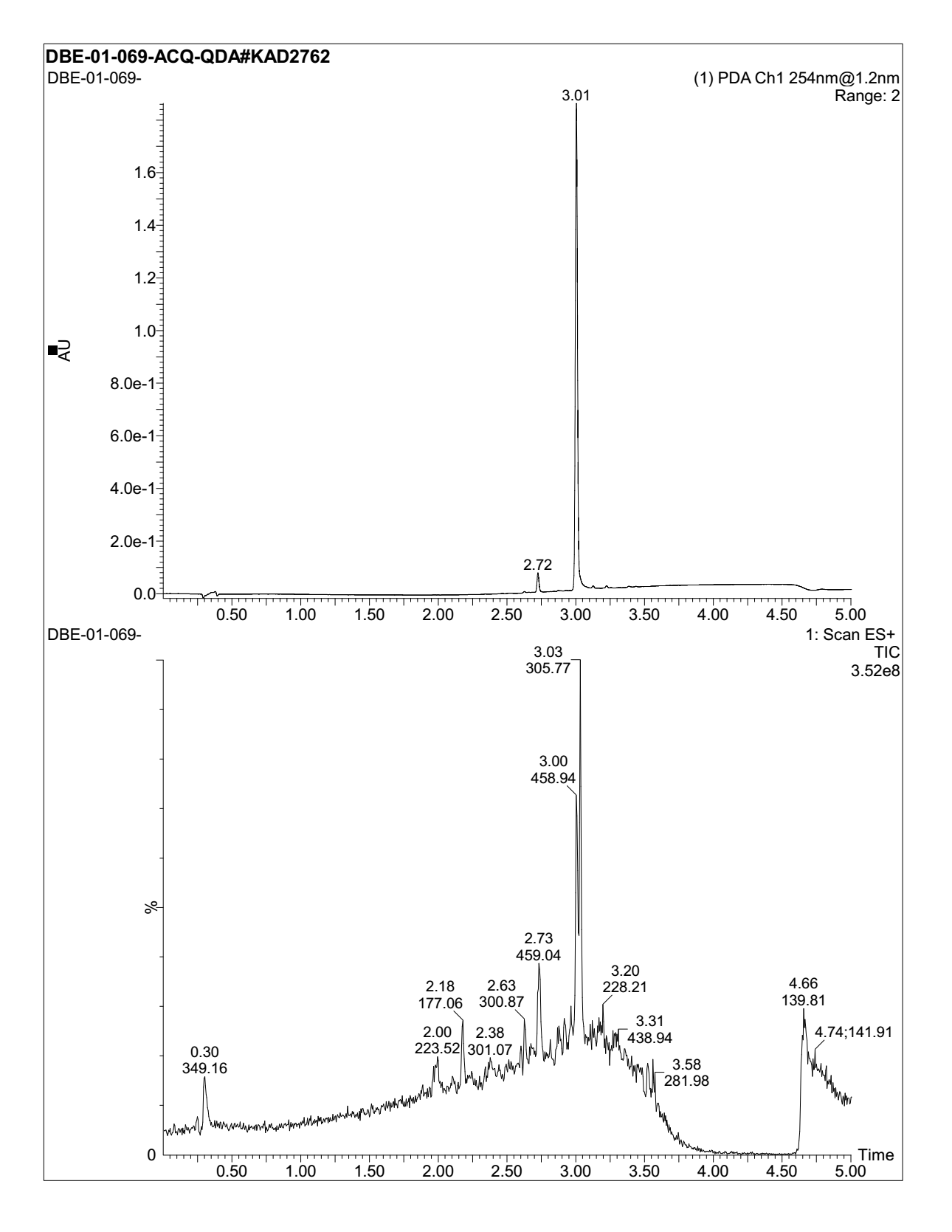


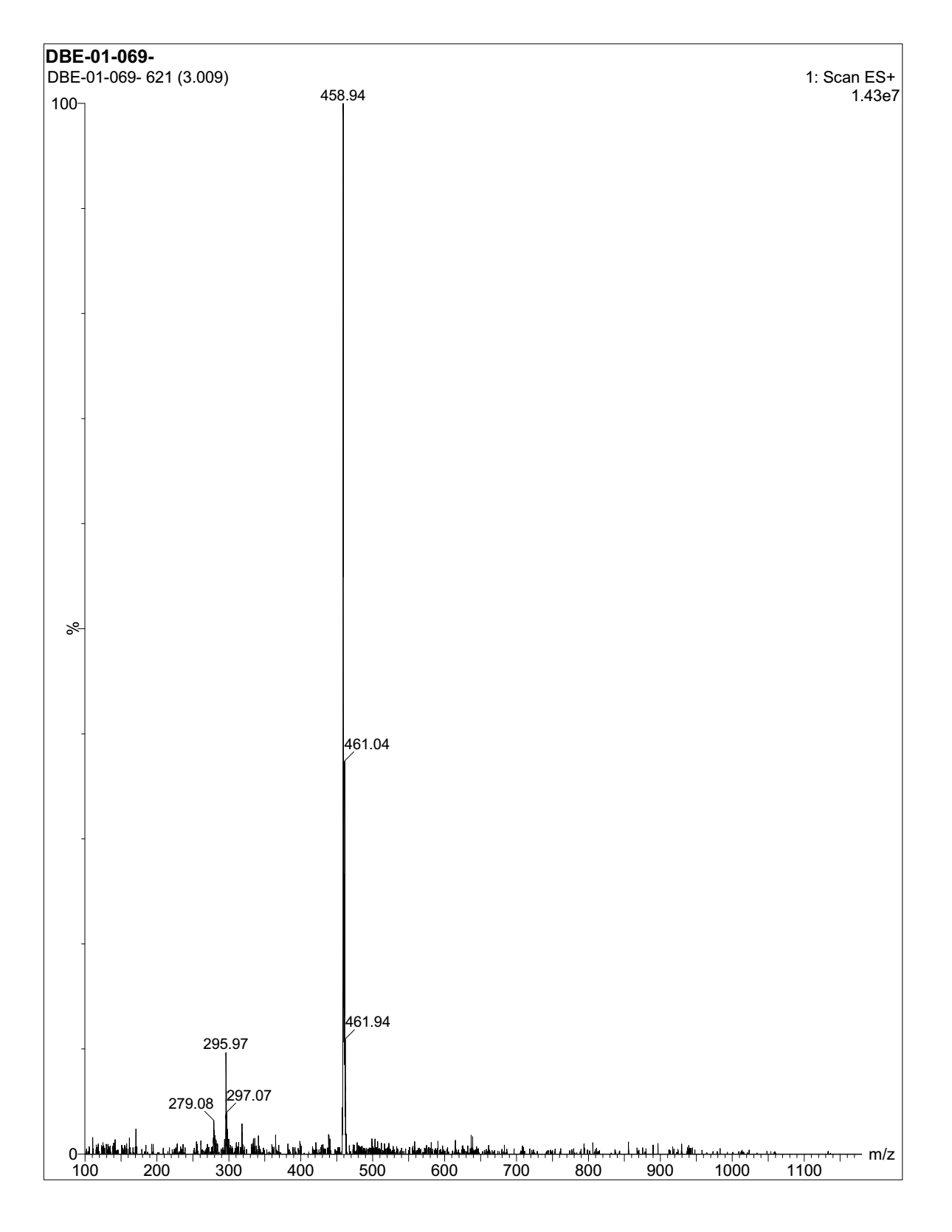


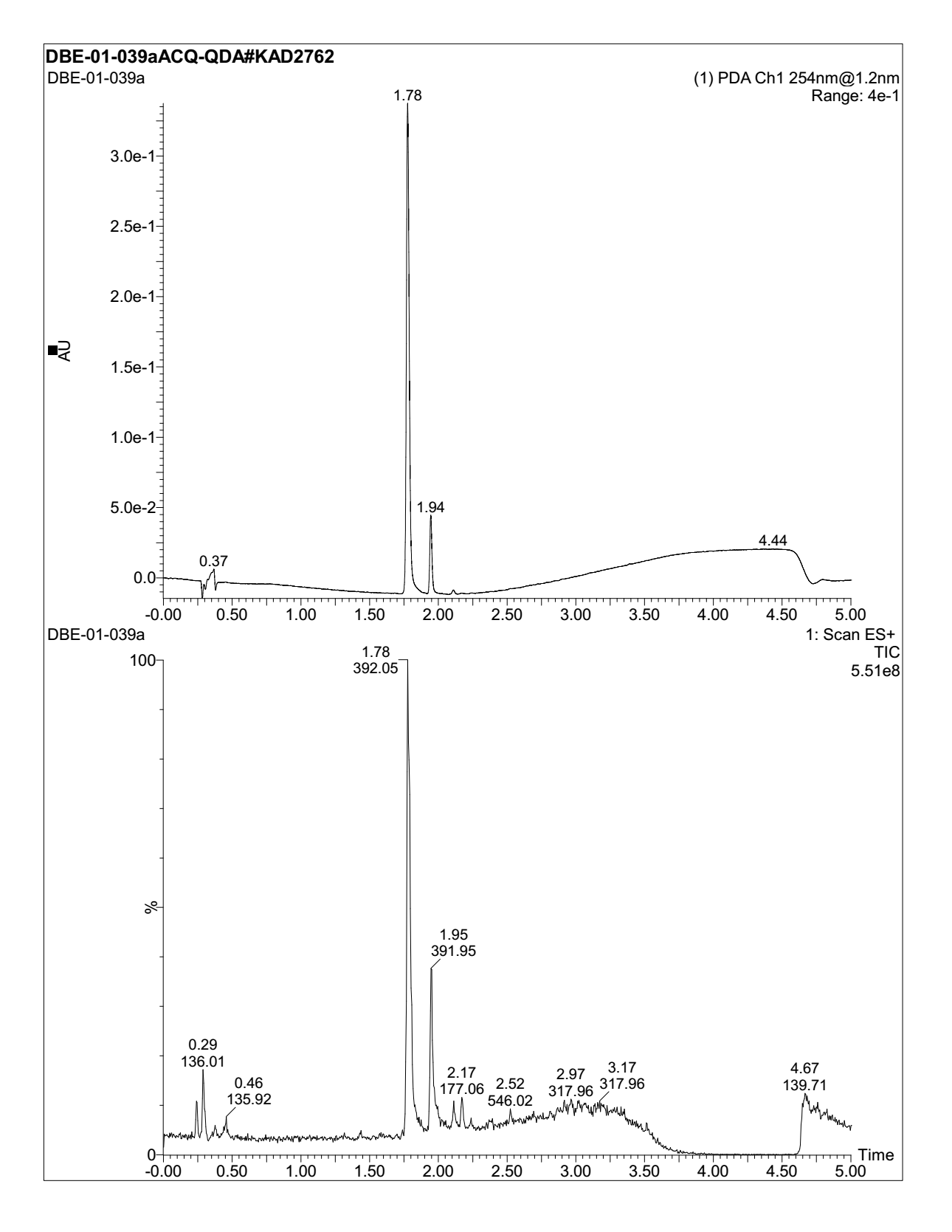


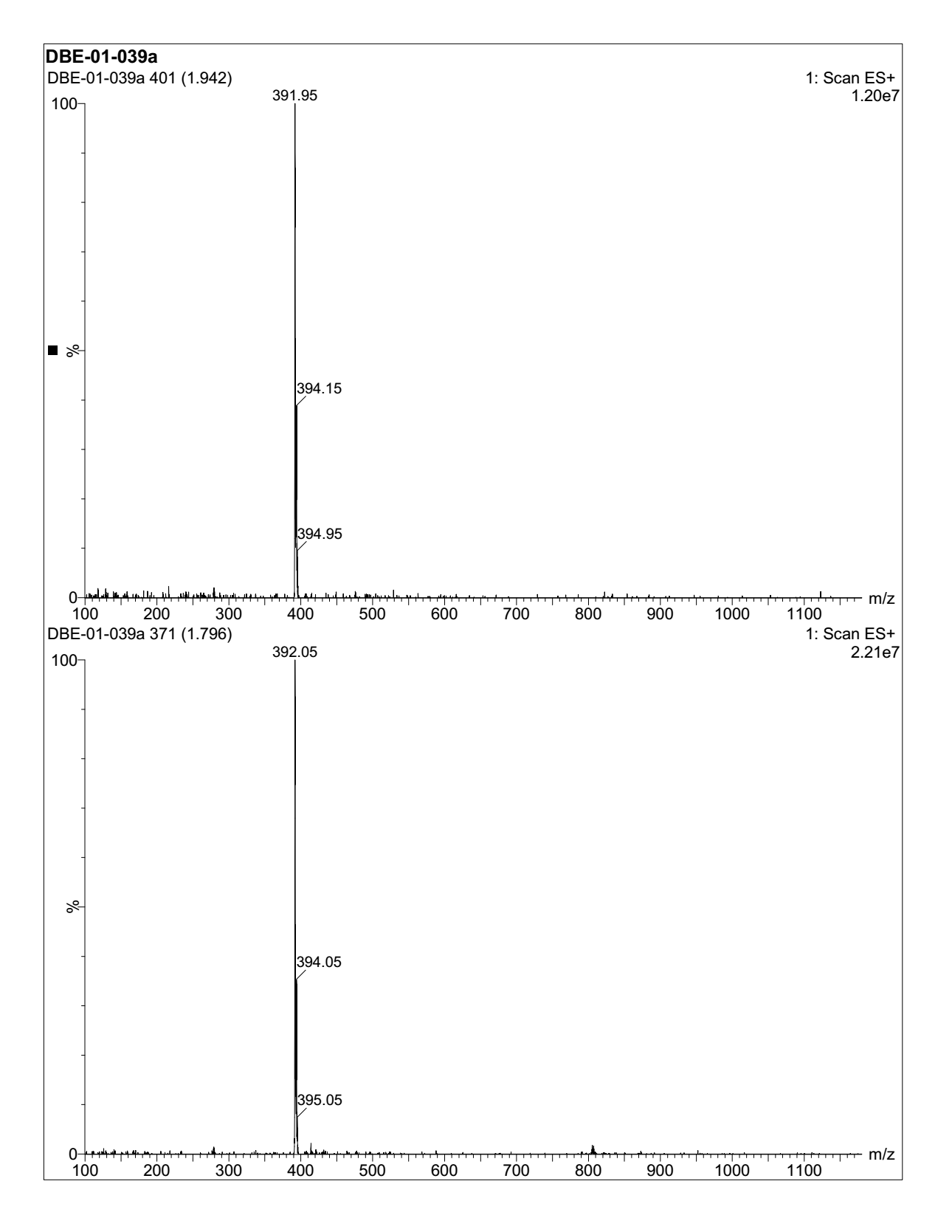


**PROTON-NMR**


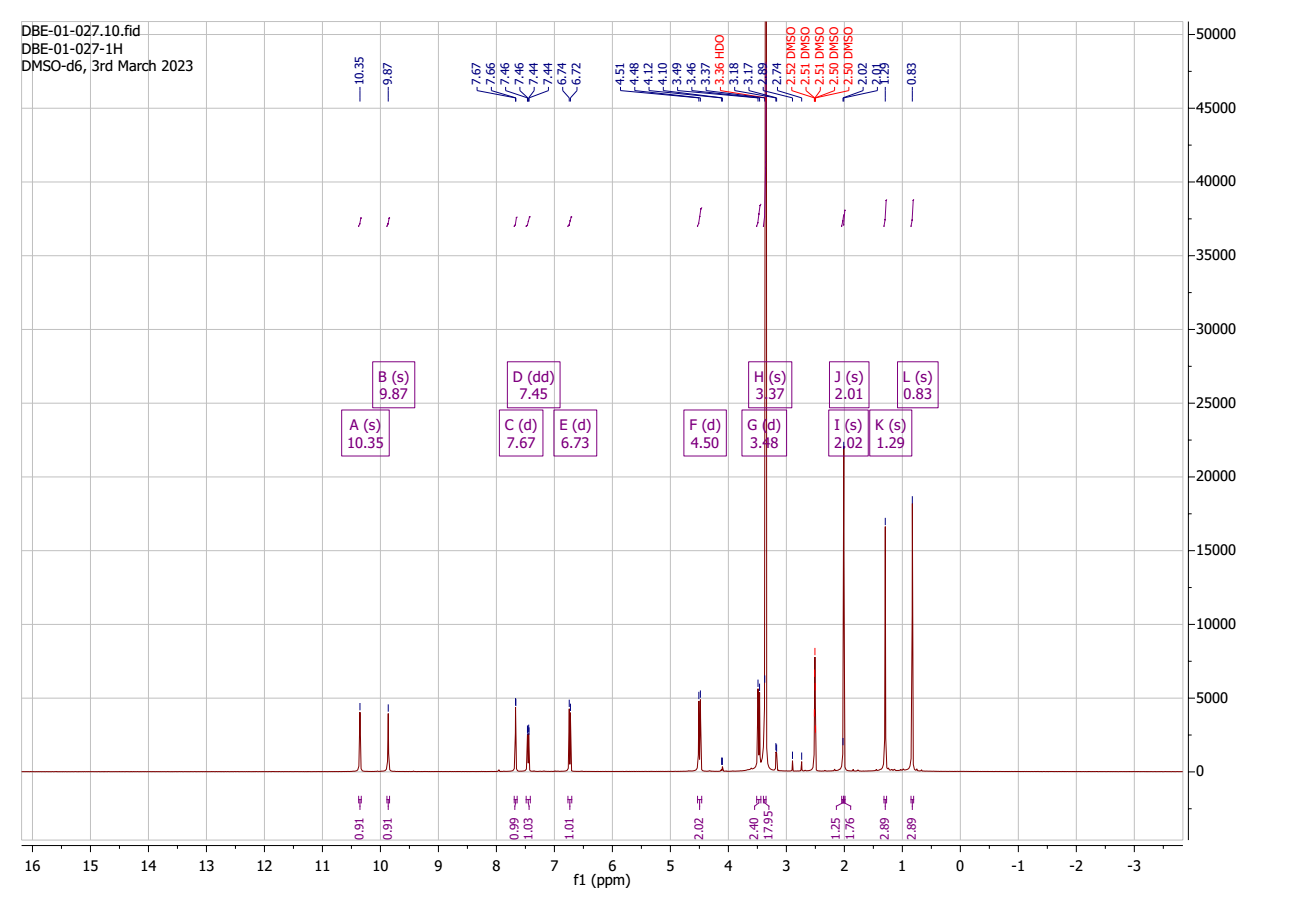


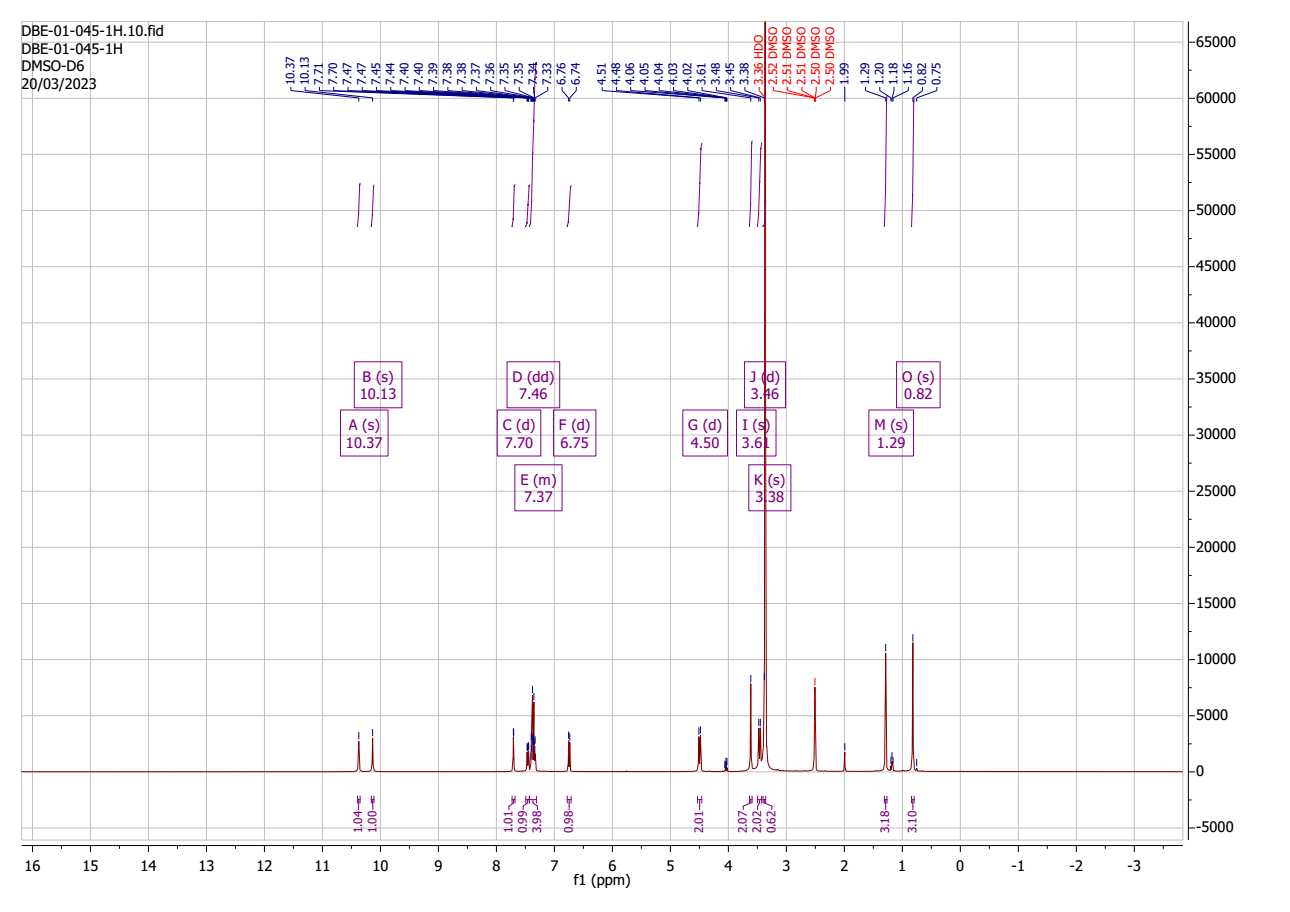


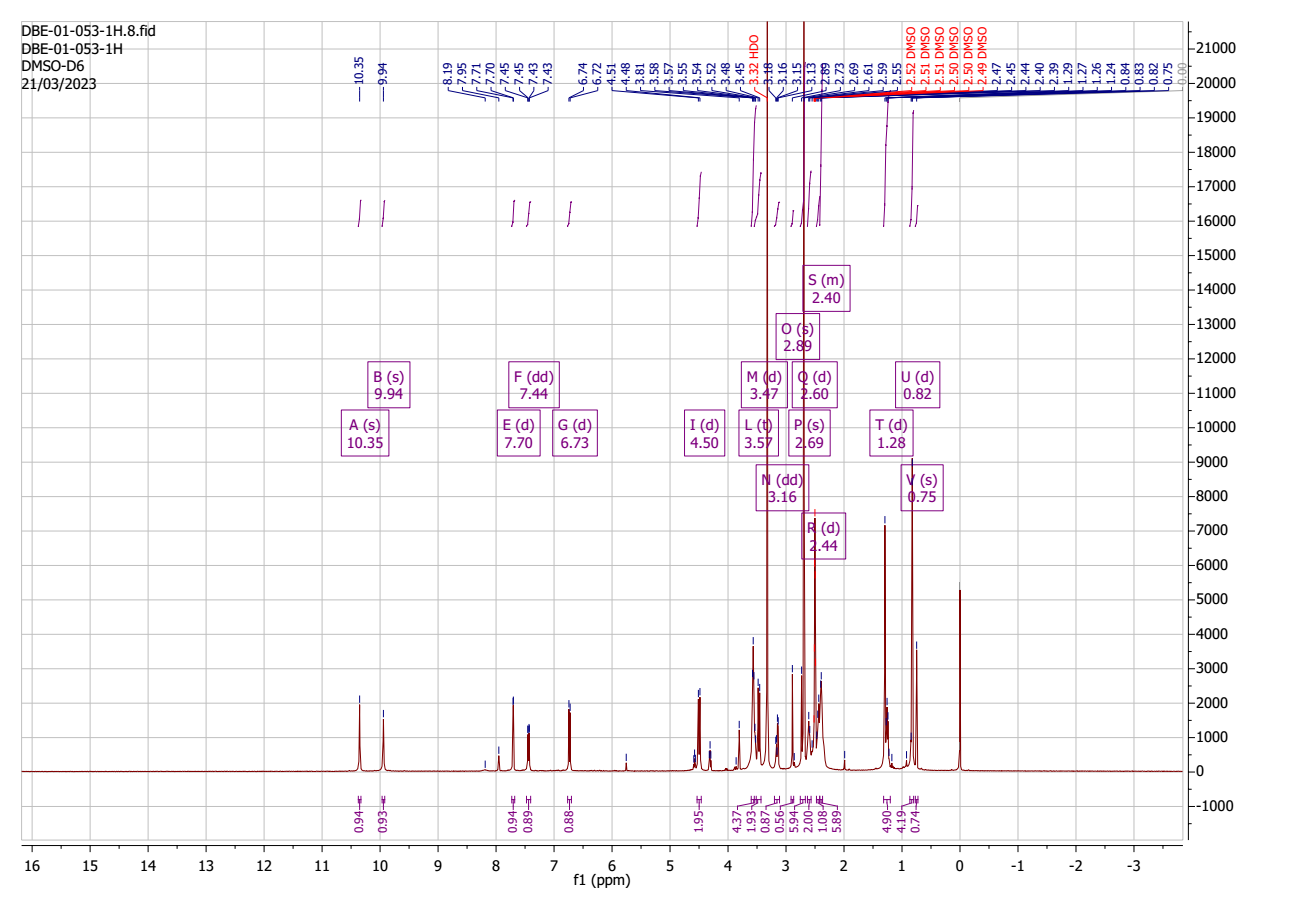


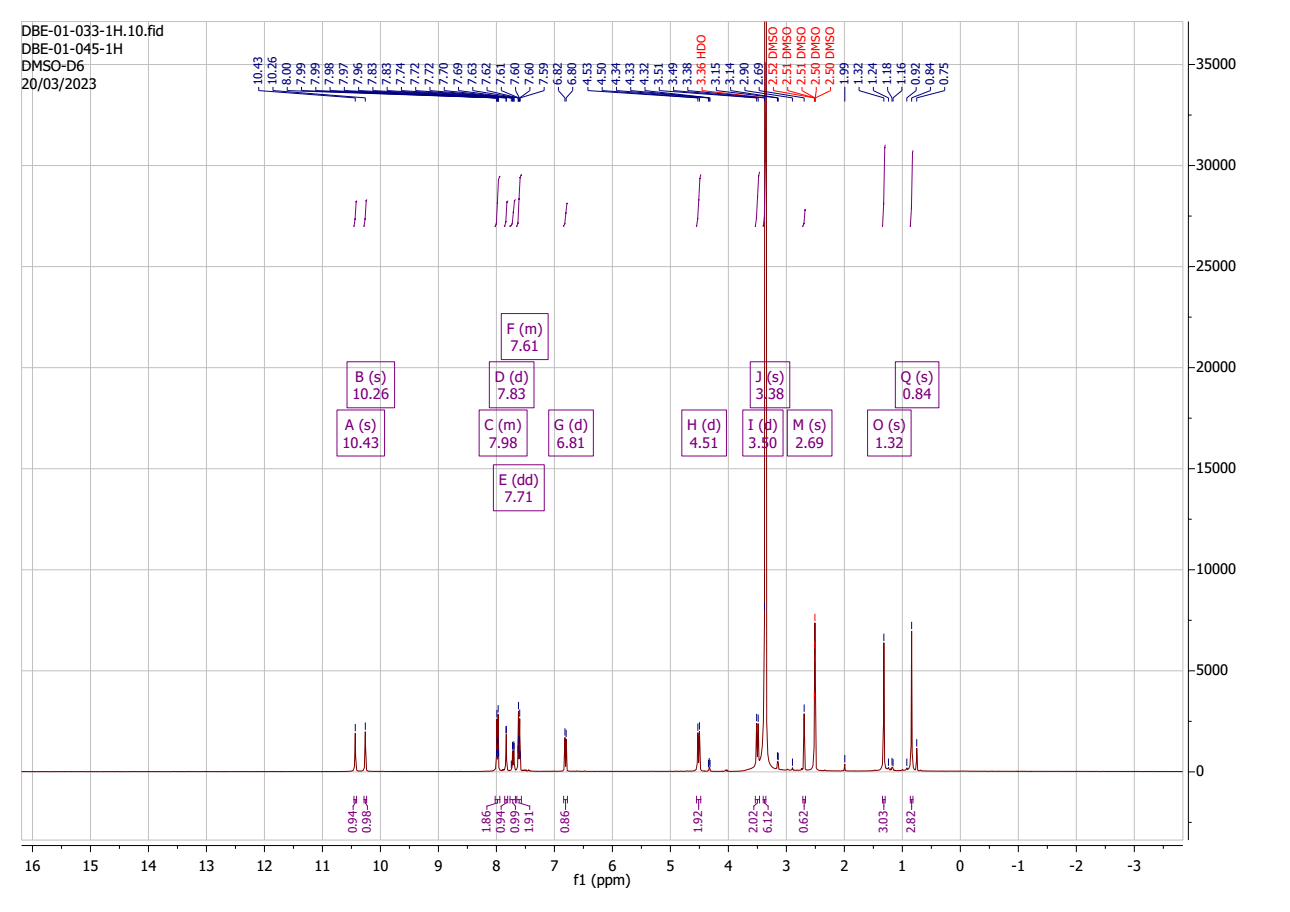


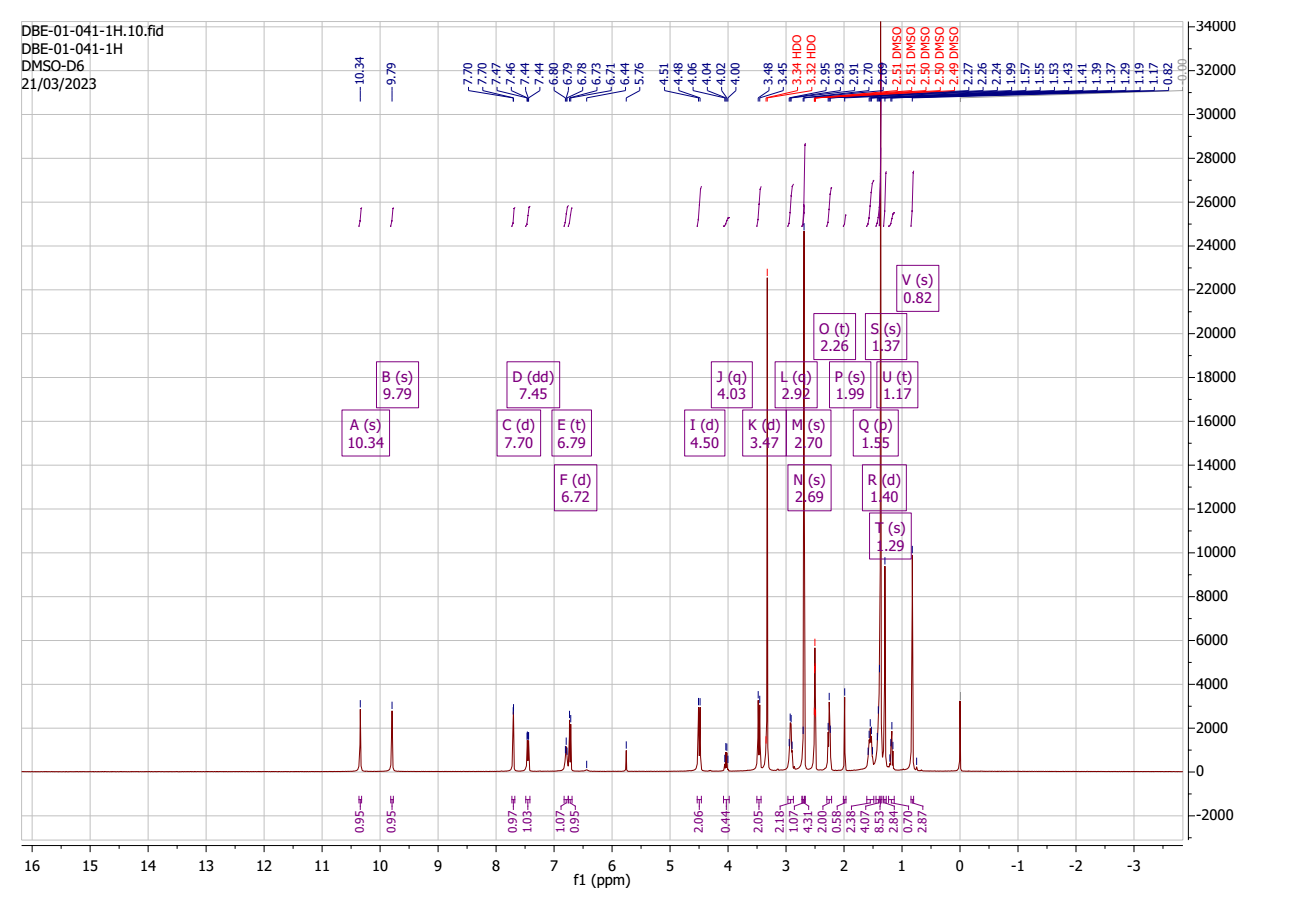


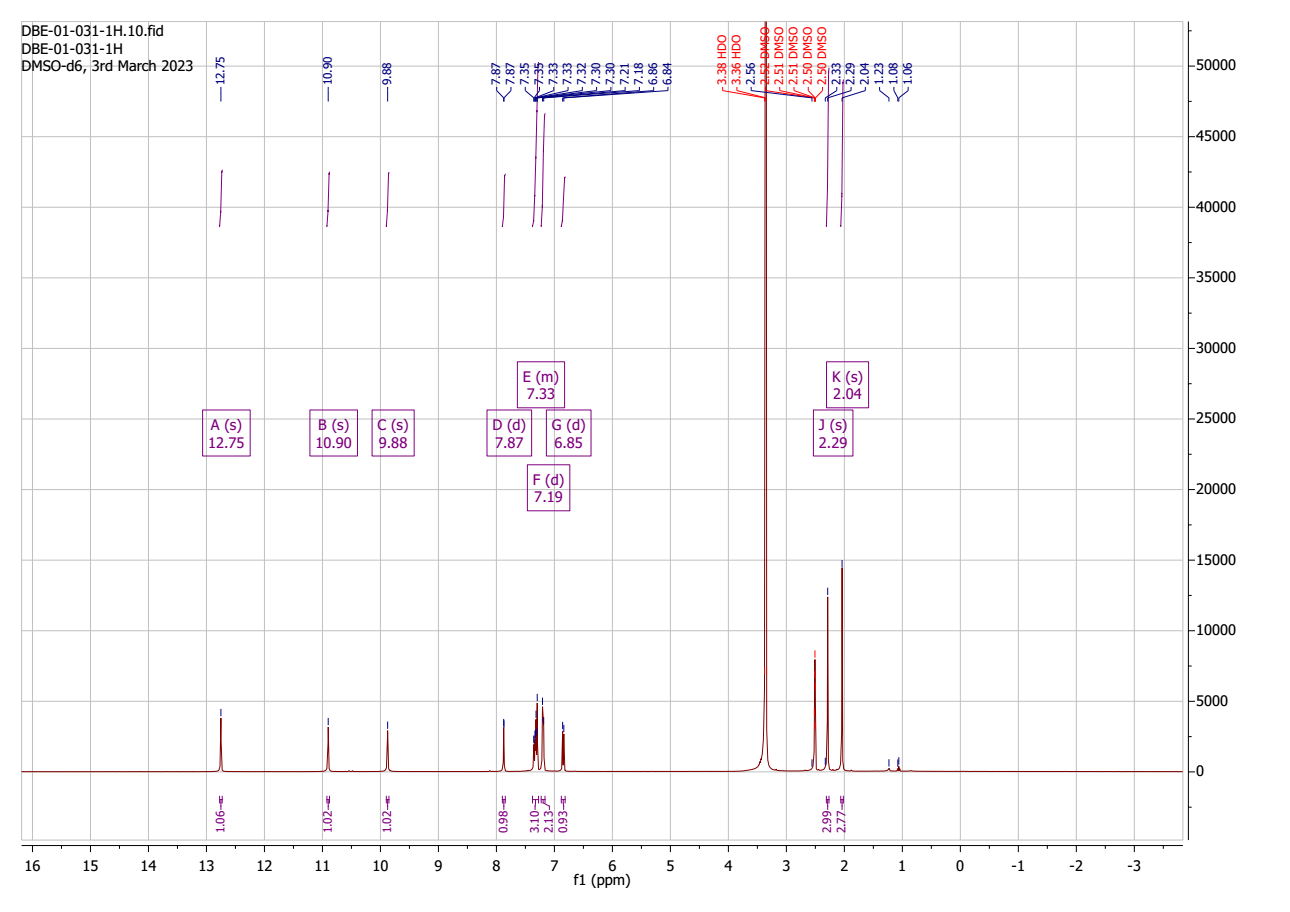


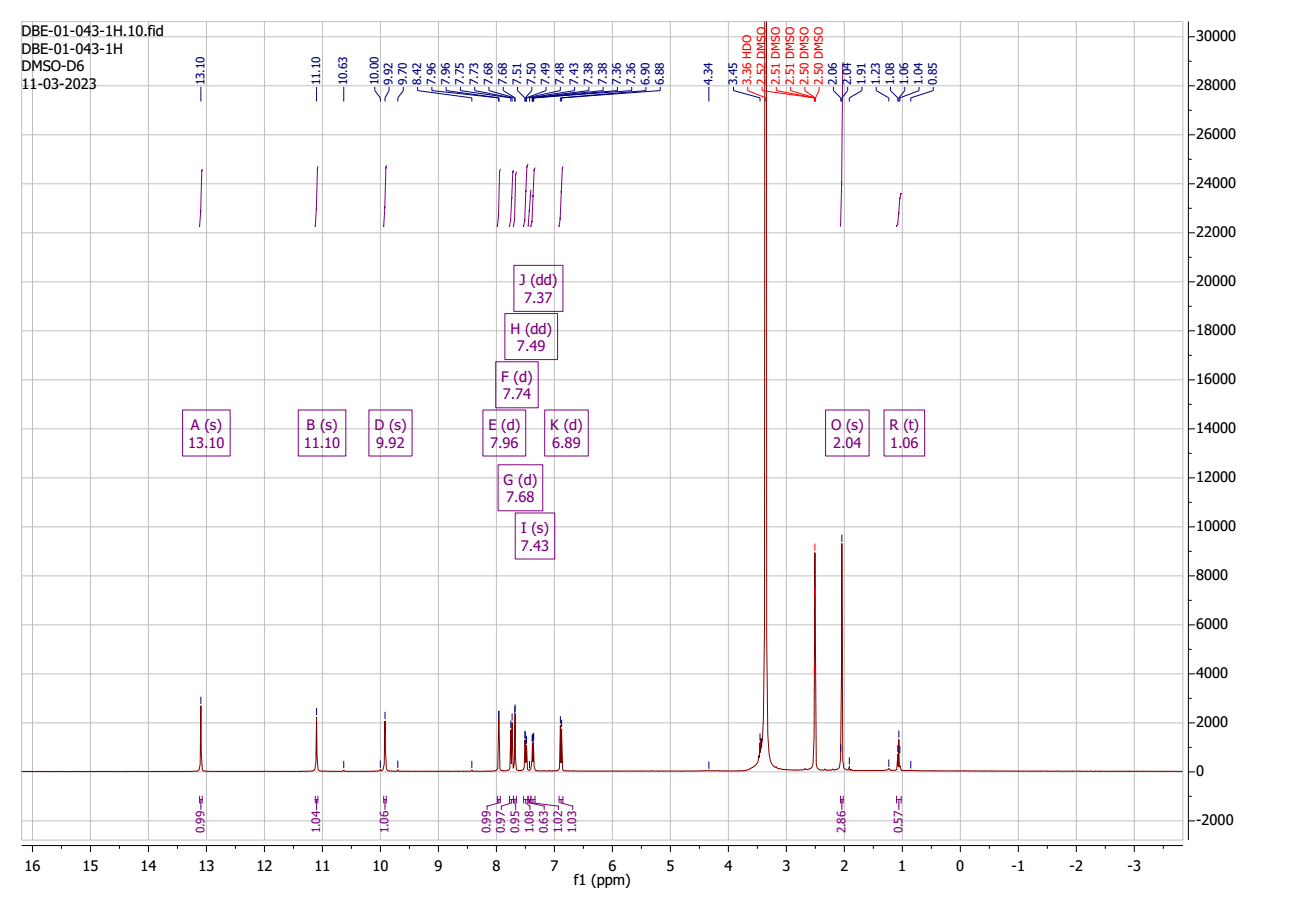


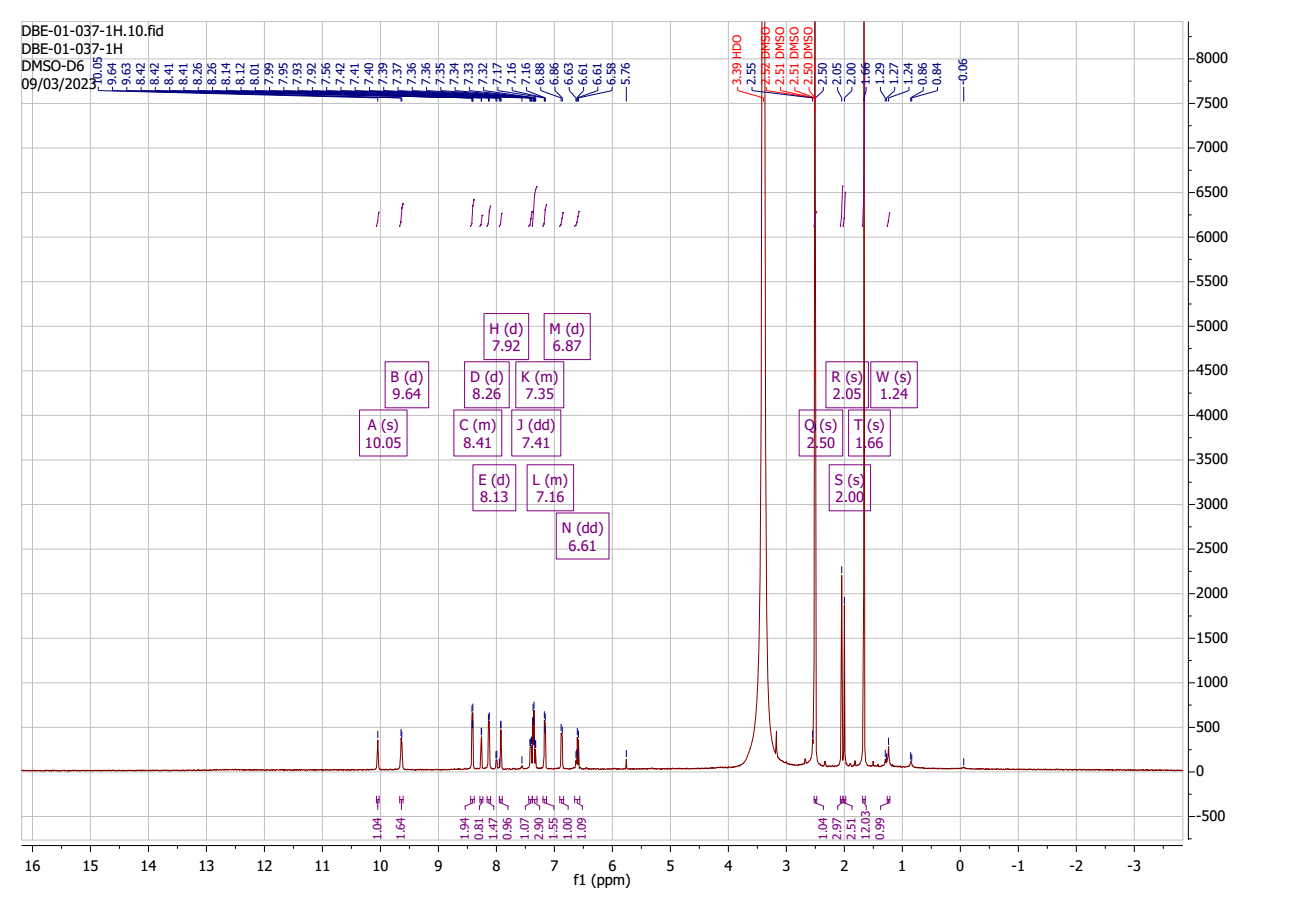


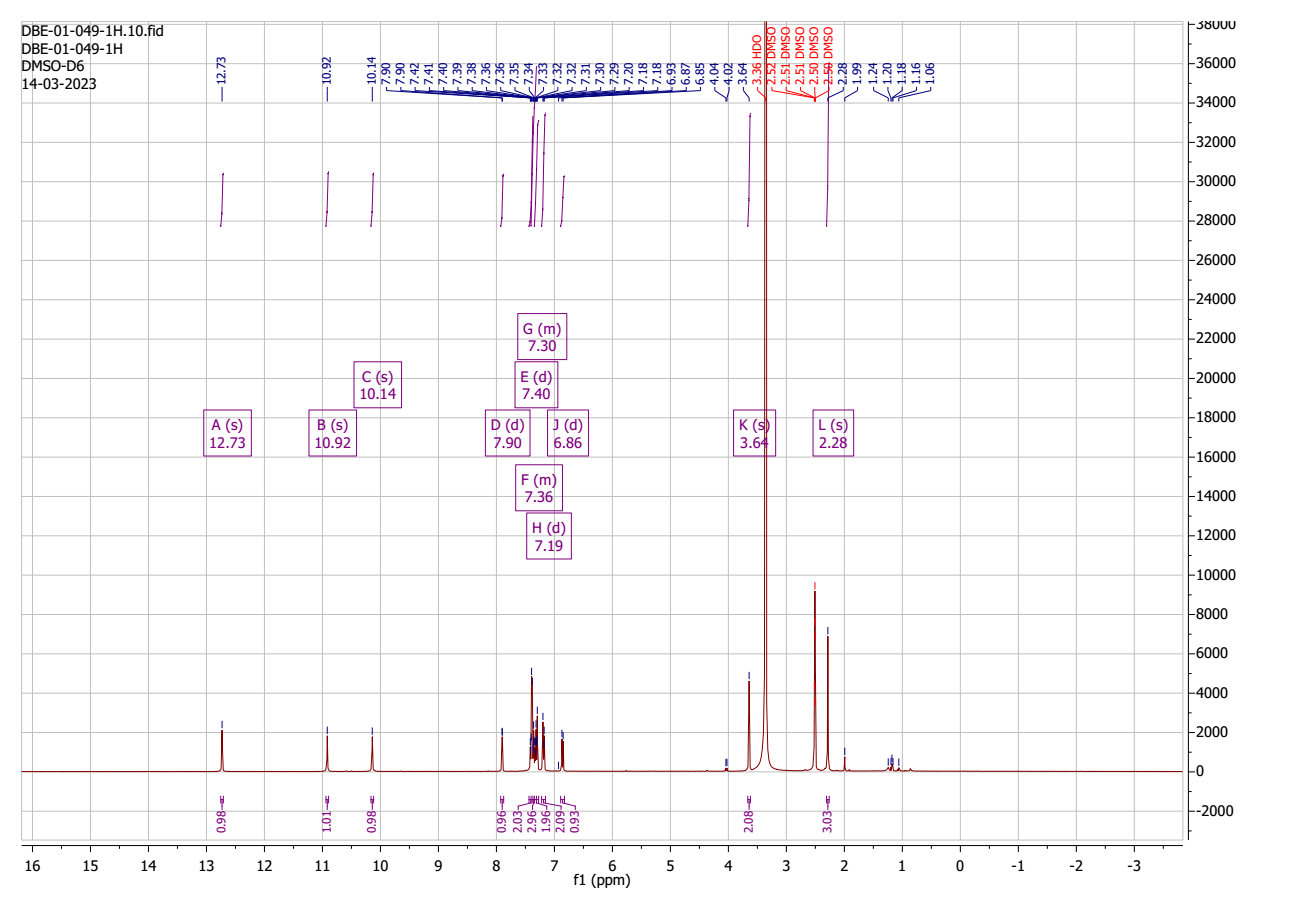


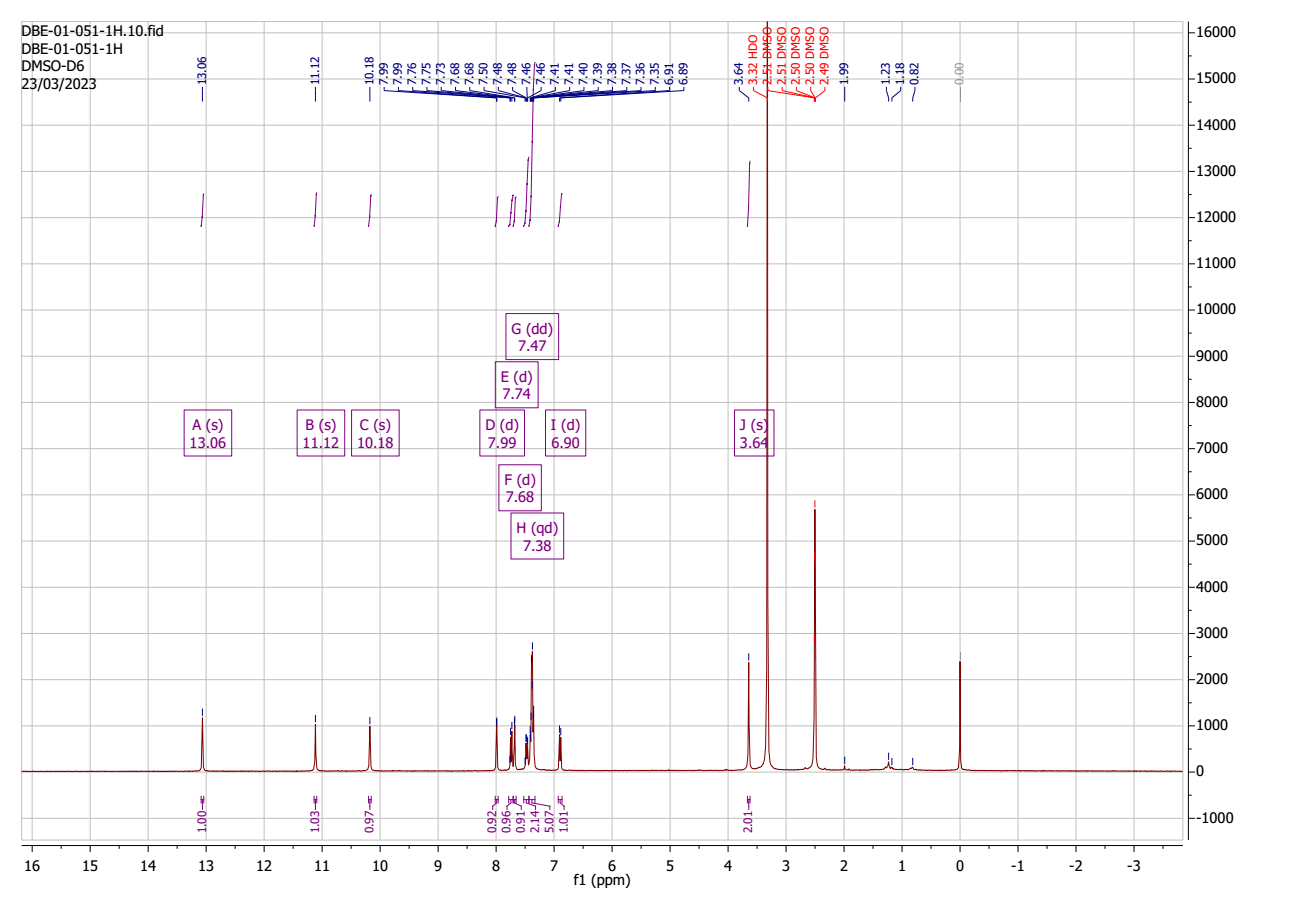


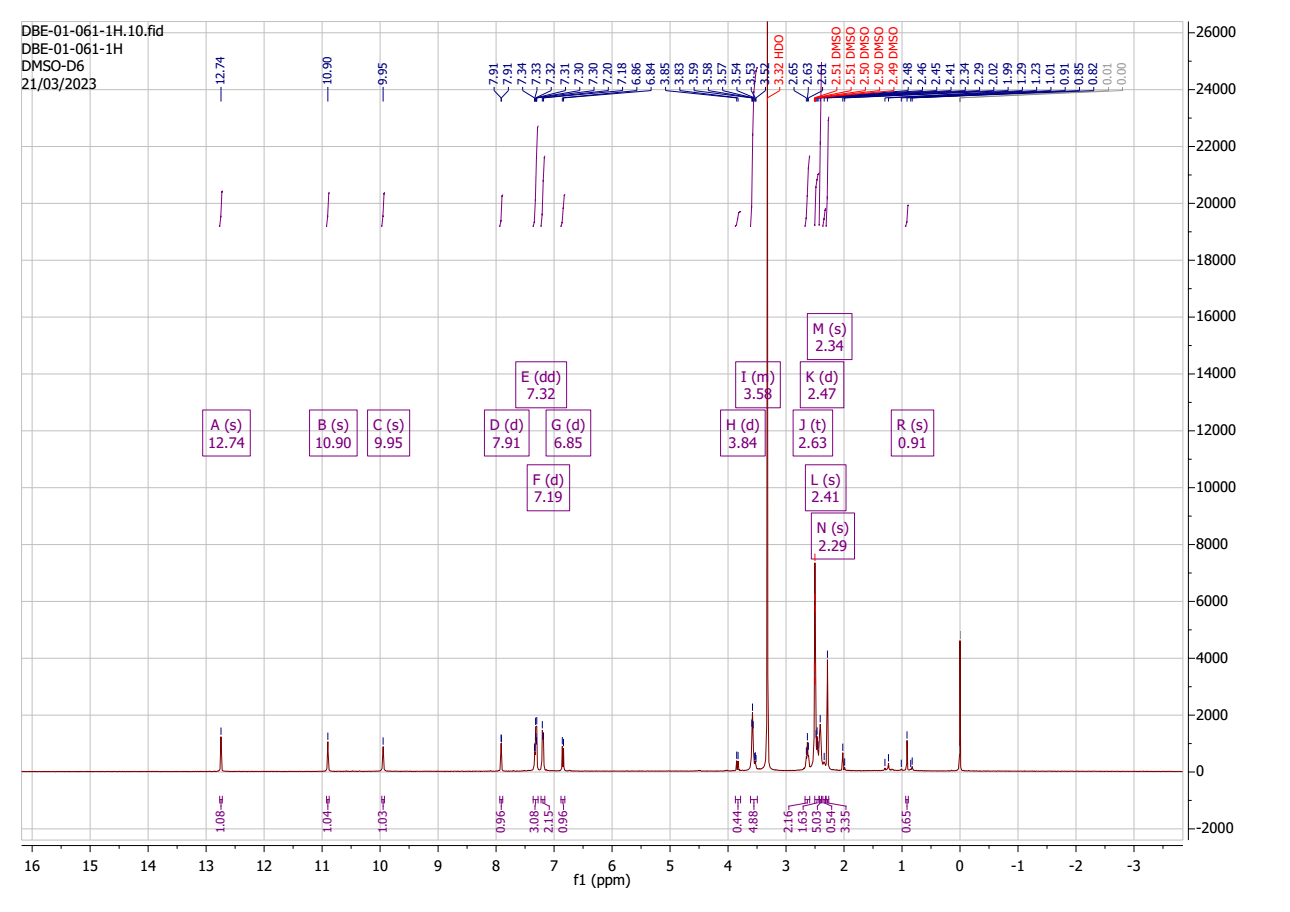


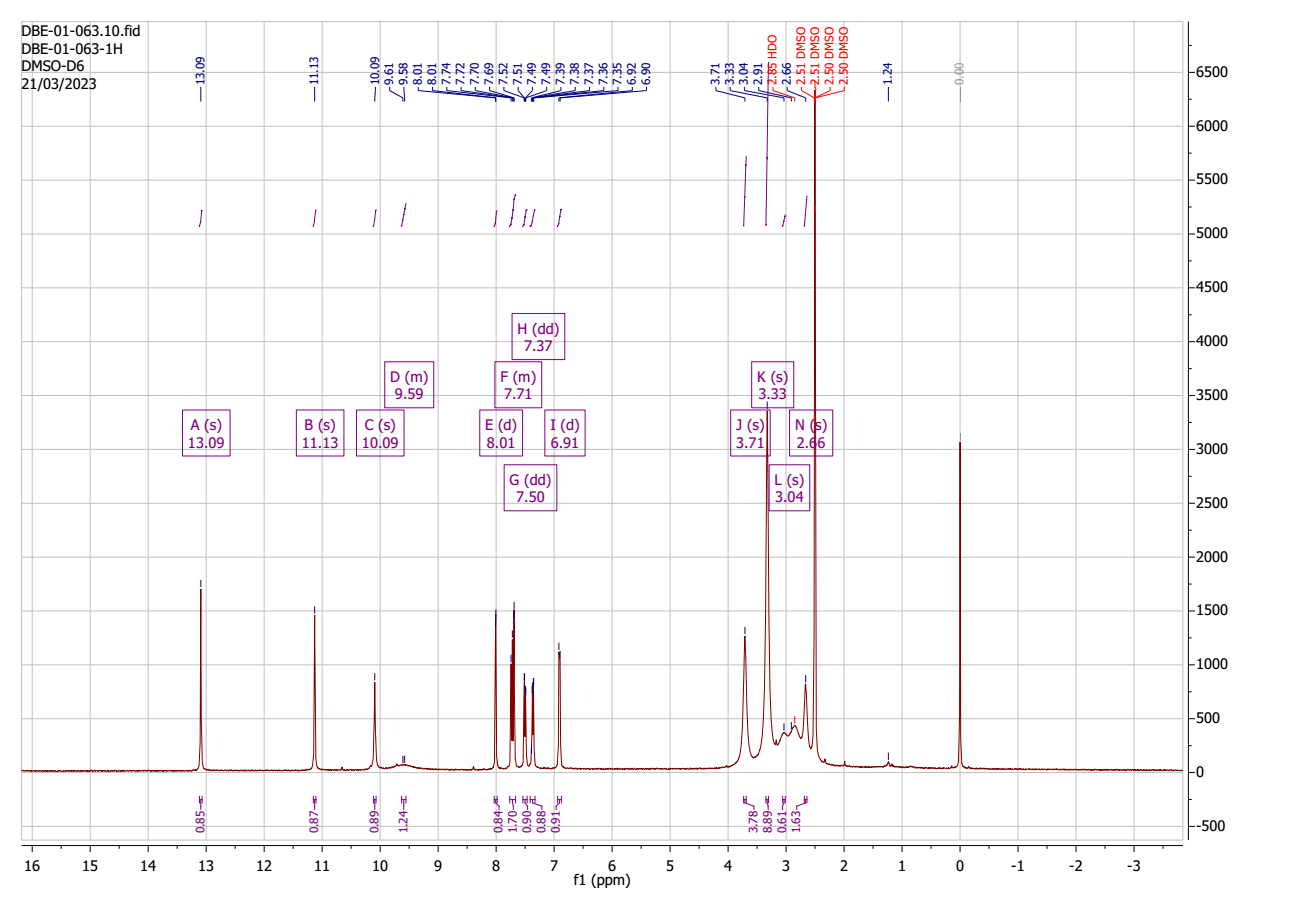


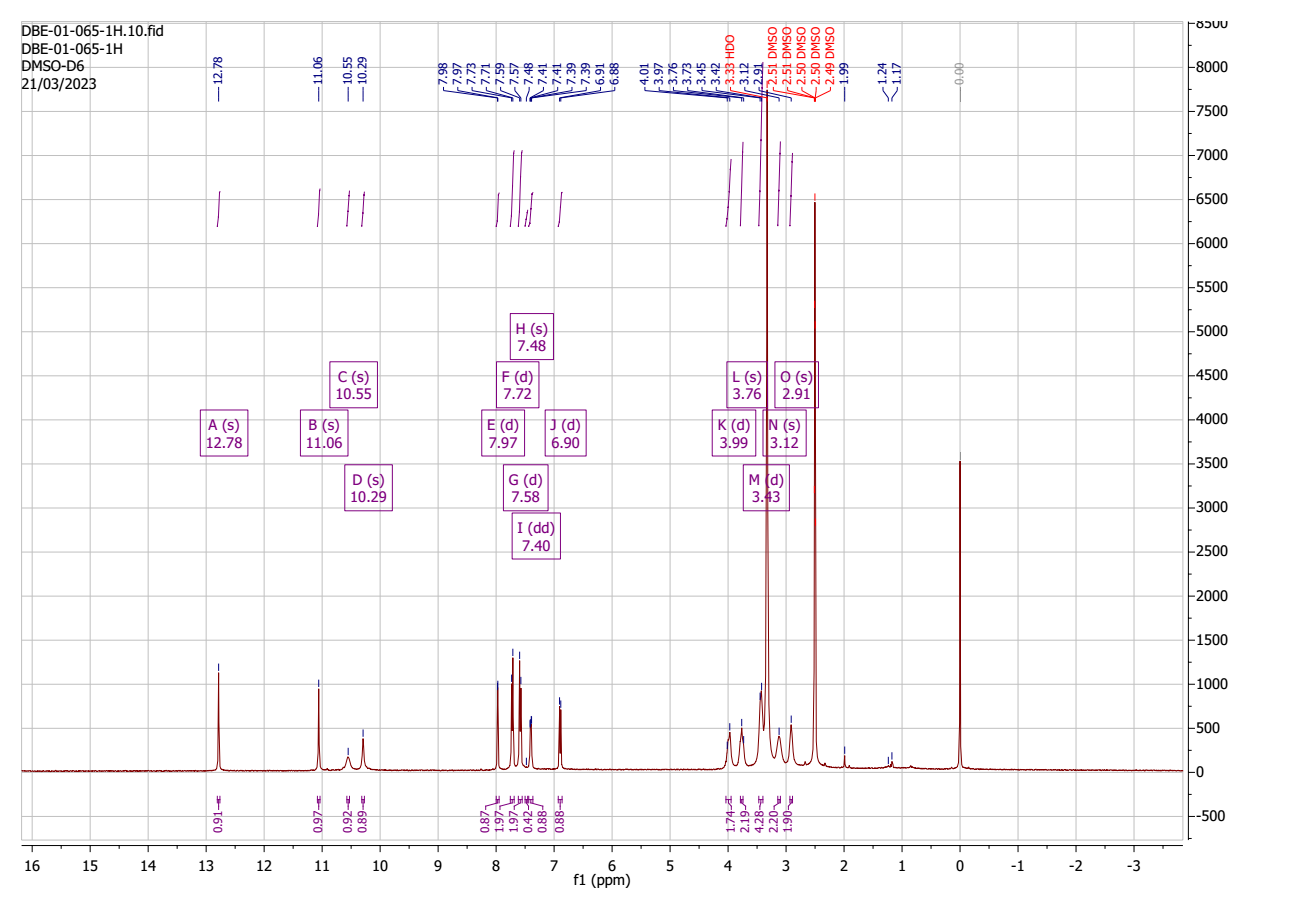


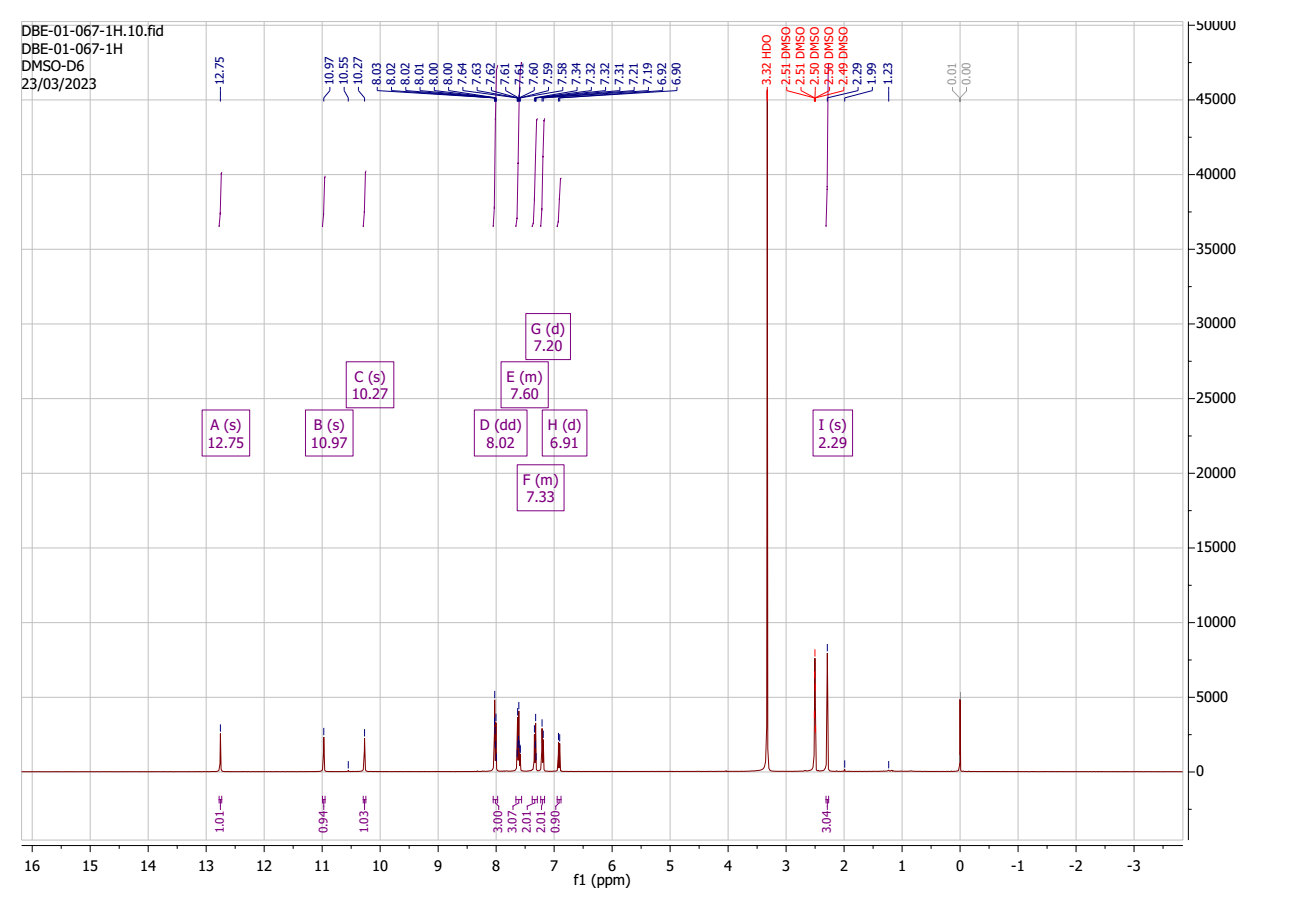


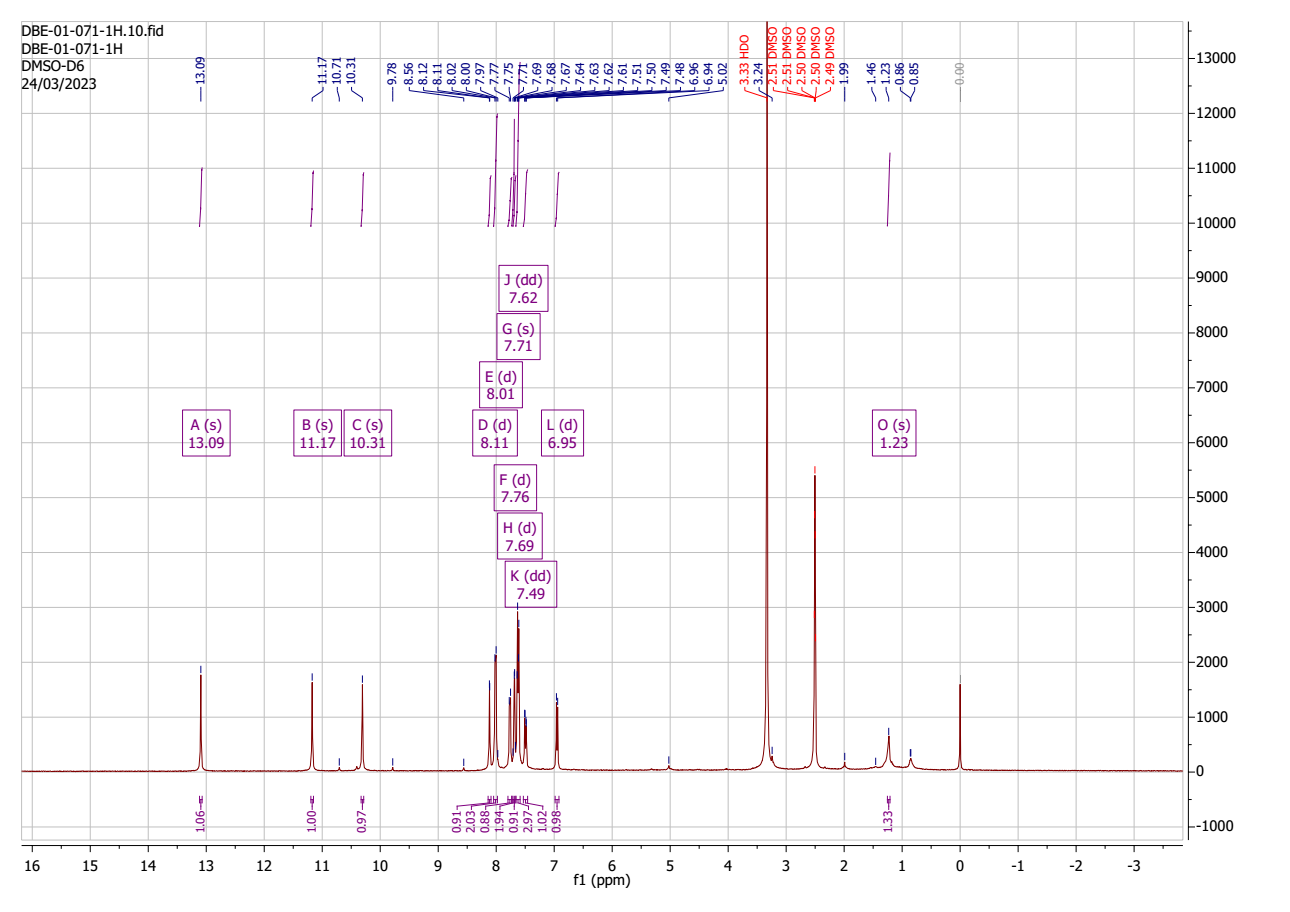


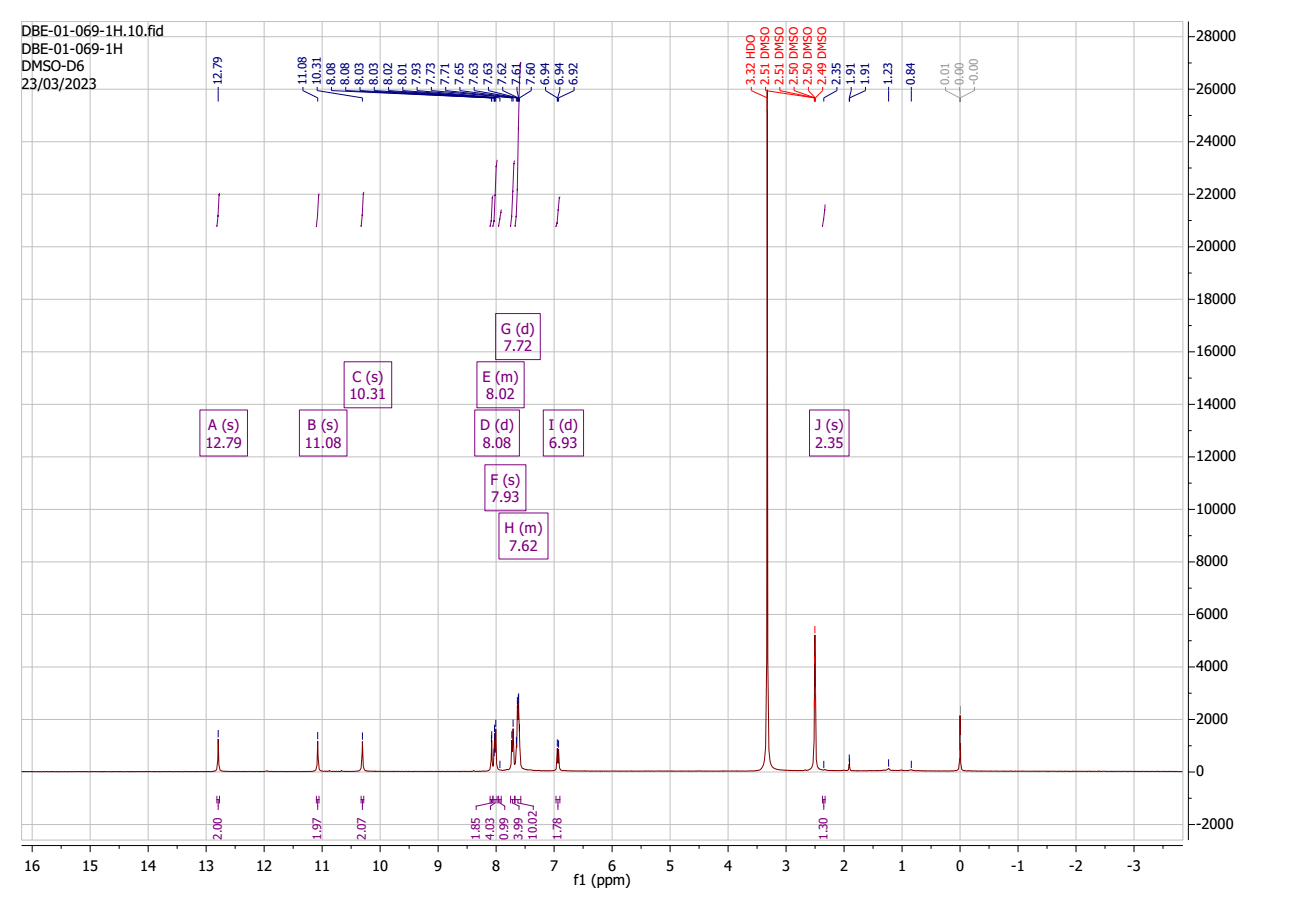


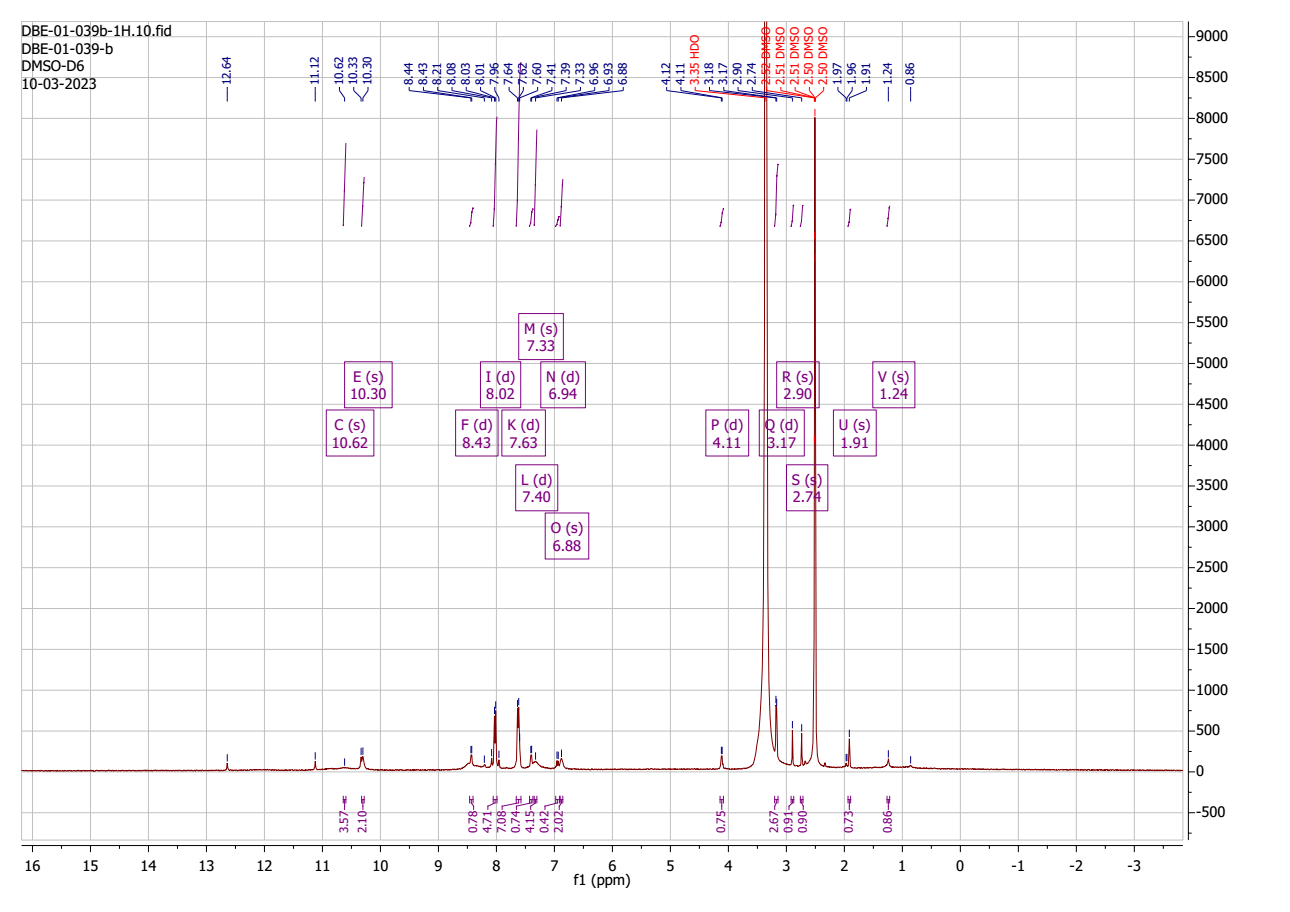

Supplement: Supplementary file 1 — Supplementary Data [file 44_2024_3201_MOESM1_ESM.docx]
